# A first principles based prediction of electronic and nonlinear optical properties towards cyclopenta thiophene chromophores with benzothiophene acceptor moieties

**DOI:** 10.1038/s41598-024-64700-6

**Published:** 2024-06-17

**Authors:** Saadia Haq, Muhammad Khalid, Ajaz Hussain, Muhammad Haroon, Saad M. Alshehri

**Affiliations:** 1 Institute of Chemistry, Khwaja Fareed University of Engineering and Information Technology, Rahim Yar Khan, 64200 Pakistan; 2 Centre for Theoretical and Computational Research, Khwaja Fareed University of Engineering and Information Technology, Rahim Yar Khan, 64200 Pakistan; 3 Institute of Chemical Sciences, Bahauddin Zakariya University, Multan, 60800 Pakistan; 4 Department of Chemistry and Biochemistry, Miami University, Oxford, OH USA; 5 Department of Chemistry, College of Science, King Saud University, 11451 Riyadh, Saudi Arabia

**Keywords:** Cyclopenta-thiophene, Push–pull architecture, Molecular engineering, DFT study, NLO, Chemistry, Materials science, Optics and photonics

## Abstract

In the current work, organic cyclopenta-thiophene (CPT) based derivatives (**FICR** and **FICD1**–**FICD5**) were designed by the modulation of end-capped acceptor group of the reference molecule i.e., **FICR**, to explore their nonlinear optical (NLO) response. The effect of terminal acceptor and donor groups in the tailored compounds was explored by using DFT based quantum calculations. The UV–Vis analysis, frontier molecular orbitals (FMOs), transition density matrix (TDM), natural bond orbitals (NBOs), density of states (DOS), nonlinear optical (NLO) analyses were performed at M06/6-311G(d,p) functional. The LUMO–HOMO band gaps of **FICD1**–**FICD5** were found to be smaller (1.75–1.92 eV) comparative to **FICR** (1.98 eV). Moreover, the global reactivity parameters (GRPs) were correlated with the results of other analyses. **FICD2** and **FICD5** with lowest band gap 1.73 and 1.75 eV showed less hardness (0.86 and 0.87 eV, respectively), high softness (0.58 and 0.57 eV^−1^), and larger absorption spectrum (815 and 813 nm) in gaseous phase and (889 and 880 nm) in solvent phase among all entitled compounds. All the designed chromophores (**FICD1**–**FICD5**) demonstrated a significant NLO response as compared to **FICR**. Particularly, **FICD2** and **FICD5** exhibited the highest average linear polarizability (<*α*>) [2.86 × 10^−22^ and 2.88 × 10^−22^ esu], first hyperpolarizability (*β*_tot_) (8.43 × 10^−27^ and 8.35 × 10^−27^ esu) and second hyperpolarizability (*γ*_tot_) (13.20 × 10^−32^ and 13.0 × 10^−32^ esu) values as compared to the other derivatives. In nutshell, structural modeling of CPT based chromophores with extended acceptors, can be significantly utilized to achieve potential NLO materials.

## Introduction

The most demanding task in present-day research is the structural modeling of sophisticated materials to generate specific non-linear optical (NLO) responses. There is an accelerating demand for the production of high-speed NLO materials owing to their enormous use in material sciences, chemical dynamics, surface interface sciences, optical computing^1^, fiber optics, data transformation and dynamic image processing^2,3^. The production and design of NLO materials has been a prominent area of theoretical and experimental research^4^.

Solid inorganic materials like LiNbO_3_ and KH_2_PO_4_ were the primary research area in NLO, but the attention was shifted to organic systems due to several significant reasons. Following the invention of simple harmonic generation (SHG) in organic compounds in 1965, the chemists involved many fruitful studies to create novel organic compounds with distinctive NLO characteristics^5,6^. Over the past two decades, organic materials have emerged as promising candidates for NLO applications, owing to their refractive index dispersion, high structural versatility, swift response rates and optical spectra in comparison to inorganic compounds^7^. In addition, they display great stability in the visible spectrum, natural softness, exceptional optical transparency, flexibility of their chemical structure due to diverse functional groups, wide variety of production processes, electro-optic switching, the generation of terahertz (THz) waves and frequency doubling^8^. Donor-π-acceptor (D-π-A) architecture plays a crucial role in achieving remarkable NLO responses^9^. There is a spatial separation of electrons in D-π-A systems, wherein, rich (D) and electron-deficient (A) moieties are interlinked by a π-conjugated bridge. The π-bridge between the donor and acceptor moieties provides a pathway for delocalized π-electrons^10^. Extending the conjugation length increases the probability of electronic transitions. This architecture exhibits intramolecular charge transfer (ICT) upon photoexcitation, where an electron is transferred from the donor to the acceptor through the π-bridge. This ICT process leads to significant changes in the molecular dipole moment, resulting in large second and third order NLO effects^11^. An effective push–pull mechanism has been established by earlier researchers using of D-*π*-A compounds tailored with different acceptors^12,13^. These push–pull arrangements affect charge distribution, broaden the wavelength range, intensify asymmetric electron distribution and narrow the energy band gap (*E*_LUMO_-*E*_HOMO_), hence increasing the NLO response^14,15^. This architecture is also utilized in the design of organic molecules, polymers, and materials as used in photonics and optoelectronics^16^. Thiophene-based compounds are considered as promising candidates, particularly when engineered with a push–pull architecture^17^. This structural design involves electron-donating and electron-withdrawing groups strategically positioned along the molecular backbone, creating a strong ICT effect. Such a configuration facilitates efficient delocalization of electron density, leading to NLO responses^18–21^. In current era, non-fullerene (NF) based compounds received a significant attention in the area of photonics due to (i) isotropic electron transference (ii) effective electron delocalization between D and A species, (iii) completely conjugated structure, and (iv) electronegativity^22^. Additionally, NFs materials, characterized by planar structures and tunable energy gaps, demonstrate significantly enhanced stability than other organic systems^23,24^. By utilizing the stronger D and A components in NFs, can enhance the NLO characteristics by developing an appropriate *π*-conjugated system.

**Figure 1:**
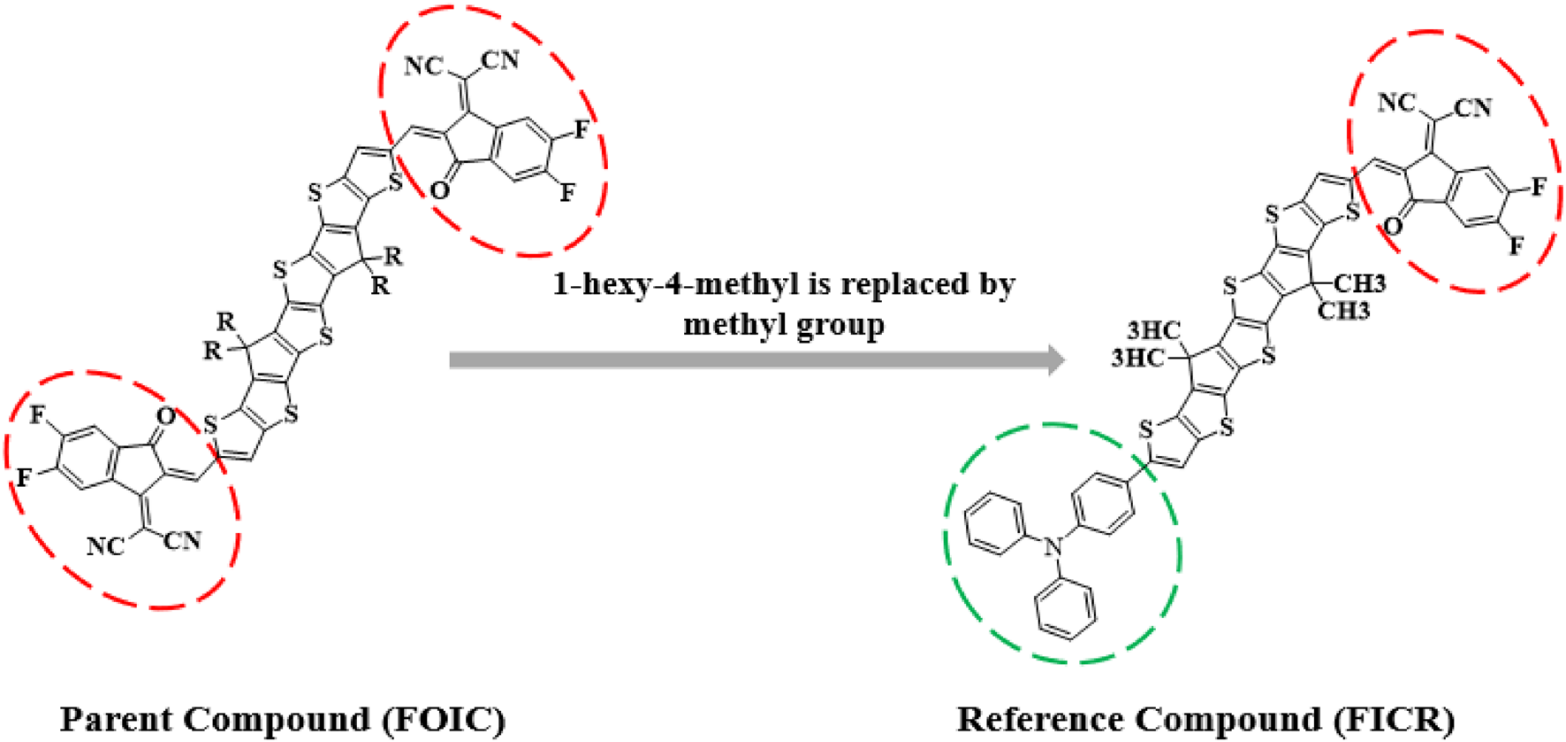
Structural modulation of parent compound (**FOIC**) into the reference compound (**FICR**).

Le and coauthor have designed and synthesized a fused tris-thienothiophene (3TT) building units having molecular packing ability and strong electron-donating effect to achieve efficient NLO properties. The chromophores having three thieno[3,2-b]thiophene (TT) units combined with two cyclopentadienyl (CP) rings, resulting structures exhibit greater planarity, along with an extended electron delocalization length compared to a single thiophene ring. Moreover, increasing the length of conjugation can enhance the NLO characteristics, and a more rigid backbone can prevent bond rotation, thereby improving molecular packing^25^. The structure–property relationship exploited that the choice of ideal length of *π*-conjugation enhanced the NLO response^26^. Therefore, an elongated *π*-bridge improves the conjugation, which eventually raises the nonlinearity of the system. Moreover, its light harvesting ability results in red shifted absorption bands^27^. The incorporation of electronegative substituents such as cyano and nitro groups at the acceptor components of NF structures may also significantly influence their electronic characteristics^28^. Therefore, keeping in mind the prior discussion, in this study an organic fullerene free cyclopentathiophene (CPT) based compound is selected as parent compound (**FOIC**)^29^ comprising A**–***π***–**A framework. In the parent compound (**FOIC**), the bulky 1-hexyl-4-methyl groups attached to the π-spacer have been replaced with methyl (-CH_3_) groups to decrease computational expenses and eliminate steric hindrance arising from the large alkyl chains (Fig. 1). Hence, we designed five compounds (**FICD1-FICD5**) by altering its architecture from A-*π*-A to D-*π*-A and determined their NLO response. The aniline moiety has been selected as a donor owing to its simple, planar, and rigid structure and good electron-donating capabilitywhich enhances the optical nonlinearity of the system^30^. Literature data exploited that this is the first comprehensive DFT work to elaborate the NLO and electronic properties of **FOIC** based molecules. The main objective of this research article is to identify prospective materials which possess promising *β*_tot_ and *γ*_tot_ amplitudes. It is anticipated that these **FOIC** based molecules would be utilized as significant NLO materials.

### Computational procedure

Current quantum chemical calculations were executed in chloroform media using Gaussian 09 program^31^. For DFT based investigations; molecular structure input and visualizing output files of the entitled systems were generated by using the GaussView 6.0^32^ and Chemcraft software^33^. The M06^34^ functional along with 6–311 G(d,p)^35^ basis set to accomplished current chemical calculations. The M06 functional, coupled with the 6-311G(d,p) basis set, has demonstrated excellent performance and shown a strong correlation with experimental data in numerous studies^36,37^. Therefore, we also used above mentioned functional and basis set in our study. At first, all the geometries were optimized in order to get true minima structures. The NBO 5.0 program^38^ was utilized for NBOs analysis *i.e.,* calculating the energy of stabilization, find out intramolecular charge transfer (ICT) among various components (D, *π*, and A) of reference compound (**FICR**) and the investigated chromophores (**FICD1-FICD5**). The TD-DFT^39^ is one of the most widely used method for the calculation of excitation energies (UV–Vis) and FMOs analyses because of its reasonable agreement with experimental data. For this reasons, TD-DFT was used for (UV–Vis) and FMOs analyses in this study. The FMOs investigation was performed to calculate the energy gap between HOMOs and LUMOs^40^. The DOS analysis was also carried out to determine the charge density of the studied compounds by using PyMOlyze 2.0 program^41^. All the graphs were developed via using Origin 8.0 software^42^. The other software’s such as Multiwfn^43^, GaussSum^44^ and Avogadro^45^ were employed for the interpretation of results from output files.

## Results and discussion

**Figure 2:**
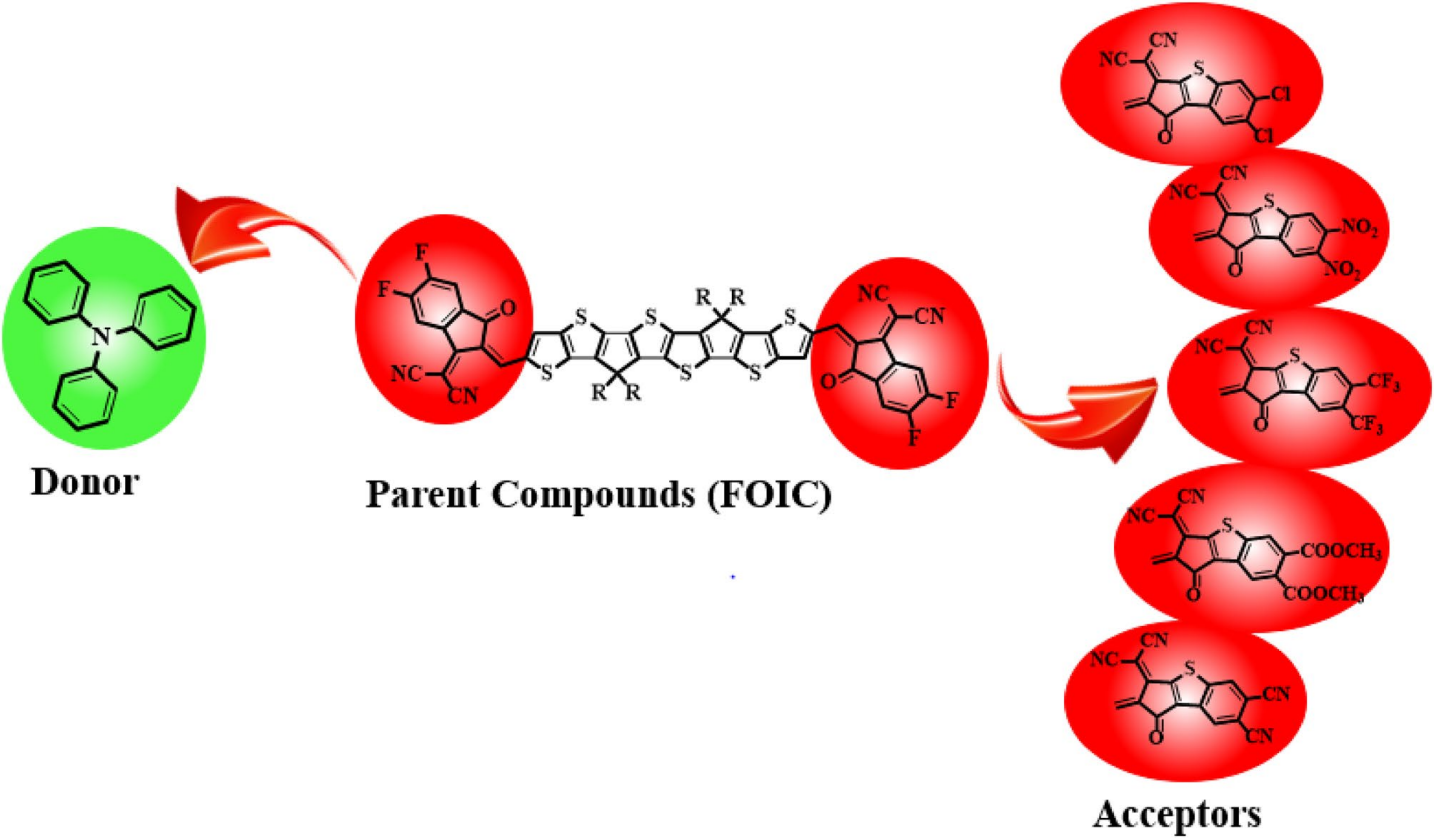
Sketch map of **FICR** and its derivatives **(FICD1-FICD5)**.

**Figure 3:**
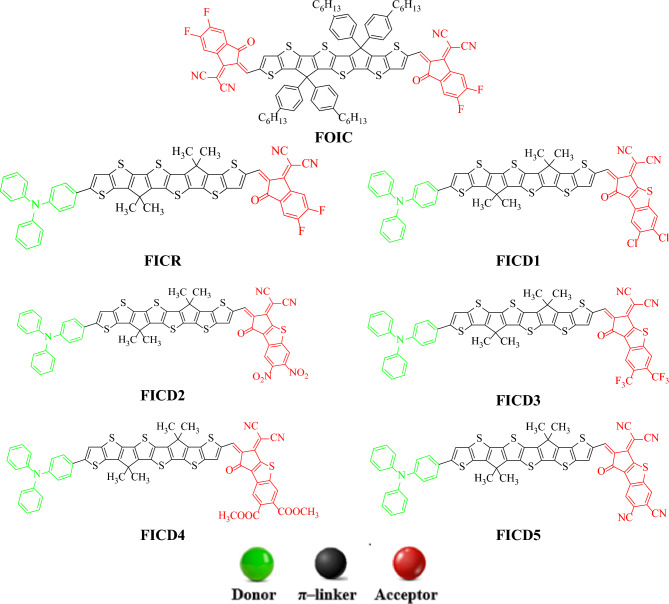
Chemdraw structures of the reference (**FICR**) and the designed derivatives (**FICD1-FICD5**).

**Figure 4:**
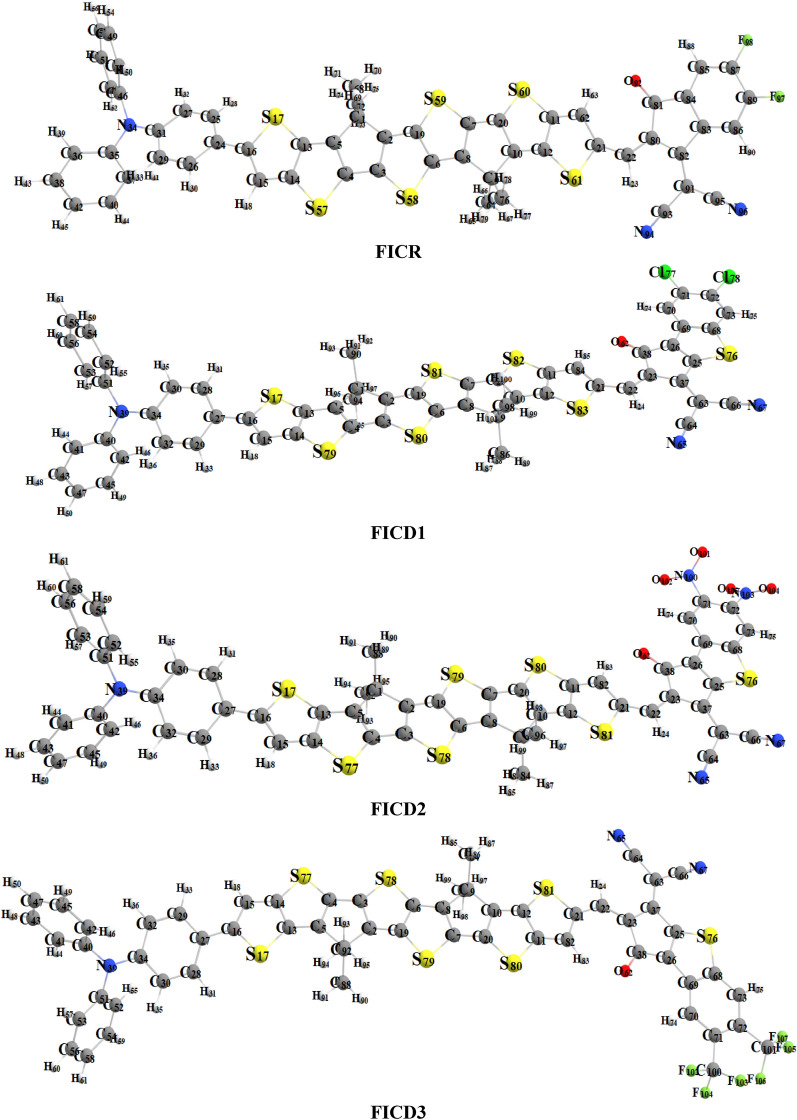

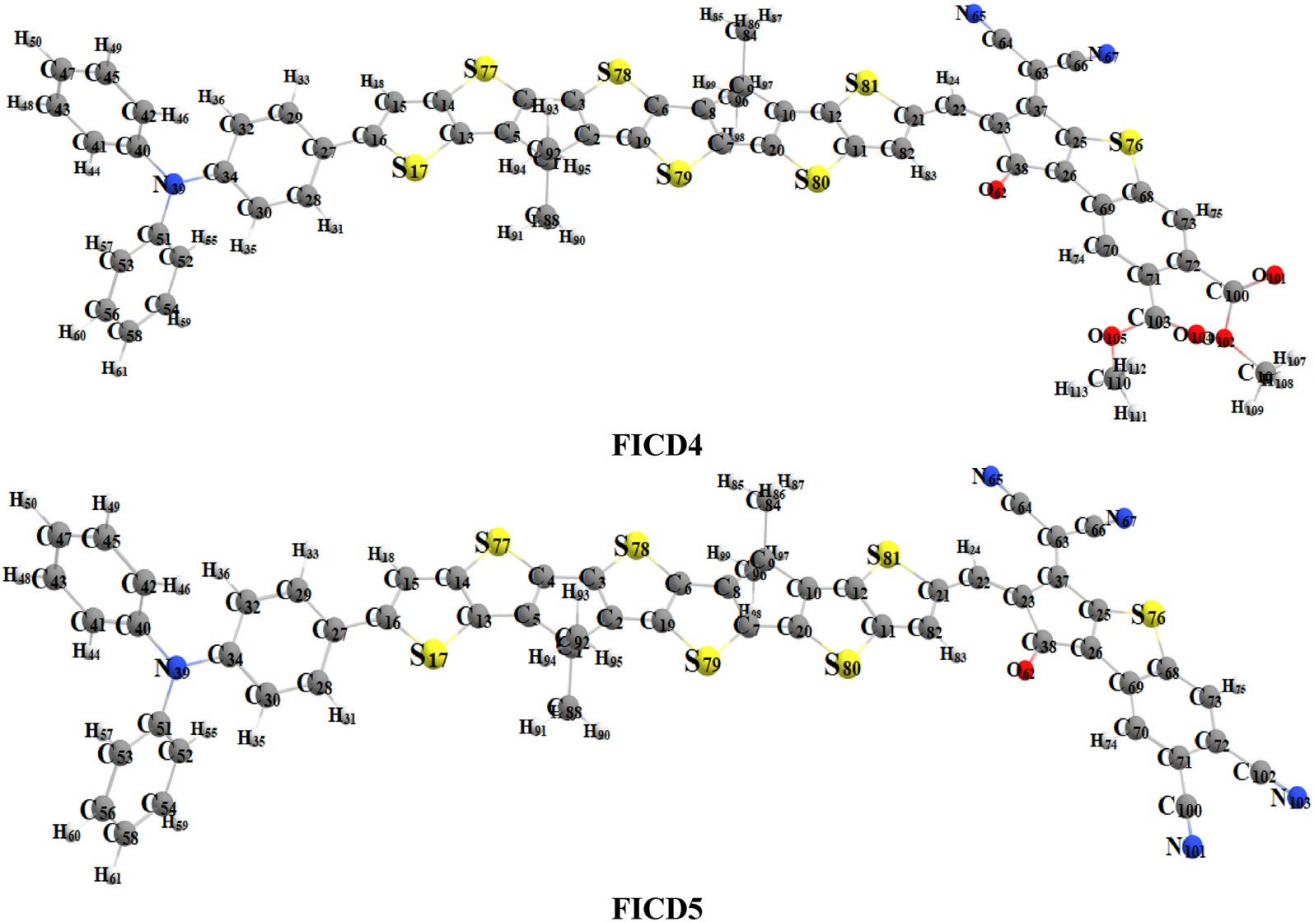
Optimized structure of the reference (**FICR**) and designed chromophores (**FICD1-FICD5**).

The present research is focused on designing a series of CPT based compounds (**FICR** and **FICD1-FICD5**) for NLO materials. For this purpose, already synthesized parent molecule (**FOIC**)^29^ with A**–***π***–**A type configuration is taken and by its some structural alteration the reference compound (**FICR**) is designed as illustrated in Fig. 1. To achieve a simple push–pull, D–*π*–A architecture, one of the peripheral acceptor of **FOIC** is exchanged with a donor moiety *i.e.,* 4-methyl-*N*-phenyl-*N*-(*p*-tolyl)aniline, while other terminal acceptor is exchanged as with benzothiophene (BT) based acceptors in order to obtain **FICR** (Fig. 2). In the designed molecules (**FICD1-FICD5**), two chloro (-Cl), two nitro (-NO_2_), two trifluoromethyl (-CF_3_), two methyl acetate (-COOCH_3_) and two cyano (-CN) groups are substituted to BT acceptor moiety (Fig. 3). Cartesian coordinates are given in Tables S1-S6. The optimized structures of the entitled compounds are presented in Fig. 4. To study the impact of structural designing on NLO properties, DFT/TD-DFT computations are performed to calculate the transitions of NBOs, spectral absorption, HOMO/LUMO band gaps, ⟨*a*⟩, *β*_tot_ and *γ*_tot_. It is expected that this research will be significant in the field of NLO and will inspire experimental researchers to synthesize these chromophores with enhanced NLO responses.

### Natural bond orbitals (NBOs) analysis

The electronic charge transfer and intramolecular charge delocalization from HOMOs to LUMOs in the *π*-conjugated network can be efficiently explained by NBOs investigation^46,47^. It demonstrate efficiency in interpreting orbital interactions within bonds, molecular stability, hyper-conjugative interactions, and hybridization of the studied compounds. Second-order perturbation theory is used to calculate the stabilization energy^48^ of the molecules by employing Eq. 1.

1 $$E^{(2)} = q_{i} \frac{{(F_{i,j} )^{2} }}{{\varepsilon_{j} - \varepsilon_{i} }}$$

Here, *i* and *j* indicate donor and acceptor groups, while *E*^(2)^ is energy of stabilization. Moreover, *q*_i_, *ε*_j_, *ε*_i_ and *F*_i,j_ are donor orbital occupancy, diagonal and off-diagonal NBO Fock matrix elements between the NBOs. Table 1 displays some significant selected transitions while the detailed values of the investigated compounds are illustrated in Tables S7-S12.

**Table 1:** The representative NBOs values for FICR and FICD1-FICD5.

Compounds	Donor (*i*)	Type	Acceptor (*j*)	Type	*E* ^(2)^ [kcal/mol]	*E*(*j*)-*E*(*i*) [*a.u*]	*F*(*i,j*) [*a.u*]
**FICR**	C4-S57	σ	C13-S17	σ*	0.51	0.90	0.02
	C10-C20	σ	C12-S61	σ*	8.58	0.92	0.08
	C93-N94	π	C82-C91	π*	0.92	1.65	0.03
	C21-C62	π	C22-C80	π*	31.18	0.30	0.09
	S57	LP(1)	C3-C4	σ*	0.92	1.18	0.03
	S61	LP(2)	C11-C12	π*	28.29	0.24	0.08
**FICD1**	C4-S79	σ	C13-S17	σ*	0.51	0.90	0.02
	C10-C20	σ	C12-S83	σ*	8.57	0.92	0.08
	C51-C53	π	C40-C42	π*	0.53	0.30	0.01
	C21-C84	π	C22-C23	π*	29.69	0.31	0.09
	S82	LP(1)	C11-C84	σ*	0.62	1.24	0.03
	S83	LP(2)	C11-C12	π*	28.01	0.25	0.08
**FICD2**	C4-S77	σ	C13-S17	σ*	0.51	0.90	0.02
	C10-C20	σ	C12-S81	σ*	8.62	0.92	0.08
	N103-O105	π	C72-N103	π*	0.93	1.39	0.04
	C21-C82	π	C22-C23	π*	31.64	0.30	0.09
	S80	LP(1)	C11-C82	σ*	0.63	1.25	0.03
	S81	LP(2)	C11-C12	π*	28.18	0.24	0.08
**FICD3**	C4-S77	σ	C13-S17	σ*	0.51	0.90	0.02
	C10-C20	σ	C12-S81	σ*	8.62	0.92	0.08
	C51-C53	π	C40-C42	π*	0.50	0.30	0.01
	C21-C82	π	C22-C23	π*	30.5	0.30	0.09
	F102	LP(1)	C71-C100	σ*	0.51	1.46	0.03
	S81	LP(2)	C11-C12	π*	28.07	0.24	0.08
**FICD4**	C4-S77	σ	C13-S17	σ*	0.51	0.90	0.02
	C10-C20	σ	C12-S81	σ*	8.58	0.92	0.08
	C51-C53	π	C40-C42	π*	0.50	0.30	0.01
	C21-C82	π	C22-C23	π*	29.64	0.31	0.09
	O105	LP(1)	C110-H111	σ*	0.52	0.96	0.02
	O105	LP(2)	C103-O104	π*	50.34	0.37	0.12
**FICD5**	C4-S77	σ	C13-S17	σ*	0.51	0.90	0.02
	C72-C102	σ	C102-N103	σ*	8.93	1.62	0.11
	C102-N103	π	C72-C102	π*	0.92	1.57	0.11
	C21-C82	π	C22-C23	π*	31.36	0.30	0.09
	S80	LP(1)	C11-C82	σ*	0.63	1.25	0.03
	S81	LP(2)	C11-C12	π*	28.12	0.24	0.08

Table 1 shows four important types of transitions such as *π* → π*, *σ* → σ*, LP → π* and LP → σ*. Among these *π* → π* are the most dominant transitions due to the presence of conjugation, while *σ* → σ* are the weak transitions due to sigma electrons. The most prominent *π* → π* transitions *π*(C21-C62) → π*(C22-C80), *π*(C21-C84) → π*(C22-C23), with 31.18 and 29.69 *kcal/mol* were observed in **FICR** and **FICD1** and 31.64, 30.50, 29.64 and 31.36 were observed in transition *π*(C21-C84) → π*(C22-C23), for **FICD2-FICD5**, respectively. The least prominent *π* → π* transitions are *π*(C51-C53) → π*(C40-C42), for **FICD1** and **FICD4** having stabilization energies 0.5 *kcal/mol*, respectively and *π*(C93-N94) → π*(C82-C91), *π*(N103-O105) → π*(C72-N103), *π*(C51-C53) → π*(C40-C42), and *π*(C102-N103) → π*(C72-C102) in case of **FICR**, **FICD2**, **FICD3** and **FICD5** with stabilization energies values of 0.92, 0.93. 0.50 and 0.92 *kcal/mol,* respectively.

In case of *σ* → σ* transitions, the highest energies of stabilization were observed to be 8.58, 8.57, and 8.93 *kcal/mol* at *σ*(C10-C20) → σ*(C12-S61), *σ*(C10-C20) → σ*(C12-S83) and *σ*(C72-C102) → σ*(C102-N103) electronic transitions for compound **FICR**, **FICD1** and **FICD5**, respectively. The transitions *σ*(C10-C20) → σ*(C12-S81) for compounds **FICD2**-**FICD4** and having highest stabilization energies: 8.62, 8.62 and 8.58 *kcal/mol*, respectively. Moreover the transitions *σ* → σ* having the least stabilization energies (0.51 *kcal/mol*) were found as *σ*(C4-S57) → σ*(C13-S17) and *σ*(C4-S79) → σ*(C13-S17) transitions for **FICR** and **FICD1**, respectively. However in case of **FICD2-FICD5** the transitions *σ*(C4-S77) → σ*(C13-S17) having least stabilization energies values of 0.51 *kcal/mol,* respectively.

Some important amplitudes of stability are also observed in lone pair transitions *i.e.,* 28.29, 28.01, and 50.34 *kcal/mol* observed in LP2(S61) → π*(C11-C12), LP2(S83) → π*(C11-C12), LP2(O105) → π*(C103-O104), for **FICR, FICD1** and **FICD4**. For compounds **FICD2**, **FICD3** and **FICD5** the transition LP2(S81) → π*(C11-C12) having the largest stabilization energies which are 28.18, 28.07 and 28.12 *kcal/mol*. Whereas, lowest stabilization energies 0.92, 0.62, 0.63, 0.51, 0.52 and 0.63 *kcal/mol* noted in electronic transitions LP1(S57) → σ*(C3-C4), LP1(S82) → σ*(C11-C84), LP1(S80) → σ*(C11-C82), LP1(F102) → π*(C71-C100), LP1(O105) → π*(C110-H111) and LP1(S80) → π*(C11-C82) for **FICR**, **FICDI-FICD5** chromophores, respectively. Some other transitions with their stabilization energies can also be seen into the Tables S7-S12. Therefore, NBOs analysis of the molecules reveal that high ICT and extended hyperconjugation play a substantial role in the stability of investigated compounds.

### Frontier molecular orbitals (FMOs) analysis

An FMO investigation was conducted to compare the electronic properties of the reference molecule (**FICR**) with its designed derivatives (**FICD1-FICD5**) along with their GRPs and DOS analyses. It is an outstanding method to determine the electronic transitions, chemical stability and reactivity of compounds^49^. Moreover, the energies of the highest occupied molecular orbitals (HOMOs) and the lowest unoccupied molecular orbitals (LUMOs) were used to determine the GRPs of the studied compounds. The energy gaps of the designed compounds were computed in chloroform utilizing the M06/6-311G(d,p) functional. (Table 2). The other orbitals and their respective energy gaps are illustrated in Tables S13-S18.

**Table 2:** Energies (eV) of frontier molecular orbitals of **FICR, FICD1-FICD5** compounds.

Compounds	*E*_*HOMO*_	*E*_*LUMO*_	Δ*E*
FICR	− 5.29	− 3.31	1.98
FICD1	− 5.26	− 3.36	1.92
FICD2	− 5.33	− 3.60	1.73
FICD3	− 5.29	− 3.44	1.85
FICD4	− 5.27	− 3.36	1.91
FICD5	− 5.29	− 3.55	1.75

Units in eV.

Table 2 showed that *E*_HOMOs_ in **FICR** and **FICD1-FICD5** were found as −5.29, −5.26, −5.33, −5.29, −5.27 and −5.29 eV*,* respectively. Similarly, calculated values for *E*_LUMOs_ are as −3.31, −3.36, −3.60,−3.44, −3.36 and −3.55 eV, respectively. In **FICD5**, HOMO is observed to be similar to **FICR** but LUMO is at lower energy (−3.55 eV), while with **FICD2**, both **FICR** and **FICD5** showed HOMO at little bit higher energy (−5.29 eV) as compared to **FICD2** (−5.33 eV). The E_gap_ of all the designed compounds (**FICD1-FICD5**) is found lower than that **FICR** indicating easily ICT in derivatives. The least energy gap is observed in case of **FICD2** (1.73 eV) and **FICD5** (1.75 eV) might be due to the presence of strong electron withdrawing moieties (-NO_2_ and -CN groups, respectively) on the acceptors as compared to the other designed chromophores. In **FICD1**, the E_gap_ was observed to be smaller than that of **FICR** but larger than **FICD5** (1.92 > 1.75 eV). The reduction in **FICD1** energy gap value as compared to **FICR** might be due to the replacement of terminal acceptor with benzothiophene acceptor having two -Cl groups. The increase in conjugation as well as inductive effect (-*I*) and resonating effect (-R) due to chloro group in **FICD1** might be the reason of reduction of energy gap in **FICD1**. The further decreased in E_gap_ in **FICD5** might be due to the replacement of -Cl groups with strong electron with drawing -CN groups. In **FICD3**, the *E*_gap_ (1.85 eV) was found smaller than **FICD4** (1.91 eV) when the -COOCH_3_ group was replaced with trifluoro (**-**CF_3_) group at the acceptor unit resulting in lesser electron-withdrawing capability. Table 2 showed that, all the designed derivatives have smaller band gap as compared to the reference. The overall descending order of band gap in **FICR** and **FICD1**-**FICD5** was found as : **FICR** > **FICD1** > **FICD4** > **FICD3** > **FICD5** > **FICD2**.

**Figure 5:**
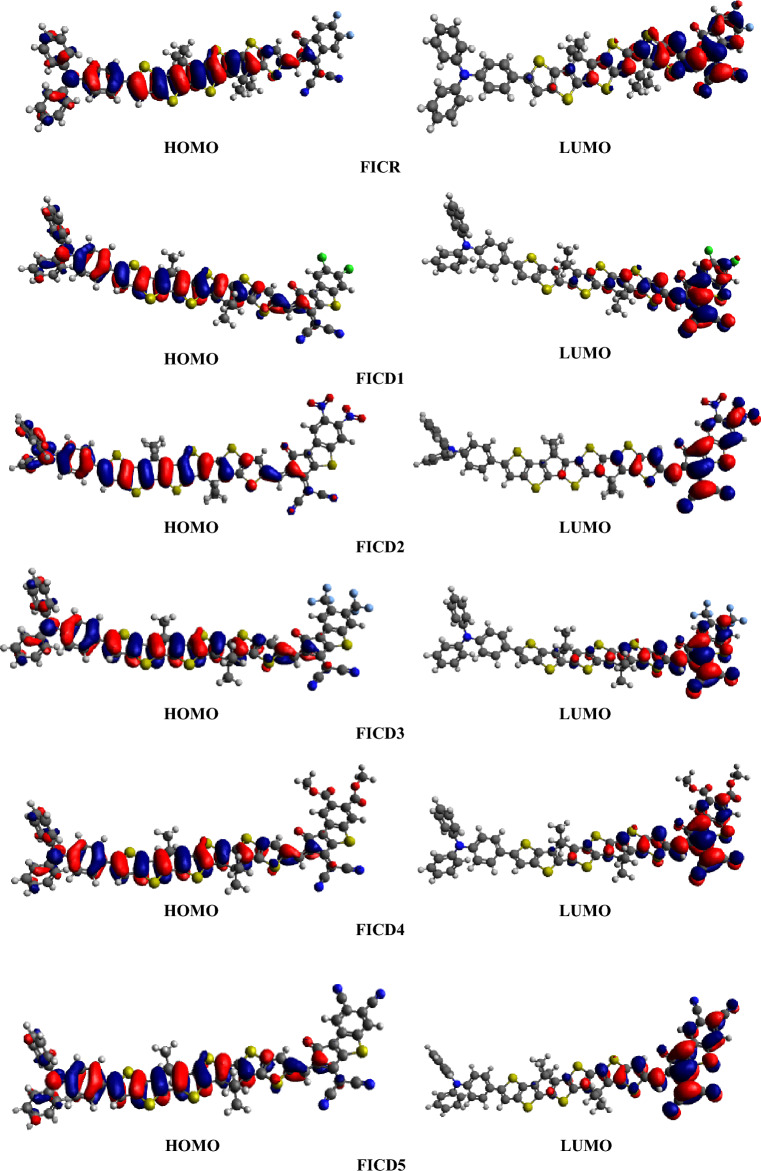
HOMOs and LUMOs representation of compounds **FICR** and **FICD1-FICD5** at 0.02 iso value.

For the charge transfer, *E*_gap_ is a crucial factor *i.e.,* narrower the band gap, larger the charge transfer rate. The **FICD2** and **FICD5** exhibited the lowest energy gap due to the enhanced conjugation and electron withdrawing nature at acceptor caused by two -NO_2_ and two -CN strong electrons withdrawing groups resulting in the most efficient push–pull mechanism out of all the entitled compounds. The data of orbital energies can be used to depict the charge transfer analysis with the help of FMOs pictographs as shown in Fig. 5 and Figure S1. Figure 5 showed that HOMOs were primarily located over donor and *π*-spacer. In contrast, LUMOs exhibited a considerable charge density over acceptor unit across all the entitled compounds. The mesomeric effects on acceptor groups primarily involve the withdrawal of electron density from neighboring atoms through resonance delocalization. In case of **FICD2**, resonance stabilization involved the delocalization of electron density onto the oxygen atoms, resulting in a significant withdrawal of electron density from the adjacent atoms. Similarly, the mesomeric effect of cyano group was attributed to the resonance stabilization involving the carbon–nitrogen triple bond, leading to the withdrawal of electron density from neighboring atoms in **FICD5**. Overall, both -NO2 and -CN groups exert strong mesomeric effects, influencing the electronic properties and reactivity of molecules containing these acceptor groups.

Thus, a notable transfer of electronic charge is observed from donor to acceptor with the help of *π*-spacer moiety in the investigated molecules as illustrated in Fig. 5. For HOMO the charge is major located over the donor and π-spacer then in LUMO this charge is significantly moves towards the acceptors. The presence of π-spacer enhances ICT from electron-rich to electron-deficient molecules, demonstrating the effectiveness of these molecular systems as NLO materials.

### UV–Visible analysis

**Figure 6:**
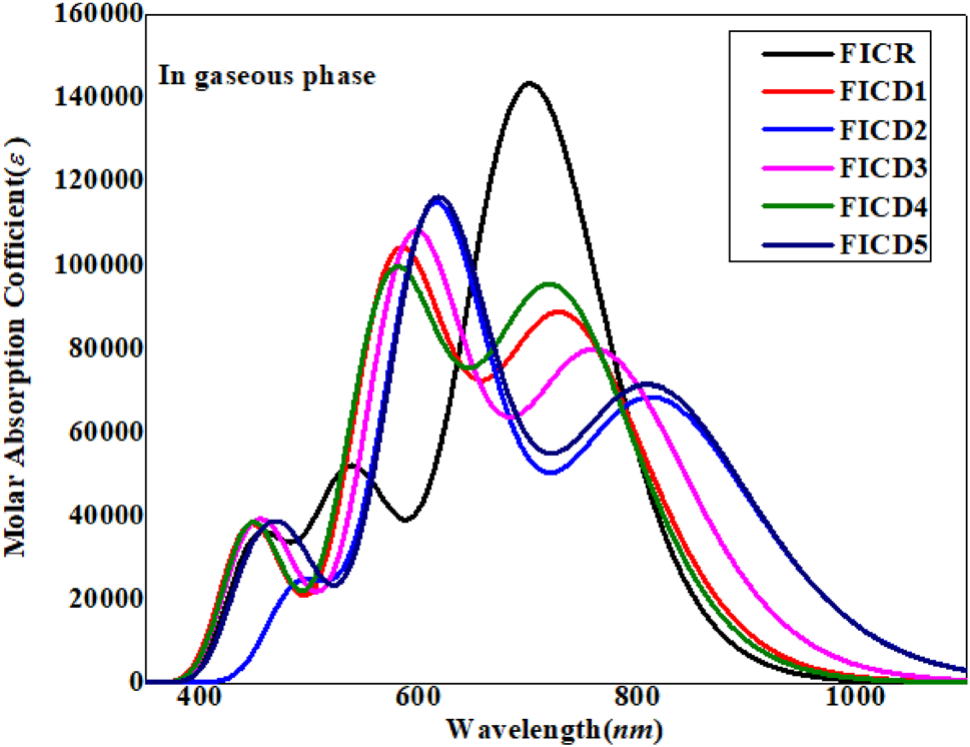
Wavelengths and molecular absorption coefficients of FICR, FICD1-FICD5 in the gaseous phase.

In order to estimate the optoelectronic properties of the entitled chromophores (**FICR** and **FICD1-FICD5**), their UV–Visible analysis is carried out in chloroform and gaseous phase by employing TD-DFT computations at the above mentioned functional^50^. This analysis provides understanding of charge transference, contributing configurations and types of electronic transitions in the investigated compounds^51,52^. Moreover, the effect of the addition of a donor and modulation of terminal acceptor group on the optical properties of compounds is also studied. By executing TD-DFT computations, six lowest singlet–singlet allowed transitions have been calculated for **FICR** and **FICD1-FICD5** and the results are tabulated in Tables S19-S24. The highest transition wavelengths (*λ*_max_) along with their major contributing percentages are shown in Table 3 and their UV–Vis spectra (gaseous phase) is depicted in Fig. 6.

**Table 3:** Excitation energies (*E*), oscillator strength (*f*_os_), wavelength ($\lambda )$ and contributions of various molecular orbitals of **FICR** and **FICD1-FICD5** in the gaseous phase.

Compounds	DFT *λ* (*nm*)	*E*(*eV*)	*f*_os_	MO contributions
FICR	701	1.77	1.98	H → L (97%),
FICD1	734	1.69	1.19	H → L (94%), H-1 → L (3%)
FICD2	815	1.52	0.93	H → L (95%), H-1 → L (3%)
FICD3	765	1.62	1.08	H → L (95%), H-1 → L (3%)
FICD4	725	1.71	1.28	H → L (94%), H-1 → L (3%)
FICD5	813	1.53	0.97	H → L (95%), H-1 → L (3%)

MO = molecular orbital, H = HOMO, L = LUMO, $\lambda$ (*nm*) = wavelength.

Table 3 clearly depicts that all the chromophores showed highest absorption in the visible region of electromagnetic spectrum. In gaseous phase, the chromophore **FICR** exhibited the *λ*_max_ with major H → L (97%) contribution at the range of 701 nm and oscillator strength (*f*_*os*_) of 1.77. All the designed chromophores **FICD1-FICD5** showed greater *λ*_max_ values at the range of 734–815 nm than **FICR**. The compound **FICD2** and **FICD5** showed the largest absorption wavelength as 815 and 813 nm and the least transition energy value of 1.52 and 1.53 eV with a smallest *f*_*os*_ of 0.93 and 0.97, respectively and HOMO–LUMO contribution of H → L (97%). Both **AICD2** and **FICD5** were observed as the most-red shifted derivatives which might be due to the presence of -NO_2_ and -CN groups on the acceptor moiety that reduced the energy gap between molecular orbitals. The *λ*_max_ (nm) of all the designed molecules is in the following ascending order: **FICR** > **FICD4** > **FICD1** > **FICD3** > **FICD5** > **FICD2**. The NLO efficiency of these materials was also calculated from excitation energy. The chromophores with lowest excitation energy exhibit bathochromic shift resulting in efficient NLO properties. The transition energy values (eV) gives the reverse order of *λ*_max_: **FICR** < **FICD4** < **FICD1** < **FICD3** < **FICD5** < **FICD2**. The UV–Visible graph in gaseous phase is displayed in Fig. 6.

**Figure 7:**
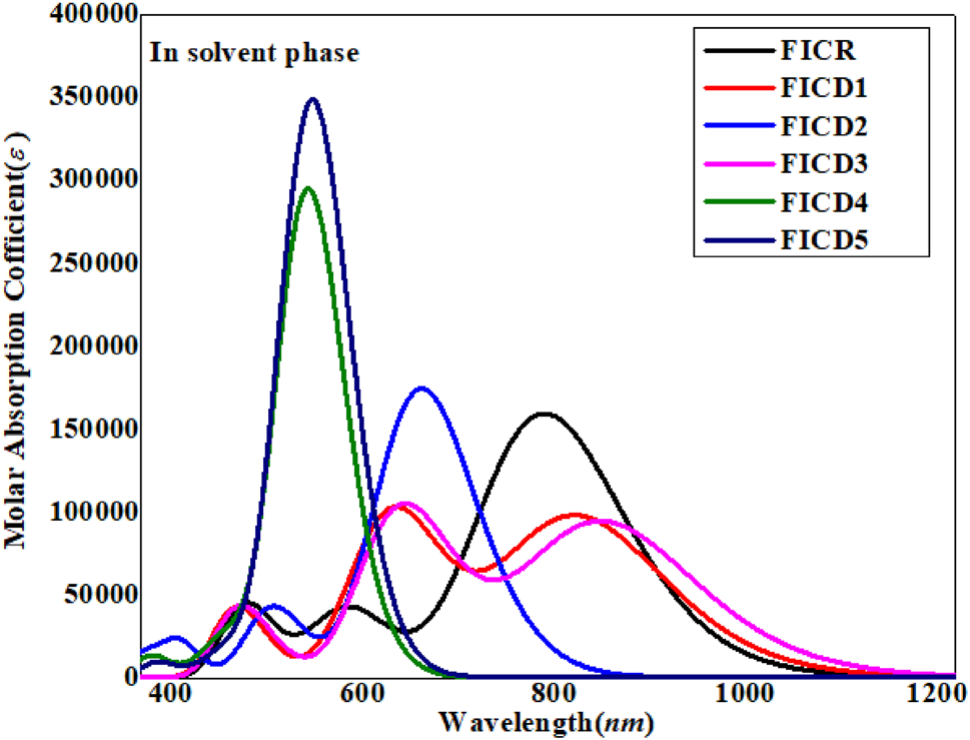
Wavelengths and molecular absorption coefficients of **FICR**, **FICD1-FICD5** in the solvent phase.

In solvent phase (chloroform), the chromophore **FICR** exhibited the *λ*_max_ with major H → L (94%) contribution at the range of 771 nm and oscillator strength of 2.20. All the designed chromophores **FICD1-FICD5** showed greater *λ*_max_ values at the range of 806–880 nm than **FICR** (Table 4). Like gas phase, in chloroform media both **FICD2** and **FICD5** chromophores showed the largest absorption wavelength as 889.29 and 879.95 nm with the least transition energy value of 1.39 and 1.41 eV, respectively and HOMO–LUMO contribution of H → L (94%). Both these compounds have strong electron withdrawing -NO_2_ and –CN groups on the acceptor moiety, hence, create a strong push–pull architecture that relatively lower the excitation energy and improved the absorption spectra towards bathochromic shift. The UV–Visible graph in solvent phase is displayed in Fig. 7. Literature study revealed that the polar medium aids in stabilizing the π–π* state alongside the n–π* state by employing a suitable electronic level^53^. Typically, the energy of compound interactions in polar media is governed by non-covalent interactions (NCIs) and polarity influences. This factor delineates dipolar interactions and hydrogen bonding, which play a significant role in stabilizing the molecules' first singlet electronic level. As solvent polarity increases, the molecule experiences a red shift^54^. The excited state is considered to be more polar than the ground state, resulting in greater stabilization of the excited state compared to the ground state in chloroform. Therefore, the current studied compounds showed wider absorption spectra in chloroform than that of gaseous phase which significantly improve the NLO properties of these compounds.

**Table 4:** Excitation energies (*E*), oscillator strength (*f*_os_), wavelength ($\lambda )$ and contributions of various molecular orbitals of **FICR** and **FICD1-FICD5** in solvent phase.

Comp	DFT λ (*nm*)	E(*eV*)	*f*_*os*_	MO contributions
FICR	771	1.61	2.20	H → L (94%), H-1 → L (3%)
FICD1	806	1.54	1.34	H → L (93%), H-1 → L (4%)
FICD2	889	1.39	1.19	H → L (94%), H-1 → L (4%)
FICD3	832	1.49	1.29	H → L (93%), H-1 → L (4%)
FICD4	806	1.54	1.36	H → L (93%), H-1 → L (4%)
FICD5	880	1.41	1.23	H → L (94%), H-1 → L (4%)

MO = molecular orbital, H = HOMO, L = LUMO, DFT = Density functional theory.

### Global reactivity parameters (GRPs)

To explore the reactivity and stability of the designed compounds, the DFT approach is employed to estimate energy values, allowing for the examination of global reactivity parameters such as global hardness (*η*), global softness (*σ*), global electrophilicity index (*ω*), electronegativity (*X*), ionization potential (*IP*), electron affinity (*EA*), and chemical potential (*μ*)^55^. The GRPs for current study chromophores were calculated by utilizing Koopmans' theorem^56^ Equations: S1-S8 given in Table S29. Either donating or accepting capability of a molecule within a system is directly linked to its ionization potential (*IP*) and electron affinity (*EA*), respectively. Electronegativity (*X*) governs a compound's capacity to attract the electron cloud towards its own nucleus^57^. Furthermore, the energy gap is closely linked to the chemical potential, hardness, and stability of a compound, whereas it has an inverse relation with reactivity^58^.

Among all the designed compounds, **FICD2** and **FICD5** are regarded as the softer, less stable, and most reactive ones owing to their higher global softness value of 0.58 and 0.57 eV, respectively. The global softness of the designed compounds follows a descending order: **FICD2**(0.58 eV) > **FICD5**(0.57 eV) > **FICD3**(0.54 eV) > **FICD1** = **FICD4**(0.52 eV) > **FICR** (0.50 eV) (Table 5). Nevertheless, the trends in global hardness values were noted to be opposite to those of global softness. The descending order of *η* is provided as follows: **FICR** (0.99 eV) > **FICD4** (0.96 eV) > **FICD1** (0.95 eV) > **FICD3** (0.92 eV) > **FICD5** (0.87 eV) > **FICD2** (0.86 eV). The *ΔN*_max_ values reveal that the **FICD2** and **FICD5** compounds have the highest value at 5.18 and 5.06 eV, demonstrating a constant charge transfer throughout these molecules. The **FICD2** and **FICD5** were predicted to be the most favorable compound as compared to the others owing to their maximum softness value of 0.58 and 0.57 eV and lowest hardness value of 0.86 and 0.87 eV, respectively. Compounds (**FICD2** and **FICD5)** with lower softness values exhibit higher polarizability with grater ICT might be consider as efficient materials for NLO applications.

**Table 5:** Global reactivity descriptors of entitled compounds (FICR and FICD1-FICD5).

Compounds	*IP*	*EA*	*X*	*η*	*μ*	*ω*	*σ*	Δ*Nmax*
FICR	5.29	3.31	4.30	0.99	−4.30	9.32	0.50	4.34
FICD1	5.27	3.35	4.31	0.95	−4.31	9.72	0.52	4.51
FICD2	5.33	3.60	4.47	0.86	−4.47	11.6	0.58	5.18
FICD3	5.28	3.44	4.36	0.92	−4.36	10.3	0.54	4.72
FICD4	5.27	3.36	4.31	0.96	−4.31	9.75	0.52	4.51
FICD5	5.29	3.54	4.41	0.87	−4.42	11.20	0.57	5.06

### Density of states (DOS)

**Figure 8:**
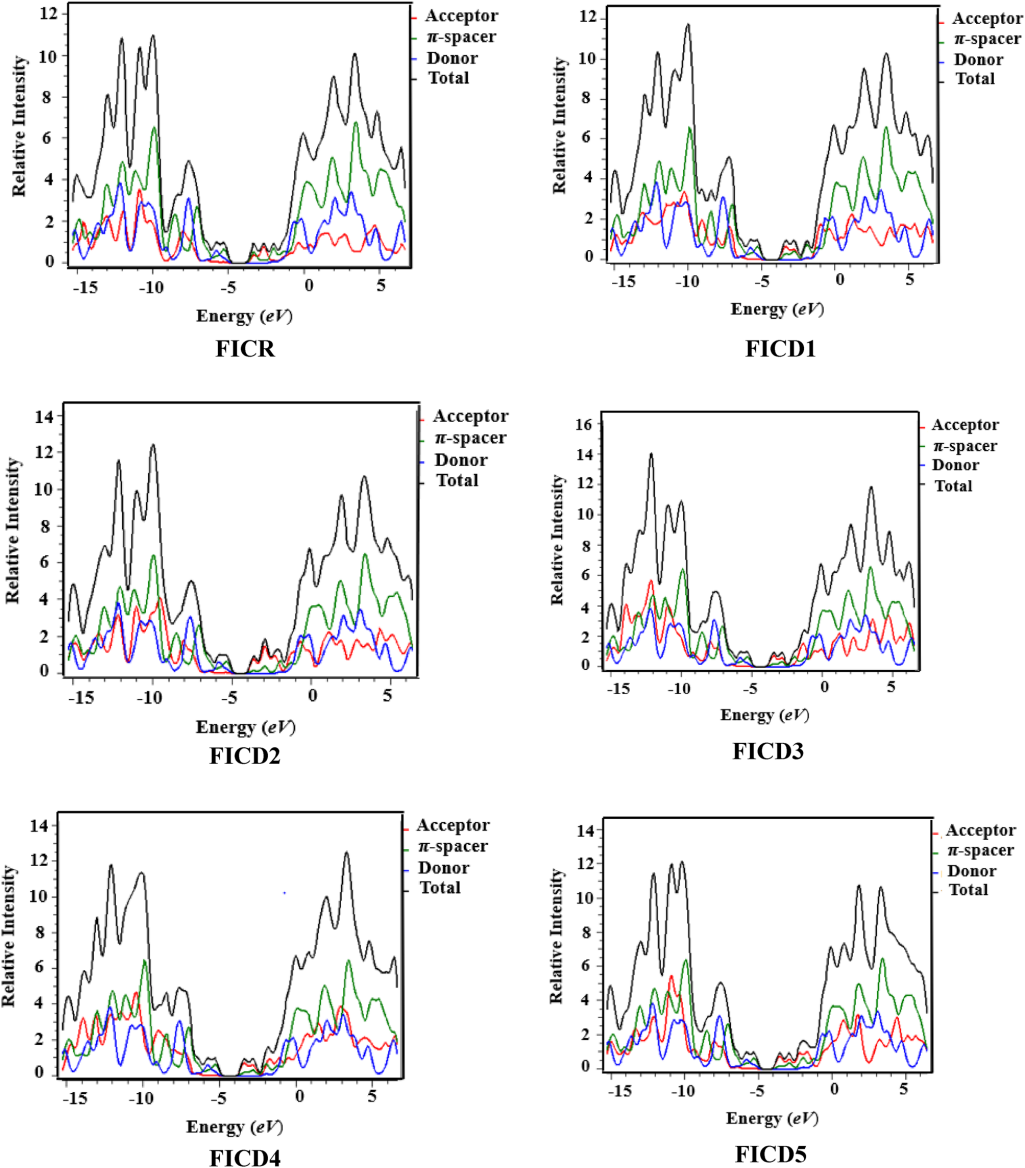
Graphical representation of DOS for **FICR** and **FICD1-FICD5**.

The DOS analysis was accomplished to validate the findings obtained by FMO analysis for the studied compounds (**FICR** and **FICD1-FICD5**)^59^ are presented in Fig. 8. On graphs, the HOMOs also called the valence band are represented at right side, whereas the LUMOs known as conduction band are shown at left side. The x-axis depicts the energy gap, which represents the separation of HOMO and LUMO^60^. To explain the findings regarding the density of states (DOS), the compounds are categorized into three segments: donor (donating group), π-spacer (linker), and acceptor (end capped acceptor unit) as demonstrated by blue, green, and red lines, correspondingly. In the reference compound, donor shows electronic charge distribution of 24.60% toward HOMO and 0.50% to LUMO. Whereas π-spacer shows that electronic charge distribution provides 69.60% to HOMO and 57.50% to LUMO. Additionally, acceptor display electronic charge distribution pattern as 5.70% toward HOMO and 42.00% toward LUMO. On the other hand, in the designed compounds (**FICD1-FICD5)**, donor display the electronic charge distribution pattern as 21.90, 24.10, 24.10, 23.10 and 26.10% to HOMO and 0.20% to LUMO. Similarly, for **FICD1-FICD5**, π-spacer distributes 72.50, 69.30, 69.70, 70.80 and 67.70% to HOMO and 30.10, 17.60, 20.70, 21.30 and 19.10% to LUMO. Likewise, acceptor shows the electronic charge distribution pattern as 5.60, 6.70, 6.20, 6.10 and 6.20% to HOMO and 69.70, 82.30, 79.10, 78.50 and 80.70% to LUMO. These findings showed that in the compounds, the electron density in the FMOs analysis of the studied compounds shows that the HOMO density is positioned over donor and π-spacer. While in the case of LUMO electron density is predominantly located over acceptor in all studied compounds.

### Transition density matrix and exciton binding energy

The TDM investigations plays an essential part in determining the amount of electronic-charge transfer within reference and designed compounds^61^. The TDM is useful for assessing charge density excitation, electron–hole pair localization and delocalization, and excited state interactions between electron-accepting and electron-donating entities^60^.

**Figure 9:**
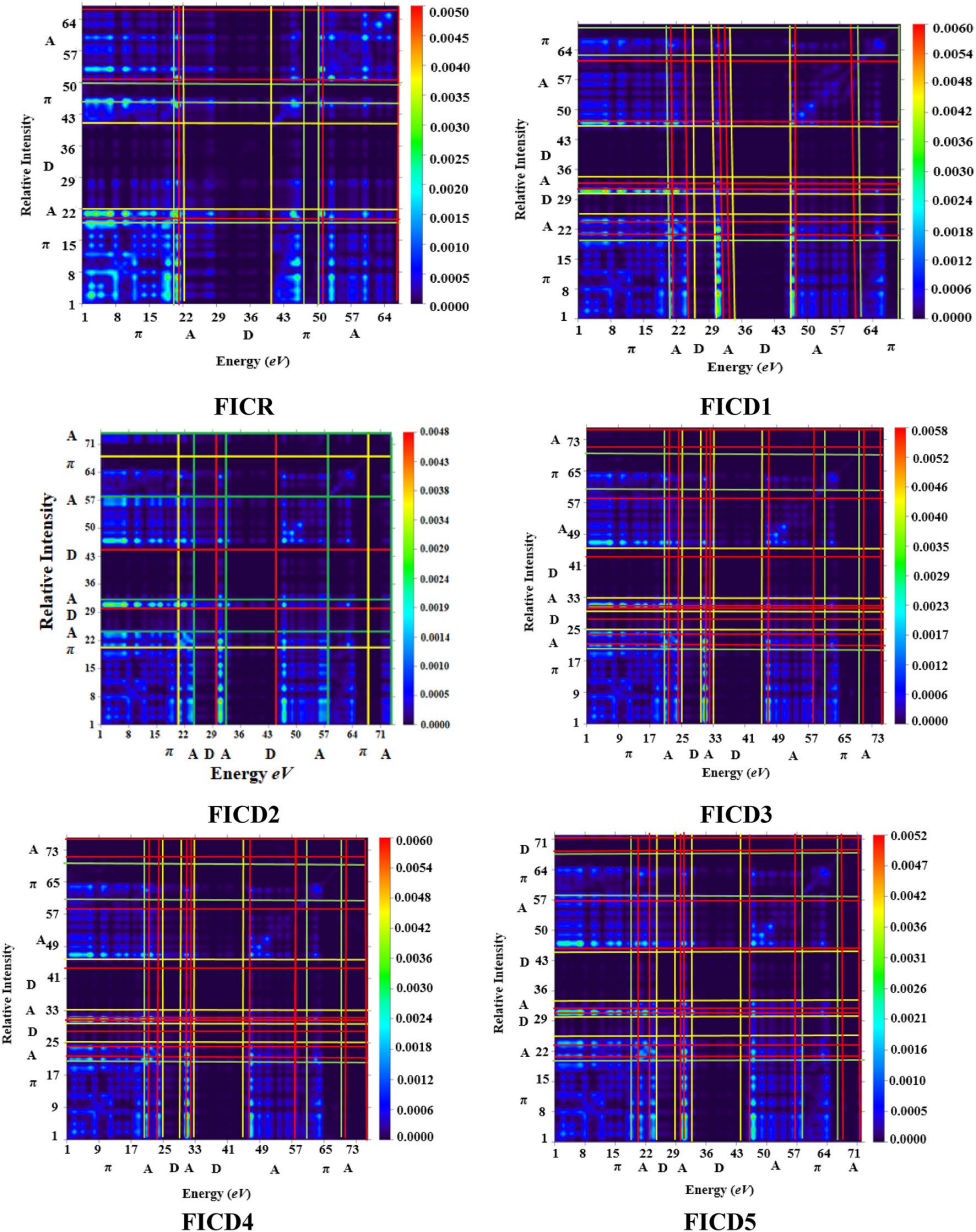
TDM graphs of compounds (**FICR** and **FICD1** to **FICD5**).

Additionally, it is also helpful in understanding transition behavior from the ground (S0) to excited state (S1)^62^. The TDM of the studied compounds was calculated using the M06/6-311G(d, p) method. The influence of the hydrogen atom is ignored in this analysis owing to its small influence on electronic transitions. The TDM heat map of all the designed compounds demonstrate the nature of transition is illustrated in Fig. 9. In this visual representation, the lower horizontal axis and the left-side vertical axis depict the atom count, while the right-side vertical axis represents the transition coefficient and electron density resulting from interactions with light. Furthermore, the green dots on the dark blue background provide a pictorial illustration of charge transfer. To understand the distribution of charge density, we divided each designed compound into three components: π-spacer (linker), donor and acceptor. As indicated in the pictographs, the highest electronic cloud was apparent on the π -linker in **FICR** and **FICD1** to **FICD5**. Although a little charge can be observed on the donor region, which successfully facilitate the shifting of electron density towards the acceptor despite trapping by incorporating electron coherence efficiently transferred from the donor to the π-linker.

Moreover, the binding energy (E_b_) serves as a crucial parameter aiding researchers in evaluating the optoelectronic characteristics of material^63^. It facilitates researchers in computing the coulombic interaction forces between the hole (*h*) and electron (*e*). At excited state, exciton dissociation has an inverse relationship with the E_b_, however there is a direct correlation between the E_b_ and the coulombic interaction between the *h* and *e*. Reduced E_b_ results in higher charge mobilities^64^. The E_*b*_ of designed chromophores **FICR** and **FICD1-FICD5** was determined using Eq. 2^65^.

2 $$E_{b} = E_{{{\text{H}} - {\text{L}}}} - { }E_{{{\text{opt}}}} { }$$

In Eq. (4), ${\text{E}}_{{{\text{H}} - {\text{L}}}} { }$ signifies energy difference between HOMO/LUMO. ${\text{E}}_{{{\text{opt}}}} { }$ represents that the minimum energy required for the initial excitation from S_0_ to S_1_, resulting in generation of electron and hole^66,67^. Table 6 displays DFT computed findings for E_b_ of the above-mentioned chromophores.

**Table 6:** Calculated *E*_*b*_ **FICR** and **FICD1-FICD5** compounds.

Compounds	*E*_*H–L*_	*E*_*opt*_	*E*_*b*_
FICR	1.98	1.61	0.37
FICD1	1.92	1.54	0.38
FICD2	1.73	2.30	−0.57
FICD3	1.85	1.49	0.36
FICD4	1.91	2.36	−0.45
FICD5	1.75	2.33	−0.58

Units in *eV.*

The E_b_ value revealed that, **FICD2** and **FICD5** demonstrated a greater extent of charge separation when compared to other derivatives. Table 6 presents the calculated values of the HOMO–LUMO gap (E_*H*_-E_*L*_), the first single excitation energies (E_opt_), and the excitation binding energies (E_b_), combined with its high current charge density, makes **FICD2** and **FICD5** a highly promising candidate for achieving exceptional efficiency in NLO compounds. The observed correlation between binding energy and TDM outcomes demonstrates a remarkable degree of charge transfer within molecules. The following compounds' binding energy values are listed in decreasing order: **FICD4 > FICD1 > FICR > FICD3 > FICD2 > FICD5**.

### Nonlinear optical (NLO) analysis

Linear polarizability (*α*) defines the potential of electric field to distort the molecular electron distribution^68^. The hyperpolarizability (*β*_tot_, *γ*_tot_, etc.) is a key parameter that characterizes the nonlinear optical behavior of molecules and atoms, encompassing a wide range of complex phenomena within the field of nonlinear optics. The first hyperpolarizability (*β*_tot_) is ascertained through the finite field method, where the proximity of an applied electric field influences the system's molecular energy. The relationship between molecular structure and NLO effects is defined by the charge transfer capability and hyperpolarizability of a substance. When a material displays a substantial NLO response, it demonstrates an elevated dipole moment, significant linear polarization, and substantial hyperpolarizability values.

The dipole moment (*µ*_tot_)^69^ of all the entitled compounds was determined by using Eq. 2.

3 $$\mu_{{{\text{tot}}}} = \left( {\mu^{{2}}_{x} + \mu^{{2}}_{y} + \mu^{{2}}_{z} } \right)^{{{1}/{2}}}$$

Similarly, the linear polarizability ⟨*a*⟩^70^ was computed with the help of Eq. 3.

4 $$\left\langle \alpha \right\rangle = {1}/{3}\left( {\alpha_{xx} + \, \alpha_{yy} + \alpha_{zz} } \right)$$

The magnitude of first hyperpolarizability *β*_tot_^71^ was measured with the help of Eq. 4.

5 $$\beta_{tot} = (\beta_{{\text{x}}}^{{2}} + \beta_{{\text{y}}}^{{2}} + \beta_{{\text{z}}}^{{2}} )]^{{{1}/{2}}}$$

where the *β*_x_ = *β*_xxx_ + *β*_xyy_ + *β*_xzz_, *β*_y=_*β*_yyy_ + *β*_xxy_ + *β*_yzz_ and* β*_z_ = *β*_zzz_ + *β*_xxz_ + *β*_yyz_.

The second hyperpolarizability *γ*_tot _^72^ was calculated by using Eq. 5.

6 $$\gamma_{tot} = \sqrt {{\upgamma }_{x }^{2} + {\upgamma }_{y}^{2} + {\upgamma }_{z}^{2} }$$

$$\gamma_{i} = \frac{1}{15 }\mathop \sum \limits_{j} (\gamma_{ijji} + \gamma_{ijij} + \gamma_{iijj} ) \quad i,j = \left\{ {x, y, z} \right\}$$

The calculated results for the electric dipole moment (*µ*_tot_), linear polarizability (< *α* >), total first order hyperpolarizability (*β*_tot_), and total second-order hyperpolarizability (*γ*_tot_), along with their respective tensors, have been presented in Tables S25-S28 for the **FICR** and **FICD1-FICD5** systems. A summary of the key discoveries can be found in Table 7.

**Table 7:** The dipole moment (*µ*_tot_), 1st order (*α*_tot_), 2nd order (*β*_tot_) and 3rd order NLO (*γ*_tot_) values of **FICR** and **FICD1**-**FICD5**.

Compounds	*µ*_*tot*_	< *α* > (× 10^−22^)	*β*_tot_ (× 10^−27^)	*γ*_tot_ (× 10^−32^)
FICR	17.87	2.61	6.42	8.14
FICD1	14.31	2.69	5.70	7.99
FICD2	19.41	2.86	8.43	13.2
FICD3	16.20	2.73	6.61	9.35
FICD4	17.59	2.76	5.85	8.16
FICD5	19.76	2.88	8.35	13.0

Dipole-moment = Debye (D); linear and nonlinear = *e.s.u.*

The dipole moment arises because of variations in electronegativity. A larger disparity in electronegativity corresponds to an increased dipole moment (*µ*_*tot*_). In the context of the **FICD5** molecule, it exhibited the highest total dipole moment (*µ*_*tot*_) of 19.76 Debye (*D*) because of the notably high electronegativity cyano groups among all derivatives. The **FICR** and **FICD1**-**FICD5** have shown the trend for dipole moment values as; **FICD1** < **FICD3** < **FICD4** < **FICR** < **FICD2** < **FICD5**. The increased electronegativity of atoms results in the attraction of charge density towards them, thereby leading to increase of linear polarizability. Consequently, the greatest linear polarizability, measured at 2.88 × 10^−22^ esu, was investigated in **FICD5**. This phenomenon can be associated with the incorporation of electronegative functional groups within the acceptor moiety. The observed linear polarizability trend of **FICR** and **FICD1–FICD5** is **FICR** < **FICD1** < **FICD3** < **FICD4** < **FICD2** < **FICD5**. In the context of the **FICD5** model, the dominant tensor component responsible for linear polarizability was oriented along the x-axis, denoted as *α*_xx_, with a magnitude of 5.94 × 10^−22^ esu. This specific tensor component plays a prominent role in determining the overall linear polarizability value.

Among the studied compounds, **FICD2** exhibited the highest first hyperpolarizability at 8.43 × 10^−27^ esu. In contrast, **FICD1** displayed the lowest first hyperpolarizability at 5.70 × 10^−27^ esu, attributed to minimal intramolecular charge transfer. Specifically, the component *β*_xxx_ = 8.41 × 10^−27^ esu. contributes significantly to the overall first hyperpolarizability value in **FICD2**. Among all the compounds, the highest second hyperpolarizability value was observed in **FICD2**, measuring 13.2 × 10^–32^ esu. However, the trend for second hyperpolarizability is as follows: **FICD1** < **FICR** < **FICD4** < **FICD3** < **FICD5** < **FICD2**. Additionally, the second hyperpolarizability value, like linear polarizability and first hyperpolarizability, was contributed by the γ_X_ tensor, amounting to 1.32 × 10^−32^ esu. The calculated dipole moment, average polarizability, second-order and third-order polarizability values of the investigated chromophores (**FICR, FICD1–FICD5**) were significantly greater than that for *para-*nitroaniline (*p*-NA)^73^, a standard molecule for the analysis of the NLO response. The γ_*tot*_ values of **FICR, FICD1, FICD2, FICD3, FICD4** and **FICD5** were determined to be (8.16 × 10^−32^, 7.94 × 10^−32^, 1.33 × 10^−31^, 9.44 × 10^−32^, 8.24 × 10^−32^ and 1.30 × 10^−31^) larger than the γ_tot_ value of *p*-NA (7.29 × 10^−36^ esu)^73^, respectively.

In literature a lot of work has been done on the NLO study of both inorganic^74^ and organic compounds^75^, however, organic compounds showed significant NLO properties. Additionally, a comparative study was made between our designed derivatives with reported findings of compounds similar analogues (**DFPPC** and **DCPPC**)^76^. The findings of designed chromophores (**FICR** and **FICD1-FICD5**) showed remarkable results in terms of linear polarizability and second hyperpolarizability values as compared to **DFPPC** and **DCPPC**. Specifically, the linear polarizability values showed that designed chromophores (**FICR** and **FICD1-FICD5**) were 100, 103, 109, 105, 106 and 110 times greater than compound **DFPPC** (2.6116 × 10^−23^ esu) and 85, 87, 92, 88, 89 and 93 times greater than compound **DCPPC** (3.0772 × 10^–23^ esu). Similarly, the nonlinear second polarizability values of designed derivatives (**FICR** and **FICD1-FICD5**) were observed to be 25,096, 24,585, 40,615, 28,769, 25,015 and 40,000 times greater than **DFPPC** (3.2455 × 10^−35^ esu) and 26,506, 26,015, 43,042, 30,425, 26,579 and 42,339 times greater than **DCPPC** (3.0708 × 10^–35^ esu).

The analysis revealed that transitioning from the A–*π*–A configuration to the D–*π*–A configuration, coupled with an increased electron-withdrawing effect, induced pronounced charge transfer and a redshift in the spectral absorption, leading to enhanced optical nonlinearity in the derivatives. Notably, the narrowing of the band gap resulting in enhanced NLO property. Both the band gap and NLO property having inverse relation where compounds with narrower band gaps typically demonstrate superior NLO performance. It is noted that, both the compounds **FICD2** and **FICD5** having the lowest energy band gaps (1.73 and 1.75 eV respectively) and highest 2nd hyperpolarizability values (13.2 × 10^−32^ and 13.0 × 10^−32^ esu respectively). Due to their substantial polarizability and hyperpolarizability values, these designed compounds hold promise as prospective candidates for NLO materials. Among them, **FICD2** and **FICD5** emerge as a particularly outstanding constituent for nonlinear optics.

## Conclusion

Herein, a series of CPT based derivatives (**FICD1–FICD5**) was designed from a NF molecule (**FICR)** by modifying the configuration from A–*π*–A to D–*π*–A. It is observed that acceptors pay a notable impact on electronic properties and to enhance the NLO response of the tailored compounds than the reference compound. Our performed analyses demonstrated that all proposed molecules experienced efficient charge transfer from donor to acceptor across a π-bridge, resulting in a lower energy gap (1.75–1.92 eV) than **FICR** (1.98 eV). The TDM and DOS data also supported the charge transfer process between HOMO and LUMO with an effective way. The **FICD1-FICD5** showed greater absorption and less transitional energy values as compared to **FICR**. The broader red-shift spectrum value (*λ*_max_ = 815 and 813 nm in gaseous phase and 889 and 880 nm in solvent phase) was exhibited by **FICD2** and **FICD5** as compared to all the other designed compounds. Moreover, NBOs analyses revealed that prolonged conjugation causes stability in the studied molecules. The significant NLO properties in terms of < *α* > (2.86 × 10^−22^ and 2.88 × 10^−22^ esu for **FICD2** and **FICD5,** respectively), *β*_tot_ (8.43 × 10^−27^ and 8.35 × 10^−27^ esu for **FICD2** and **FICD5**, respectively) and *γ*_tot_ (13.20 × 10^−32^ and 13.00 × 10^−32^ esu) were investigated as compared to **FICR** and other designed compounds. The promising NLO results for **FICD2** and **FICD5** might be obtained due to the strong electron-withdrawing groups. Effective NLO responses of current study chromophores may serve as a driving force for researchers to synthesis such efficient NLO chromophores for photonic applications.

### Supplementary Information

Supplementary Information.

## Data Availability

All data generated or analyzed during this study are included in this published article and its supplementary information files.
