# Supplementary material for: A first principles based prediction of electronic and nonlinear optical properties towards cyclopenta thiophene chromophores with benzothiophene acceptor moieties
Source: Sci Rep. 2024 Jun 17;14:13971. doi: 10.1038/s41598-024-64700-6 (PMC11183240; doi:10.1038/s41598-024-64700-6)
Supplement: Supplementary file 1 — Supplementary Information. [file 41598_2024_64700_MOESM1_ESM.docx]

**Supplementary Data**

**A First Principles Based Prediction of Electronic and Nonlinear Optical Properties towards Cyclopenta Thiophene Chromophores with Benzothiophene Acceptor Moieties**

Saadia Haq,^1,2^ Muhammad Khalid,^1,2*^ Ajaz Hussain,^3^ Muhammad Haroon^4^, Saad M. Alshehri,^5^

^1^Institute of Chemistry, Khwaja Fareed University of Engineering & Information Technology, Rahim Yar Khan, 64200, Pakistan

^2^Centre for Theoretical and Computational Research, Khwaja Fareed University of Engineering & Information Technology, Rahim Yar Khan, 64200, Pakistan

^3^ Institute of Chemical Sciences, Bahauddin Zakariya University, Multan, 60800, Pakistan

^4^Department of Chemistry and Biochemistry, Miami University, Oxford, OH, USA

^5^Department of Chemistry, College of Science, King Saud University, Riyadh 11451, Saudi Arabia

*Corresponding author E-mail addresses:

Dr. Muhammad Khalid ([muhammad.khalid@kfueit.edu.pk](mailto:muhammad.khalid@kfueit.edu.pk); [khalid@iq.usp.br](mailto:khalid@iq.usp.br))

**Table S1**: Cartesian coordinates of **FICR** compound.

| **Atom** | **X-axis** | **Y-axis** | **Z-axis** |
| --- | --- | --- | --- |

| C | 2.5552 | 0.83369 | -0.03712 |
| --- | --- | --- | --- |
| C | 1.29944 | -0.00214 | -0.1239 |
| C | 1.60386 | -1.33518 | -0.32331 |
| C | 3.02821 | -1.48052 | -0.38204 |
| C | 3.60367 | -0.23763 | -0.22041 |
| C | -0.82057 | -0.97912 | -0.22563 |
| C | -2.51183 | 0.56237 | 0.01727 |
| C | -2.2005 | -0.77342 | -0.17017 |
| C | -3.44296 | -1.62468 | -0.25266 |
| C | -4.50385 | -0.56197 | -0.08491 |
| C | -6.37331 | 0.82387 | 0.14872 |
| C | -5.89332 | -0.4918 | -0.03658 |
| C | 5.00578 | -0.31573 | -0.2405 |
| C | 5.47773 | -1.61158 | -0.42637 |
| C | 6.88277 | -1.72149 | -0.42413 |
| C | 7.49618 | -0.50974 | -0.23887 |
| S | 6.3261 | 0.79899 | -0.07538 |
| H | 7.42933 | -2.65191 | -0.52022 |
| C | -0.08418 | 0.20518 | -0.0732 |
| C | -3.92878 | 0.69416 | 0.06798 |
| C | -8.38688 | -0.29491 | 0.06176 |
| C | -9.7299 | -0.69487 | 0.07788 |
| H | -9.83523 | -1.77245 | -0.01559 |
| C | 8.92367 | -0.232 | -0.16865 |
| C | 9.42946 | 0.87759 | 0.51675 |
| C | 9.8453 | -1.09169 | -0.77975 |
| C | 10.78809 | 1.11234 | 0.60407 |
| H | 8.74861 | 1.55468 | 1.0259 |
| C | 11.20333 | -0.86155 | -0.69867 |
| H | 9.48713 | -1.94075 | -1.35449 |
| C | 11.69991 | 0.24279 | 0.00094 |
| H | 11.15433 | 1.96958 | 1.16046 |
| H | 11.89772 | -1.53637 | -1.19018 |
| N | 13.08462 | 0.46491 | 0.09546 |
| C | 13.96664 | -0.6283 | 0.27343 |
| C | 15.16383 | -0.6895 | -0.43914 |
| C | 13.65289 | -1.65047 | 1.16928 |
| C | 16.03492 | -1.75058 | -0.24821 |
| H | 15.40565 | 0.10244 | -1.142 |
| C | 14.52112 | -2.71678 | 1.34162 |
| H | 12.7229 | -1.60126 | 1.72829 |
| C | 15.71818 | -2.77115 | 0.63919 |
| H | 16.96417 | -1.78545 | -0.80863 |
| H | 14.26576 | -3.50519 | 2.04311 |
| H | 16.39968 | -3.60338 | 0.7822 |
| C | 13.60305 | 1.78131 | 0.07609 |
| C | 13.13745 | 2.71124 | -0.85377 |
| C | 14.59417 | 2.16067 | 0.98134 |
| C | 13.65073 | 3.99846 | -0.86856 |
| H | 12.3719 | 2.41593 | -1.56557 |
| C | 15.11485 | 3.44469 | 0.9482 |
| H | 14.9537 | 1.43945 | 1.70941 |
| C | 14.64442 | 4.37189 | 0.02748 |
| H | 13.2802 | 4.71197 | -1.5983 |
| H | 15.88666 | 3.72592 | 1.65816 |
| H | 15.04974 | 5.37833 | 0.00761 |
| S | 4.17634 | -2.77049 | -0.57171 |
| S | 0.21543 | -2.37126 | -0.44291 |
| S | -1.1176 | 1.5989 | 0.13446 |
| S | -5.06422 | 1.98766 | 0.26862 |
| S | -7.19715 | -1.60945 | -0.14078 |
| C | -7.74808 | 0.94075 | 0.20443 |
| H | -8.3141 | 1.8535 | 0.33484 |
| C | -3.47566 | -2.64353 | 0.88679 |
| H | -2.62699 | -3.33207 | 0.80542 |
| H | -3.42083 | -2.14359 | 1.85886 |
| H | -4.39541 | -3.23738 | 0.85247 |
| C | 2.59944 | 1.8691 | -1.16033 |
| H | 2.52969 | 1.38483 | -2.13946 |
| H | 1.76415 | 2.57234 | -1.06362 |
| H | 3.53079 | 2.44495 | -1.12341 |
| C | 2.70102 | 1.49719 | 1.33237 |
| H | 2.63009 | 0.75471 | 2.13349 |
| H | 3.67483 | 1.99432 | 1.40968 |
| H | 1.92298 | 2.25256 | 1.48836 |
| C | -3.54083 | -2.31345 | -1.61404 |
| H | -4.46671 | -2.89389 | -1.69158 |
| H | -3.523 | -1.57843 | -2.42472 |
| H | -2.69943 | -3.00148 | -1.75349 |
| C | -10.92512 | -0.00604 | 0.1661 |
| C | -11.11758 | 1.45348 | 0.1349 |
| C | -12.23553 | -0.62613 | 0.19683 |
| C | -13.24053 | 0.44898 | 0.06942 |
| C | -12.5758 | 1.67478 | 0.05353 |
| C | -13.23324 | 2.88463 | -0.03427 |
| C | -14.62861 | 0.42395 | -0.01477 |
| C | -14.6092 | 2.84907 | -0.11663 |
| H | -12.69896 | 3.82888 | -0.04045 |
| C | -15.28905 | 1.63283 | -0.11068 |
| H | -15.22364 | -0.4805 | -0.01669 |
| C | -12.55132 | -1.95565 | 0.37504 |
| O | -10.28757 | 2.34094 | 0.1509 |
| C | -11.59976 | -2.98152 | 0.6212 |
| N | -10.86007 | -3.84453 | 0.83353 |
| C | -13.88601 | -2.44143 | 0.37429 |
| N | -14.95675 | -2.8772 | 0.37714 |
| F | -16.61253 | 1.65338 | -0.20026 |
| F | -15.3141 | 3.96855 | -0.2058 |

**Table S2**: Cartesian coordinates of **FICD1** compound.

| **Atom** | **X-axis** | **Y-axis** | **Z-axis** |
| --- | --- | --- | --- |
| C | 3.72403 | 0.51864 | 0.13179 |
| C | 2.52359 | -0.38773 | -0.00911 |
| C | 2.91155 | -1.69806 | -0.20917 |
| C | 4.34464 | -1.7599 | -0.21624 |
| C | 4.83875 | -0.48829 | -0.01979 |
| C | 0.46762 | -1.49116 | -0.14092 |
| C | -1.31569 | -0.06168 | 0.12175 |
| C | -0.9244 | -1.37011 | -0.09568 |
| C | -2.11479 | -2.28806 | -0.22318 |
| C | -3.2381 | -1.29436 | -0.03953 |
| C | -5.18859 | -0.02454 | 0.18464 |
| C | -4.63254 | -1.30325 | -0.02526 |
| C | 6.24312 | -0.47532 | -0.0336 |
| C | 6.7993 | -1.73611 | -0.22787 |
| C | 8.20896 | -1.74732 | -0.25842 |
| C | 8.73962 | -0.49595 | -0.08731 |
| S | 7.48603 | 0.7251 | 0.12648 |
| H | 8.81768 | -2.62745 | -0.42772 |
| C | 1.1287 | -0.26741 | 0.03605 |
| C | -2.74047 | -0.01395 | 0.15742 |
| C | -7.1357 | -1.24234 | -0.00525 |
| C | -8.46177 | -1.70487 | -0.08538 |
| C | -9.68685 | -1.08243 | -0.01715 |
| H | -8.51027 | -2.77932 | -0.24436 |
| C | -11.99357 | -0.73483 | -0.10092 |
| C | -11.43383 | 0.496 | 0.07286 |
| C | 10.14605 | -0.11706 | -0.05357 |
| C | 10.57965 | 1.15379 | -0.44476 |
| C | 11.11643 | -1.03291 | 0.37005 |
| C | 11.91725 | 1.49831 | -0.41044 |
| H | 9.85679 | 1.88831 | -0.78976 |
| C | 12.45664 | -0.7014 | 0.3867 |
| H | 10.81139 | -2.02231 | 0.69847 |
| C | 12.87996 | 0.57387 | 0.00149 |
| H | 12.22798 | 2.49418 | -0.71132 |
| H | 13.19032 | -1.43331 | 0.71036 |
| C | -10.96477 | -1.76806 | -0.14939 |
| C | -9.97204 | 0.36568 | 0.14601 |
| N | 14.24318 | 0.92014 | 0.02806 |
| C | 15.0766 | 0.47391 | 1.07998 |
| C | 16.37069 | 0.02846 | 0.8097 |
| C | 14.62134 | 0.4843 | 2.39874 |
| C | 17.19323 | -0.39184 | 1.84298 |
| H | 16.72513 | 0.01718 | -0.21696 |
| C | 15.4447 | 0.04697 | 3.42422 |
| H | 13.61687 | 0.8384 | 2.6118 |
| C | 16.73525 | -0.38958 | 3.15433 |
| H | 18.1984 | -0.73558 | 1.61843 |
| H | 15.07773 | 0.06186 | 4.44598 |
| H | 17.38009 | -0.72419 | 3.96043 |
| C | 14.78326 | 1.75113 | -0.98328 |
| C | 14.49054 | 1.51001 | -2.32544 |
| C | 15.61791 | 2.81681 | -0.64806 |
| C | 15.01727 | 2.3294 | -3.31136 |
| H | 13.84744 | 0.67517 | -2.58849 |
| C | 16.15373 | 3.62204 | -1.64098 |
| H | 15.8436 | 3.00755 | 0.39717 |
| C | 15.85386 | 3.38646 | -2.97649 |
| H | 14.78223 | 2.13026 | -4.35253 |
| H | 16.80141 | 4.44903 | -1.36629 |
| H | 16.27001 | 4.02187 | -3.75154 |
| O | -9.19648 | 1.28754 | 0.30453 |
| C | -11.25456 | -3.10668 | -0.28507 |
| C | -10.29327 | -4.15046 | -0.30099 |
| N | -9.53751 | -5.02505 | -0.31467 |
| C | -12.59782 | -3.54788 | -0.41396 |
| N | -13.68905 | -3.91412 | -0.52166 |
| C | -13.68165 | 1.0545 | -0.00476 |
| C | -12.37056 | 1.55616 | 0.13473 |
| C | -12.16805 | 2.92832 | 0.30131 |
| C | -13.259 | 3.76752 | 0.32493 |
| C | -14.56347 | 3.25438 | 0.18489 |
| C | -14.78187 | 1.90023 | 0.01956 |
| H | -11.16419 | 3.3235 | 0.40956 |
| H | -15.79424 | 1.52685 | -0.08687 |
| S | -13.72307 | -0.69126 | -0.20361 |
| Cl | -12.99881 | 5.47217 | 0.52961 |
| Cl | -15.93775 | 4.31145 | 0.21502 |
| S | 5.57451 | -2.97117 | -0.41299 |
| S | 1.58971 | -2.81496 | -0.35827 |
| S | 0.01065 | 1.05514 | 0.26911 |
| S | -3.95167 | 1.20639 | 0.36904 |
| S | -5.87172 | -2.48468 | -0.20657 |
| C | -6.57126 | 0.01589 | 0.19747 |
| H | -7.18703 | 0.89278 | 0.33999 |
| C | -2.10401 | -3.34796 | 0.87837 |
| H | -1.21569 | -3.98291 | 0.78429 |
| H | -2.09046 | -2.8821 | 1.86866 |
| H | -2.98791 | -3.99144 | 0.80875 |
| C | 3.78786 | 1.5627 | -0.98314 |
| H | 3.73152 | 1.08605 | -1.96687 |
| H | 2.96595 | 2.28213 | -0.89772 |
| H | 4.73043 | 2.11917 | -0.9246 |
| C | 3.74263 | 1.18127 | 1.50923 |
| H | 3.73319 | 0.42805 | 2.3033 |
| H | 4.63605 | 1.80344 | 1.63171 |
| H | 2.86349 | 1.824 | 1.63372 |
| C | -2.15407 | -2.93381 | -1.60854 |
| H | -3.04275 | -3.56479 | -1.71876 |
| H | -2.16989 | -2.17156 | -2.3937 |
| H | -1.27114 | -3.56524 | -1.75845 |

**Table S3**: Cartesian coordinates of **FICD2** compound.

| **Atom** | **X-axis** | **Y-axis** | **Z-axis** |
| --- | --- | --- | --- |
| C | 4.02832 | 0.39951 | 0.27823 |
| C | 2.84381 | -0.52953 | 0.156 |
| C | 3.25336 | -1.83546 | -0.03307 |
| C | 4.68709 | -1.87217 | -0.04983 |
| C | 5.15859 | -0.58899 | 0.12714 |
| C | 0.80443 | -1.66671 | 0.02626 |
| C | -0.99606 | -0.25218 | 0.24735 |
| C | -0.58942 | -1.56231 | 0.05408 |
| C | -1.77401 | -2.48525 | -0.10864 |
| C | -2.90429 | -1.49191 | 0.02077 |
| C | -4.86551 | -0.23178 | 0.19158 |
| C | -4.29537 | -1.5104 | 0.00078 |
| C | 6.56131 | -0.54761 | 0.09382 |
| C | 7.14132 | -1.79901 | -0.09328 |
| C | 8.5505 | -1.7787 | -0.15115 |
| C | 9.05451 | -0.51261 | -0.01123 |
| S | 7.77857 | 0.6827 | 0.21205 |
| H | 9.17665 | -2.64649 | -0.32108 |
| C | 1.44839 | -0.43107 | 0.19517 |
| C | -2.41842 | -0.20766 | 0.2303 |
| C | -6.79844 | -1.46677 | -0.03736 |
| C | -8.11539 | -1.93309 | -0.14656 |
| C | -9.34877 | -1.31337 | -0.10129 |
| H | -8.15797 | -3.0073 | -0.30944 |
| C | -11.65636 | -0.97445 | -0.21533 |
| C | -11.10352 | 0.2544 | -0.01818 |
| C | 10.4503 | -0.09398 | -0.02341 |
| C | 10.8338 | 1.17045 | -0.48172 |
| C | 11.45754 | -0.95937 | 0.41899 |
| C | 12.16007 | 1.55773 | -0.49429 |
| H | 10.08039 | 1.86491 | -0.84372 |
| C | 12.78657 | -0.58416 | 0.39135 |
| H | 11.19118 | -1.9416 | 0.79839 |
| C | 13.15944 | 0.68397 | -0.06159 |
| H | 12.43318 | 2.54704 | -0.84871 |
| H | 13.55248 | -1.27376 | 0.7334 |
| C | -10.61618 | -2.00354 | -0.26226 |
| C | -9.63975 | 0.1292 | 0.07304 |
| N | 14.5119 | 1.07379 | -0.08215 |
| C | 15.37125 | 0.73589 | 0.98818 |
| C | 16.6861 | 0.34039 | 0.73858 |
| C | 14.92106 | 0.80385 | 2.30734 |
| C | 17.53162 | 0.02602 | 1.7907 |
| H | 17.03945 | 0.28339 | -0.28684 |
| C | 15.76797 | 0.47044 | 3.35229 |
| H | 13.90237 | 1.12256 | 2.50761 |
| C | 17.0783 | 0.08379 | 3.10236 |
| H | 18.5522 | -0.27954 | 1.58087 |
| H | 15.4029 | 0.52844 | 4.37323 |
| H | 17.74142 | -0.16924 | 3.92317 |
| C | 15.00372 | 1.84553 | -1.16264 |
| C | 14.69369 | 1.49098 | -2.47549 |
| C | 15.79951 | 2.96656 | -0.92779 |
| C | 15.16214 | 2.25399 | -3.53322 |
| H | 14.08155 | 0.61244 | -2.65795 |
| C | 16.27754 | 3.71541 | -1.99169 |
| H | 16.03874 | 3.24534 | 0.09439 |
| C | 15.95866 | 3.36756 | -3.29822 |
| H | 14.91348 | 1.9675 | -4.55052 |
| H | 16.89494 | 4.58687 | -1.79628 |
| H | 16.32988 | 3.95993 | -4.12823 |
| O | -8.87643 | 1.05679 | 0.25235 |
| C | -10.90393 | -3.3402 | -0.42116 |
| C | -9.94083 | -4.38207 | -0.44072 |
| N | -9.18174 | -5.25355 | -0.45799 |
| C | -12.24596 | -3.77689 | -0.57247 |
| N | -13.33925 | -4.13146 | -0.69751 |
| C | -13.35279 | 0.79952 | -0.12408 |
| C | -12.04576 | 1.30497 | 0.04744 |
| C | -11.84894 | 2.67836 | 0.23088 |
| C | -12.94553 | 3.49975 | 0.24961 |
| C | -14.24416 | 2.98315 | 0.10197 |
| C | -14.46083 | 1.63884 | -0.09113 |
| H | -10.85009 | 3.08852 | 0.32977 |
| H | -15.47601 | 1.26999 | -0.18416 |
| S | -13.38251 | -0.93816 | -0.34332 |
| S | 5.93924 | -3.06089 | -0.24786 |
| S | 1.95052 | -2.97374 | -0.17691 |
| S | 0.31464 | 0.88106 | 0.40005 |
| S | -3.64032 | 1.00762 | 0.40445 |
| S | -5.51988 | -2.7022 | -0.2043 |
| C | -6.24472 | -0.20074 | 0.17179 |
| H | -6.86903 | 0.67258 | 0.297 |
| C | -1.84229 | -3.53041 | 1.00521 |
| H | -1.0068 | -4.23525 | 0.93484 |
| H | -1.81347 | -3.05416 | 1.99018 |
| H | -2.77308 | -4.10371 | 0.92724 |
| C | 4.06493 | 1.43222 | -0.84867 |
| H | 4.00449 | 0.94429 | -1.82662 |
| H | 3.23478 | 2.14189 | -0.76203 |
| H | 5.00022 | 2.00223 | -0.80672 |
| C | 4.04845 | 1.07714 | 1.64835 |
| H | 4.05911 | 0.33252 | 2.45044 |
| H | 4.93258 | 1.71516 | 1.75549 |
| H | 3.16022 | 1.70678 | 1.77442 |
| C | -1.75219 | -3.14677 | -1.48683 |
| H | -2.63239 | -3.78311 | -1.62843 |
| H | -1.73677 | -2.3936 | -2.28069 |
| H | -0.86061 | -3.77535 | -1.59085 |
| N | -12.68984 | 4.9508 | 0.28252 |
| O | -13.32527 | 5.63611 | -0.4859 |
| O | -11.82728 | 5.34027 | 1.0357 |
| N | -15.43438 | 3.82467 | 0.30237 |
| O | -16.42148 | 3.55155 | -0.34294 |
| O | -15.35283 | 4.69978 | 1.13542 |

**Table S4**: Cartesian coordinates of **FICD3** compound.

| **Atom** | **X-axis** | **Y-axis** | **Z-axis** |
| --- | --- | --- | --- |
| C | 4.50914 | 0.25066 | 0.42244 |
| C | 3.34472 | -0.69027 | 0.22655 |
| C | 3.77901 | -1.96015 | -0.09914 |
| C | 5.21301 | -1.95829 | -0.14823 |
| C | 5.65896 | -0.68809 | 0.14894 |
| C | 1.32803 | -1.85239 | 0.01005 |
| C | -0.49923 | -0.50792 | 0.39043 |
| C | -0.06726 | -1.78093 | 0.06342 |
| C | -1.2306 | -2.71715 | -0.15746 |
| C | -2.38395 | -1.77194 | 0.08982 |
| C | -4.36931 | -0.57102 | 0.38289 |
| C | -3.77696 | -1.81509 | 0.0748 |
| C | 7.05867 | -0.60674 | 0.08301 |
| C | 7.66302 | -1.81434 | -0.25439 |
| C | 9.06859 | -1.74844 | -0.35185 |
| C | 9.54671 | -0.49126 | -0.08941 |
| S | 8.24836 | 0.63622 | 0.29888 |
| H | 9.70839 | -2.57672 | -0.63218 |
| C | 1.94819 | -0.62646 | 0.29349 |
| C | -1.92364 | -0.50116 | 0.40783 |
| C | -6.28141 | -1.80841 | 0.03099 |
| C | -7.59254 | -2.28709 | -0.12508 |
| C | -8.83242 | -1.68881 | -0.05953 |
| H | -7.6156 | -3.34776 | -0.36365 |
| C | -11.13657 | -1.3637 | -0.25959 |
| C | -10.60244 | -0.13594 | -0.01029 |
| C | 10.92883 | -0.03073 | -0.09435 |
| C | 11.25319 | 1.31168 | -0.31665 |
| C | 11.98382 | -0.92578 | 0.12072 |
| C | 12.56461 | 1.7452 | -0.31532 |
| H | 10.46368 | 2.03587 | -0.49842 |
| C | 13.29809 | -0.50261 | 0.09971 |
| H | 11.76865 | -1.97273 | 0.3135 |
| C | 13.61106 | 0.84321 | -0.11211 |
| H | 12.78877 | 2.79445 | -0.48124 |
| H | 14.09939 | -1.21757 | 0.25958 |
| C | -10.09048 | -2.38494 | -0.27788 |
| C | -9.14521 | -0.2536 | 0.14911 |
| N | 14.94848 | 1.27957 | -0.11957 |
| C | 15.88214 | 0.74925 | 0.8008 |
| C | 17.18497 | 0.46126 | 0.39235 |
| C | 15.52017 | 0.51954 | 2.1287 |
| C | 18.1062 | -0.03978 | 1.29839 |
| H | 17.46952 | 0.63633 | -0.64121 |
| C | 16.44283 | 0.00172 | 3.02392 |
| H | 14.51065 | 0.7523 | 2.45471 |
| C | 17.74129 | -0.27766 | 2.61735 |
| H | 19.11631 | -0.25844 | 0.96545 |
| H | 16.14628 | -0.17113 | 4.05408 |
| H | 18.46288 | -0.67662 | 3.32272 |
| C | 15.35569 | 2.29125 | -1.02294 |
| C | 14.97266 | 2.23266 | -2.36287 |
| C | 16.14435 | 3.35472 | -0.58503 |
| C | 15.36411 | 3.22827 | -3.24361 |
| H | 14.36511 | 1.40037 | -2.70649 |
| C | 16.54618 | 4.33771 | -1.47589 |
| H | 16.44041 | 3.40356 | 0.45878 |
| C | 16.15517 | 4.28372 | -2.80759 |
| H | 15.05867 | 3.17118 | -4.28393 |
| H | 17.15972 | 5.16051 | -1.12175 |
| H | 16.46573 | 5.05805 | -3.50155 |
| O | -8.39614 | 0.67063 | 0.39461 |
| C | -10.35769 | -3.72335 | -0.45197 |
| C | -9.38513 | -4.75628 | -0.41685 |
| N | -8.61945 | -5.62171 | -0.38973 |
| C | -11.68611 | -4.17472 | -0.66878 |
| N | -12.76598 | -4.54617 | -0.8485 |
| C | -12.8457 | 0.40319 | -0.20982 |
| C | -11.55567 | 0.90954 | 0.02709 |
| C | -11.38635 | 2.27825 | 0.24714 |
| C | -12.47604 | 3.12205 | 0.22649 |
| C | -13.77041 | 2.60216 | -0.03109 |
| C | -13.94872 | 1.24683 | -0.23816 |
| H | -10.39047 | 2.66108 | 0.4346 |
| H | -14.94048 | 0.85466 | -0.42772 |
| S | -12.85711 | -1.33323 | -0.46323 |
| S | 6.48668 | -3.08524 | -0.50515 |
| S | 2.49647 | -3.10713 | -0.33945 |
| S | 0.78967 | 0.63361 | 0.6402 |
| S | -3.16763 | 0.66707 | 0.70222 |
| S | -4.98333 | -3.00223 | -0.23996 |
| C | -5.75086 | -0.56095 | 0.36181 |
| H | -6.38867 | 0.28779 | 0.56389 |
| C | -1.21121 | -3.86293 | 0.8548 |
| H | -0.31388 | -4.4768 | 0.71799 |
| H | -1.21411 | -3.47942 | 1.8798 |
| H | -2.08486 | -4.51074 | 0.72365 |
| C | 4.49165 | 1.39463 | -0.59155 |
| H | 4.4354 | 1.00845 | -1.61417 |
| H | 3.63458 | 2.05573 | -0.42168 |
| H | 5.40393 | 1.99509 | -0.49987 |
| C | 4.54259 | 0.78613 | 1.85392 |
| H | 4.58407 | -0.03473 | 2.57667 |
| H | 5.41555 | 1.42967 | 2.00922 |
| H | 3.64521 | 1.38128 | 2.05884 |
| C | -1.23248 | -3.25005 | -1.59029 |
| H | -2.10096 | -3.89476 | -1.7641 |
| H | -1.25913 | -2.42876 | -2.31315 |
| H | -0.33008 | -3.84362 | -1.77543 |
| C | -12.19779 | 4.5905 | 0.42816 |
| C | -15.01564 | 3.4532 | -0.04301 |
| F | -10.99094 | 4.78672 | 0.96437 |
| F | -13.07835 | 5.17291 | 1.2433 |
| F | -12.21375 | 5.25841 | -0.72847 |
| F | -15.42185 | 3.7511 | 1.19387 |
| F | -14.844 | 4.60048 | -0.69969 |
| F | -16.03127 | 2.81938 | -0.63579 |

**Table S5**: Cartesian coordinates of **FICD4** compound.

| **Atom** | **X-axis** | **Y-axis** | **Z-axis** |
| --- | --- | --- | --- |
| C | 4.41958 | 0.29339 | 0.3432 |
| C | 3.2534 | -0.64708 | 0.1532 |
| C | 3.68701 | -1.92425 | -0.14294 |
| C | 5.12151 | -1.92769 | -0.17925 |
| C | 5.56894 | -0.6537 | 0.09764 |
| C | 1.23652 | -1.81098 | -0.04806 |
| C | -0.5934 | -0.46065 | 0.29855 |
| C | -0.1591 | -1.73898 | -0.00035 |
| C | -1.31931 | -2.683 | -0.1993 |
| C | -2.47479 | -1.73713 | 0.03258 |
| C | -4.4661 | -0.54154 | 0.30785 |
| C | -3.86844 | -1.78782 | 0.02597 |
| C | 6.96981 | -0.57869 | 0.04454 |
| C | 7.5728 | -1.79489 | -0.26276 |
| C | 8.97972 | -1.73727 | -0.34355 |
| C | 9.4608 | -0.47794 | -0.09773 |
| S | 8.16281 | 0.6627 | 0.25231 |
| H | 9.61893 | -2.57374 | -0.59987 |
| C | 1.85589 | -0.58003 | 0.21187 |
| C | -2.01927 | -0.45827 | 0.31931 |
| C | -6.37209 | -1.80017 | 0.00293 |
| C | -7.68148 | -2.29845 | -0.12317 |
| C | -8.92656 | -1.71737 | -0.0522 |
| H | -7.69263 | -3.36554 | -0.3315 |
| C | -11.24059 | -1.4375 | -0.18102 |
| C | -10.72235 | -0.1933 | 0.01945 |
| C | 10.84572 | -0.02606 | -0.09088 |
| C | 11.18274 | 1.31082 | -0.32686 |
| C | 11.89146 | -0.92522 | 0.15066 |
| C | 12.49716 | 1.73522 | -0.31293 |
| H | 10.40104 | 2.03809 | -0.52906 |
| C | 13.20888 | -0.51165 | 0.14194 |
| H | 11.66628 | -1.96792 | 0.35471 |
| C | 13.53443 | 0.82897 | -0.08327 |
| H | 12.73088 | 2.78063 | -0.48962 |
| H | 14.00255 | -1.23029 | 0.32212 |
| C | -10.17982 | -2.43888 | -0.22233 |
| C | -9.25895 | -0.28202 | 0.13178 |
| N | 14.87505 | 1.25575 | -0.07672 |
| C | 15.79106 | 0.72939 | 0.86357 |
| C | 17.09634 | 0.42306 | 0.47711 |
| C | 15.40868 | 0.52147 | 2.18934 |
| C | 18.00006 | -0.07418 | 1.40258 |
| H | 17.39654 | 0.5804 | -0.55487 |
| C | 16.31375 | 0.00696 | 3.10431 |
| H | 14.39699 | 0.76824 | 2.49786 |
| C | 17.61484 | -0.29027 | 2.71954 |
| H | 19.01243 | -0.30723 | 1.08671 |
| H | 16.00139 | -0.14885 | 4.13251 |
| H | 18.3229 | -0.6864 | 3.44007 |
| C | 15.3044 | 2.2508 | -0.98801 |
| C | 14.93595 | 2.18068 | -2.33154 |
| C | 16.10207 | 3.30934 | -0.55432 |
| C | 15.35166 | 3.15948 | -3.22011 |
| H | 14.32122 | 1.35246 | -2.67212 |
| C | 16.5281 | 4.27506 | -1.4527 |
| H | 16.3868 | 3.36747 | 0.49217 |
| C | 16.15226 | 4.20937 | -2.78824 |
| H | 15.0576 | 3.09312 | -4.26318 |
| H | 17.14881 | 5.09382 | -1.10164 |
| H | 16.48198 | 4.97019 | -3.48827 |
| O | -8.51569 | 0.65812 | 0.33088 |
| C | -10.42548 | -3.78379 | -0.37762 |
| C | -9.43278 | -4.79802 | -0.37727 |
| N | -8.65199 | -5.65037 | -0.37848 |
| C | -11.75094 | -4.26523 | -0.54287 |
| N | -12.82656 | -4.66569 | -0.68071 |
| C | -12.98587 | 0.29658 | -0.08843 |
| C | -11.69314 | 0.83711 | 0.07558 |
| C | -11.54072 | 2.21213 | 0.27145 |
| C | -12.65796 | 3.02472 | 0.2861 |
| C | -13.94422 | 2.47007 | 0.09812 |
| C | -14.11361 | 1.10942 | -0.06904 |
| H | -10.5524 | 2.6293 | 0.42895 |
| H | -15.11025 | 0.69953 | -0.19807 |
| S | -12.96936 | -1.4443 | -0.30996 |
| S | 6.39415 | -3.06588 | -0.50195 |
| S | 2.40409 | -3.07463 | -0.364 |
| S | 0.69471 | 0.68649 | 0.52723 |
| S | -3.26916 | 0.70992 | 0.59137 |
| S | -5.06848 | -2.99038 | -0.25074 |
| C | -5.84961 | -0.54206 | 0.29717 |
| H | -6.49289 | 0.30593 | 0.48426 |
| C | -1.28943 | -3.80934 | 0.8341 |
| H | -0.38541 | -4.41578 | 0.70777 |
| H | -1.29502 | -3.4066 | 1.85173 |
| H | -2.15656 | -4.46838 | 0.7162 |
| C | 4.41836 | 1.42091 | -0.68907 |
| H | 4.36748 | 1.01882 | -1.70583 |
| H | 3.56565 | 2.09192 | -0.5372 |
| H | 5.3348 | 2.01546 | -0.59948 |
| C | 4.44056 | 0.85186 | 1.76623 |
| H | 4.47177 | 0.04247 | 2.5023 |
| H | 5.31441 | 1.49457 | 1.92 |
| H | 3.54322 | 1.45319 | 1.95243 |
| C | -1.3243 | -3.24289 | -1.62195 |
| H | -2.19154 | -3.89311 | -1.78086 |
| H | -1.3558 | -2.43518 | -2.35977 |
| H | -0.42068 | -3.83707 | -1.79901 |
| C | -15.17714 | 3.31335 | 0.04873 |
| O | -16.12235 | 3.16387 | 0.775 |
| O | -15.11716 | 4.18668 | -0.9537 |
| C | -12.53044 | 4.46475 | 0.63331 |
| O | -13.42418 | 5.13196 | 1.09065 |
| O | -11.30012 | 4.92514 | 0.415 |
| C | -16.18256 | 5.13572 | -1.00316 |
| H | -17.1488 | 4.6319 | -1.07275 |
| H | -16.1603 | 5.75854 | -0.10463 |
| H | -16.00472 | 5.74299 | -1.88817 |
| C | -11.07558 | 6.28706 | 0.78214 |
| H | -11.73604 | 6.94737 | 0.21596 |
| H | -11.25574 | 6.43009 | 1.84988 |
| H | -10.03507 | 6.49447 | 0.54165 |

**Table S6**: Cartesian coordinates of **FICD5** compound.

| **Atom** | **X-axis** | **Y-axis** | **Z-axis** |
| --- | --- | --- | --- |
| C | 3.50746 | 0.56213 | 0.28047 |
| C | 2.30147 | -0.32796 | 0.08803 |
| C | 2.68294 | -1.62585 | -0.19455 |
| C | 4.11426 | -1.69321 | -0.21443 |
| C | 4.61667 | -0.43885 | 0.06121 |
| C | 0.24134 | -1.4132 | -0.11135 |
| C | -1.53612 | 0.00898 | 0.22236 |
| C | -1.14865 | -1.28856 | -0.065 |
| C | -2.34097 | -2.19394 | -0.2486 |
| C | -3.46152 | -1.20594 | -0.02321 |
| C | -5.40677 | 0.06344 | 0.24748 |
| C | -4.85373 | -1.2082 | -0.02255 |
| C | 6.02079 | -0.43236 | 0.03507 |
| C | 6.5669 | -1.67946 | -0.25434 |
| C | 7.97483 | -1.69683 | -0.30455 |
| C | 8.51839 | -0.46487 | -0.04813 |
| S | 7.27486 | 0.74491 | 0.26867 |
| H | 8.57098 | -2.56852 | -0.54548 |
| C | 0.90793 | -0.20423 | 0.13915 |
| C | -2.95877 | 0.06149 | 0.24705 |
| C | -7.35711 | -1.13441 | -0.02616 |
| C | -8.68106 | -1.58359 | -0.13194 |
| C | -9.90637 | -0.9537 | -0.04729 |
| H | -8.73769 | -2.65081 | -0.33256 |
| C | -12.20982 | -0.58539 | -0.13853 |
| C | -11.6409 | 0.63573 | 0.06024 |
| C | 9.92776 | -0.10013 | -0.00931 |
| C | 10.35831 | 1.21964 | -0.18331 |
| C | 10.90928 | -1.07725 | 0.19626 |
| C | 11.69918 | 1.54948 | -0.15309 |
| H | 9.6295 | 2.00843 | -0.35046 |
| C | 12.25224 | -0.75866 | 0.20365 |
| H | 10.61558 | -2.11039 | 0.35638 |
| C | 12.67266 | 0.56383 | 0.03014 |
| H | 12.00513 | 2.58305 | -0.28311 |
| H | 12.99076 | -1.54058 | 0.3503 |
| C | -11.18399 | -1.62725 | -0.19847 |
| C | -10.17835 | 0.49096 | 0.14237 |
| N | 14.03654 | 0.89861 | 0.03309 |
| C | 14.95553 | 0.19515 | 0.84766 |
| C | 16.18335 | -0.21241 | 0.32588 |
| C | 14.65368 | -0.08708 | 2.18015 |
| C | 17.09317 | -0.88463 | 1.12644 |
| H | 16.4189 | 0.00558 | -0.7119 |
| C | 15.56236 | -0.77442 | 2.96928 |
| H | 13.70277 | 0.23814 | 2.59219 |
| C | 16.78732 | -1.17378 | 2.44992 |
| H | 18.04535 | -1.19552 | 0.70746 |
| H | 15.3154 | -0.98688 | 4.00505 |
| H | 17.4992 | -1.70496 | 3.07333 |
| C | 14.52262 | 1.90711 | -0.83621 |
| C | 14.14151 | 1.93025 | -2.17722 |
| C | 15.40255 | 2.87677 | -0.35789 |
| C | 14.62765 | 2.91589 | -3.02221 |
| H | 13.46242 | 1.16892 | -2.55036 |
| C | 15.89683 | 3.84971 | -1.21251 |
| H | 15.6965 | 2.85914 | 0.68757 |
| C | 15.51002 | 3.87716 | -2.54619 |
| H | 14.32511 | 2.92318 | -4.06481 |
| H | 16.58187 | 4.59987 | -0.82938 |
| H | 15.89482 | 4.64334 | -3.21148 |
| O | -9.39997 | 1.40557 | 0.32379 |
| C | -11.48858 | -2.9606 | -0.35316 |
| C | -10.53729 | -4.01334 | -0.37279 |
| N | -9.7873 | -4.8927 | -0.38915 |
| C | -12.83639 | -3.38218 | -0.49643 |
| N | -13.93414 | -3.72485 | -0.61528 |
| C | -13.886 | 1.20914 | -0.0216 |
| C | -12.56971 | 1.69974 | 0.13452 |
| C | -12.35507 | 3.0665 | 0.32584 |
| C | -13.44263 | 3.91786 | 0.35793 |
| C | -14.76121 | 3.41342 | 0.19953 |
| C | -14.98378 | 2.05847 | 0.00973 |
| H | -11.34739 | 3.44823 | 0.44589 |
| H | -15.99531 | 1.68682 | -0.1101 |
| S | -13.93721 | -0.52822 | -0.25 |
| S | 5.33471 | -2.89365 | -0.50799 |
| S | 1.35688 | -2.72618 | -0.41136 |
| S | -0.20442 | 1.10875 | 0.44208 |
| S | -4.16517 | 1.27623 | 0.50928 |
| S | -6.0958 | -2.37259 | -0.276 |
| C | -6.78627 | 0.11152 | 0.24665 |
| H | -7.39837 | 0.98436 | 0.4263 |
| C | -2.34196 | -3.30764 | 0.79891 |
| H | -1.4568 | -3.94206 | 0.67671 |
| H | -2.33091 | -2.89224 | 1.81139 |
| H | -3.22907 | -3.94131 | 0.69242 |
| C | 3.5706 | 1.67242 | -0.7686 |
| H | 3.51167 | 1.25711 | -1.77959 |
| H | 2.74953 | 2.38586 | -0.63711 |
| H | 4.51353 | 2.22369 | -0.67746 |
| C | 3.53684 | 1.14002 | 1.69527 |
| H | 3.52289 | 0.34116 | 2.44326 |
| H | 4.43716 | 1.74475 | 1.85025 |
| H | 2.66558 | 1.784 | 1.8618 |
| C | -2.37096 | -2.7707 | -1.6642 |
| H | -3.25975 | -3.39386 | -1.81148 |
| H | -2.37896 | -1.97159 | -2.41188 |
| H | -1.48827 | -3.39657 | -1.83727 |
| C | -13.23541 | 5.31576 | 0.55072 |
| N | -13.06574 | 6.44589 | 0.70619 |
| C | -15.87258 | 4.30504 | 0.23361 |
| N | -16.77233 | 5.02662 | 0.26068 |

**Table S7**: The representative NBOs values for **FICR.**

| **Compounds** | **Donor(*i*)** | **Type** | **Acceptor(*j*)** | **Type** | ***E* (2)**  **[*kcal/mol*]** | ***E*(*j*)-*E*(*i*)**  **[*a.u*]** |
| --- | --- | --- | --- | --- | --- | --- |
| C4-S57 | σ | C13-S17 | σ* | 0.51 | 0.9 | 0.02 |
| C22-C80 | σ | C21-S61 | σ* | 2.60 | 0.92 | 0.04 |
| C86-C89 | σ | C87-C89 | σ* | 3.99 | 1.27 | 0.06 |
| C1-C5 | σ | C4-C5 | σ* | 4.00 | 1.19 | 0.06 |
| C81-O92 | π | C84-C85 | π* | 4.20 | 0.43 | 0.04 |
| C15-C16 | σ | C14-C15 | σ* | 5.00 | 1.28 | 0.07 |
| C86-C89 | σ | C82-C83 | σ* | 5.55 | 1.22 | 0.07 |
| C21-S61 | σ | C10-C12 | σ* | 5.59 | 1.22 | 0.07 |
| C5-C13 | σ | C13-C14 | σ* | 5.60 | 1.26 | 0.08 |
| C9-C10 | σ | C10-C12 | σ* | 5.61 | 1.21 | 0.07 |
| C2-C3 | σ | C2-C19 | σ* | 5.62 | 1.28 | 0.08 |
| C6-C8 | σ | C8-C9 | σ* | 5.88 | 1.16 | 0.07 |
| C2-C19 | σ | C1-C2 | σ* | 5.96 | 1.16 | 0.07 |
| C82-C91 | σ | C91-C95 | σ* | 6.05 | 1.27 | 0.08 |
| C22-C80 | σ | C21-C22 | σ* | 6.45 | 1.31 | 0.08 |
| C13-C14 | σ | C5-C13 | σ* | 6.63 | 1.29 | 0.08 |
| C22-H23 | σ | C80-C81 | σ* | 6.98 | 0.99 | 0.08 |
| C22-H23 | σ | C21-C62 | σ* | 7.27 | 1.08 | 0.08 |
| C82-C91 | π | C22-C80 | π* | 7.79 | 0.33 | 0.05 |
| C7-C8 | σ | C20-S60 | σ* | 8.07 | 0.91 | 0.08 |
| C95-N96 | σ | C91-C95 | σ* | 8.09 | 1.57 | 0.10 |
| C10-C20 | σ | C12-S61 | σ* | 8.58 | 0.92 | 0.08 |
| C82-C91 | π | C83-C86 | π* | 9.40 | 0.32 | 0.05 |
| C15-C16 | π | C24-C25 | π* | 11.48 | 0.32 | 0.06 |
| C22-C80 | π | C21-C62 | π* | 12.22 | 0.29 | 0.06 |
| C10-C20 | π | C7-C8 | π* | 14.11 | 0.31 | 0.06 |
| C2-C3 | π | C4-C5 | π* | 14.44 | 0.31 | 0.06 |
| C7-C8 | π | C6-C19 | π* | 15.02 | 0.28 | 0.06 |
| C4-C5 | π | C13-C14 | π* | 15.50 | 0.29 | 0.06 |
| C24-C25 | π | C15-C16 | π* | 15.58 | 0.29 | 0.06 |
| C15-C16 | π | C13-C14 | π* | 15.67 | 0.29 | 0.07 |
| C4-C5 | π | C2-C3 | π* | 15.85 | 0.31 | 0.07 |
| C21-C62 | π | C11-C12 | π* | 15.87 | 0.27 | 0.06 |
| C13-C14 | π | C15-C16 | π* | 16.50 | 0.32 | 0.06 |
| C83-C86 | π | C82-C91 | π* | 17.27 | 0.30 | 0.06 |
| C84-C85 | π | C81-O92 | π* | 17.51 | 0.31 | 0.07 |
| C2-C3 | π | C6-C19 | π* | 17.58 | 0.28 | 0.07 |
| C11-C12 | π | C10-C20 | π* | 17.60 | 0.31 | 0.07 |
| C6-C19 | π | C2-C3 | π* | 18.26 | 0.31 | 0.07 |
| C13-C14 | π | C4-C5 | π* | 18.7 | 0.32 | 0.07 |
| C7-C8 | π | C10-C20 | π* | 18.76 | 0.30 | 0.07 |
| C26-C29 | π | C24-C25 | π* | 19.24 | 0.30 | 0.07 |
| C27-C31 | π | C26-C29 | π* | 19.48 | 0.30 | 0.07 |
| C46-C48 | π | C47-C49 | π* | 20.38 | 0.30 | 0.07 |
| C47-C49 | π | C51-C53 | π* | 20.41 | 0.30 | 0.07 |
| C35-C36 | π | C37-C40 | π* | 20.45 | 0.30 | 0.07 |
| C37-C40 | π | C38-C42 | π* | 20.5 | 0.30 | 0.07 |
| C87-C89 | π | C84-C85 | π* | 20.79 | 0.33 | 0.08 |
| C10-C20 | π | C11-C12 | π* | 20.86 | 0.27 | 0.07 |
| C87-C89 | π | C83-C86 | π* | 21.43 | 0.32 | 0.08 |
| C24-C25 | π | C26-C29 | π* | 21.62 | 0.30 | 0.07 |
| C26-C29 | π | C27-C31 | π* | 21.67 | 0.30 | 0.07 |
| C51-C53 | π | C46-C48 | π* | 21.77 | 0.29 | 0.07 |
| C38-C42 | π | C35-C36 | π* | 21.84 | 0.29 | 0.07 |
| C24-C25 | π | C27-C31 | π* | 21.89 | 0.29 | 0.07 |
| C6-C19 | π | C7-C8 | π* | 21.90 | 0.31 | 0.08 |
| C84-C85 | π | C83-C86 | π* | 22.12 | 0.30 | 0.07 |
| C83-C86 | π | C84-C85 | π* | 22.13 | 0.31 | 0.07 |
| C47-C49 | π | C46-C48 | π* | 23.03 | 0.29 | 0.08 |
| C37-C40 | π | C35-C36 | π* | 23.05 | 0.29 | 0.08 |
| C35-C36 | π | C38-C42 | π* | 23.06 | 0.30 | 0.08 |
| C46-C48 | π | C51-C53 | π* | 23.11 | 0.30 | 0.08 |
| C38-C42 | π | C37-C40 | π* | 23.56 | 0.30 | 0.08 |
| C51-C53 | π | C47-C49 | π* | 23.59 | 0.30 | 0.08 |
| C27-C31 | π | C24-C25 | π* | 24.05 | 0.30 | 0.08 |
| C22-C80 | π | C81-O92 | π* | 24.28 | 0.30 | 0.08 |
| C84-C85 | π | C87-C89 | π* | 24.47 | 0.27 | 0.07 |
| C83-C86 | π | C87-C89 | π* | 24.67 | 0.28 | 0.08 |
| C11-C12 | π | C21-C62 | π* | 25.97 | 0.30 | 0.08 |
| C22-C80 | π | C82-C91 | π* | 28.04 | 0.29 | 0.08 |
| C21-C62 | π | C22-C80 | π* | 31.18 | 0.30 | 0.09 |
| S57 | LP(1) | C3-C4 | σ* | 0.92 | 1.18 | 0.03 |
| O92 | LP(2) | C62-H63 | σ* | 2.27 | 0.71 | 0.04 |
| N34 | LP(1) | C35-C37 | σ* | 3.48 | 0.85 | 0.05 |
| F97 | LP(2) | C86-C89 | σ* | 6.11 | 1.01 | 0.07 |
| F98 | LP(2) | C85-C87 | σ* | 6.29 | 1.01 | 0.07 |
| F97 | LP(2) | C87-C89 | σ* | 7.56 | 0.98 | 0.08 |
| F98 | LP(2) | C87-C89 | σ* | 7.63 | 0.98 | 0.08 |
| N94 | LP(1) | C91-C93 | σ* | 12.67 | 1.04 | 0.10 |
| N96 | LP(1) | C91-C95 | σ* | 12.68 | 1.04 | 0.10 |
| N34 | LP(1) | C35-C36 | π* | 18.13 | 0.30 | 0.07 |
| S61 | LP(2) | C21-C62 | π* | 18.32 | 0.27 | 0.06 |
| N34 | LP(1) | C46-C48 | π* | 18.84 | 0.30 | 0.07 |
| O92 | LP(2) | C80-C81 | σ* | 19.57 | 0.75 | 0.11 |
| O92 | LP(2) | C81-C84 | σ* | 20.8 | 0.75 | 0.11 |
| S60 | LP(2) | C11-C12 | π* | 22.13 | 0.25 | 0.07 |
| S59 | LP(2) | C7-C8 | π* | 22.71 | 0.28 | 0.07 |
| S17 | LP(2) | C15-C16 | π* | 23.56 | 0.28 | 0.07 |
| S58 | LP(2) | C6-C19 | π* | 23.59 | 0.25 | 0.07 |
| S57 | LP(2) | C13-C14 | π* | 24.11 | 0.26 | 0.07 |
| N34 | LP(1) | C27-C31 | π* | 24.12 | 0.29 | 0.08 |
| S57 | LP(2) | C4-C5 | π* | 24.19 | 0.28 | 0.08 |
| S17 | LP(2) | C13-C14 | π* | 24.64 | 0.26 | 0.07 |
| S58 | LP(2) | C2-C3 | π* | 24.77 | 0.28 | 0.08 |
| S59 | LP(2) | C6-C19 | π* | 24.82 | 0.25 | 0.08 |
| S60 | LP(2) | C10-C20 | π* | 26.80 | 0.28 | 0.08 |
| S61 | LP(2) | C11-C12 | π* | 28.29 | 0.24 | 0.08 |

**Table S8**: The representative NBOs values for **FICD1.**

| **Compounds** | **Donor(*i*)** | **Type** | **Acceptor(*j*)** | **Type** | ***E* (2)**  **[*kcal/mol*]** | ***E*(*j*)-*E*(*i*)**  **[*a.u*]** |
| --- | --- | --- | --- | --- | --- | --- |
| C4-S79 | σ | C13-S17 | σ* | 0.51 | 0.90 | 0.02 |
| C51-C53 | π | C40-C42 | π* | 0.53 | 0.30 | 0.01 |
| C64-N65 | π | C66-N67 | π* | 0.66 | 0.47 | 0.02 |
| C66-N67 | π | C64-N65 | π* | 0.66 | 0.47 | 0.02 |
| C25-C26 | π | C25-C26 | π* | 0.77 | 0.32 | 0.01 |
| C5-C13 | σ | C3-C4 | σ* | 2.97 | 1.24 | 0.05 |
| C38-O62 | π | C22-C23 | π* | 3.57 | 0.43 | 0.04 |
| C58-H61 | σ | C53-C56 | σ* | 3.98 | 1.11 | 0.06 |
| C25-C26 | σ | C69-C70 | σ* | 4.97 | 1.30 | 0.07 |
| C72-C73 | σ | C68-C73 | σ* | 4.98 | 1.32 | 0.07 |
| C15-C16 | σ | C14-C15 | σ* | 4.99 | 1.28 | 0.07 |
| C84-H85 | σ | C21-S83 | σ* | 5.91 | 0.72 | 0.06 |
| C2-C19 | σ | C1-C2 | σ* | 5.93 | 1.16 | 0.07 |
| C13-C14 | σ | C5-C13 | σ* | 6.63 | 1.29 | 0.08 |
| C23-C37 | σ | C25-S76 | σ* | 6.86 | 0.88 | 0.07 |
| C15-C16 | σ | C14-S79 | σ* | 7.49 | 0.93 | 0.08 |
| C37-C63 | π | C22-C23 | π* | 7.54 | 0.33 | 0.07 |
| C2-C3 | σ | C4-S79 | σ* | 7.83 | 0.91 | 0.08 |
| C2-C3 | σ | C19-S81 | σ* | 8.52 | 0.90 | 0.08 |
| C7-C8 | σ | C6-S80 | σ* | 8.52 | 0.90 | 0.08 |
| C10-C20 | σ | C12-S83 | σ* | 8.57 | 0.92 | 0.08 |
| C37-C63 | π | C25-C26 | π* | 9.21 | 0.32 | 0.05 |
| C15-C16 | π | C27-C28 | π* | 10.80 | 0.32 | 0.06 |
| C22-C23 | π | C21-C84 | π* | 11.71 | 0.30 | 0.06 |
| C10-C20 | π | C7-C8 | π* | 14.05 | 0.31 | 0.06 |
| C2-C3 | π | C4-C5 | π* | 14.39 | 0.31 | 0.06 |
| C27-C28 | π | C15-C16 | π* | 14.60 | 0.29 | 0.06 |
| C25-C26 | π | C68-C69 | π* | 15.00 | 0.31 | 0.07 |
| C7-C8 | π | C6-C19 | π* | 15.02 | 0.28 | 0.06 |
| C15-C16 | π | C13-C14 | π* | 15.47 | 0.29 | 0.07 |
| C21-C84 | π | C11-C12 | π* | 15.53 | 0.27 | 0.06 |
| C4-C5 | π | C2-C3 | π* | 15.57 | 0.31 | 0.06 |
| C4-C5 | π | C13-C14 | π* | 15.57 | 0.29 | 0.07 |
| C13-C14 | π | C15-C16 | π* | 16.54 | 0.32 | 0.07 |
| C2-C3 | π | C6-C19 | π* | 17.29 | 0.28 | 0.07 |
| C72-C73 | π | C68-C69 | π* | 17.34 | 0.30 | 0.07 |
| C11-C12 | π | C10-C20 | π* | 17.43 | 0.31 | 0.07 |
| C7-C8 | π | C10-C20 | π* | 18.21 | 0.30 | 0.07 |
| C6-C19 | π | C2-C3 | π* | 18.28 | 0.32 | 0.07 |
| C68-C69 | π | C25-C26 | π* | 18.46 | 0.30 | 0.07 |
| C13-C14 | π | C4-C5 | π* | 18.56 | 0.32 | 0.07 |
| C72-C73 | π | C70-C71 | π* | 18.69 | 0.31 | 0.07 |
| C70-C71 | π | C68-C69 | π* | 18.85 | 0.30 | 0.07 |
| C29-C32 | π | C27-C28 | π* | 19.46 | 0.30 | 0.07 |
| C30-C34 | π | C29-C32 | π* | 19.67 | 0.30 | 0.07 |
| C70-C71 | π | C72-C73 | π* | 19.8 | 0.30 | 0.07 |
| C10-C20 | π | C11-C12 | π* | 20.18 | 0.27 | 0.07 |
| C68-C69 | π | C70-C71 | π* | 20.18 | 0.30 | 0.07 |
| C40-C42 | π | C41-C43 | π* | 20.24 | 0.30 | 0.07 |
| C41-C43 | π | C45-C47 | π* | 20.39 | 0.30 | 0.07 |
| C51-C53 | π | C52-C54 | π* | 20.47 | 0.30 | 0.07 |
| C52-C54 | π | C56-C58 | π* | 20.56 | 0.30 | 0.07 |
| C25-C26 | π | C38-O62 | π* | 20.63 | 0.33 | 0.07 |
| C6-C19 | π | C7-C8 | π* | 21.39 | 0.31 | 0.08 |
| C25-C26 | π | C37-C63 | π* | 21.61 | 0.31 | 0.08 |
| C45-C47 | π | C40-C42 | π* | 21.65 | 0.29 | 0.07 |
| C27-C28 | π | C29-C32 | π* | 21.76 | 0.30 | 0.07 |
| C29-C32 | π | C30-C34 | π* | 21.83 | 0.30 | 0.07 |
| C56-C58 | π | C51-C53 | π* | 21.87 | 0.29 | 0.07 |
| C27-C28 | π | C30-C34 | π* | 21.93 | 0.29 | 0.07 |
| C22-C23 | π | C38-O62 | π* | 22.66 | 0.31 | 0.08 |
| C51-C53 | π | C56-C58 | π* | 22.99 | 0.30 | 0.08 |
| C52-C54 | π | C51-C53 | π* | 23.05 | 0.29 | 0.08 |
| C41-C43 | π | C40-C42 | π* | 23.12 | 0.29 | 0.08 |
| C40-C42 | π | C45-C47 | π* | 23.26 | 0.30 | 0.08 |
| C56-C58 | π | C52-C54 | π* | 23.5 | 0.30 | 0.08 |
| C45-C47 | π | C41-C43 | π* | 23.64 | 0.30 | 0.08 |
| C68-C69 | π | C72-C73 | π* | 23.89 | 0.29 | 0.08 |
| C30-C34 | π | C27-C28 | π* | 23.91 | 0.30 | 0.08 |
| C11-C12 | π | C21-C84 | π* | 25.16 | 0.30 | 0.08 |
| C22-C23 | π | C37-C63 | π* | 27.00 | 0.29 | 0.08 |
| C21-C84 | π | C22-C23 | π* | 29.69 | 0.31 | 0.09 |
| S82 | LP(1) | C11-C84 | σ* | 0.62 | 1.24 | 0.03 |
| O62 | LP(2) | C84-H85 | σ* | 2.31 | 0.72 | 0.04 |
| S82 | LP(1) | C10-C20 | σ* | 2.97 | 1.21 | 0.06 |
| Cl78 | LP(2) | C72-C73 | σ* | 4.39 | 0.90 | 0.06 |
| Cl78 | LP(2) | C71-C72 | σ* | 5.42 | 0.86 | 0.06 |
| Cl77 | LP(2) | C71-C72 | σ* | 5.44 | 0.86 | 0.06 |
| N65 | LP(1) | C63-C64 | σ* | 12.64 | 1.05 | 0.10 |
| N67 | LP(1) | C63-C66 | σ* | 12.65 | 1.04 | 0.10 |
| Cl77 | LP(3) | C70-C71 | π* | 14.21 | 0.35 | 0.07 |
| Cl78 | LP(3) | C72-C73 | π* | 14.50 | 0.34 | 0.07 |
| N39 | LP(1) | C51-C53 | π* | 17.53 | 0.30 | 0.07 |
| S83 | LP(2) | C21-C84 | π* | 18.58 | 0.27 | 0.07 |
| N39 | LP(1) | C40-C42 | π* | 19.59 | 0.30 | 0.07 |
| O62 | LP(2) | C23-C38 | σ* | 19.79 | 0.74 | 0.11 |
| O62 | LP(2) | C26-C38 | σ* | 20.89 | 0.75 | 0.11 |
| S76 | LP(2) | C68-C69 | π* | 21.91 | 0.27 | 0.07 |
| S82 | LP(2) | C11-C12 | π* | 22.34 | 0.25 | 0.07 |
| S81 | LP(2) | C7-C8 | π* | 22.93 | 0.28 | 0.07 |
| N39 | LP(1) | C30-C34 | π* | 23.34 | 0.29 | 0.08 |
| S17 | LP(2) | C15-C16 | π* | 23.60 | 0.28 | 0.07 |
| S80 | LP(2) | C6-C19 | π* | 23.63 | 0.25 | 0.07 |
| S79 | LP(2) | C13-C14 | π* | 24.01 | 0.26 | 0.07 |
| S79 | LP(2) | C4-C5 | π* | 24.20 | 0.28 | 0.08 |
| S80 | LP(2) | C2-C3 | π* | 24.63 | 0.28 | 0.08 |
| S81 | LP(2) | C6-C19 | π* | 24.74 | 0.25 | 0.08 |
| S17 | LP(2) | C13-C14 | π* | 24.77 | 0.26 | 0.08 |
| S82 | LP(2) | C10-C20 | π* | 26.69 | 0.28 | 0.08 |
| S76 | LP(2) | C25-C26 | π* | 26.77 | 0.28 | 0.08 |
| S83 | LP(2) | C11-C12 | π* | 28.01 | 0.25 | 0.08 |

**Table S9**: The representative NBOs values for **FICD2.**

| **Compounds** | **Donor(*i*)** | **Type** | **Acceptor(*j*)** | **Type** | ***E* (2)**  **[*kcal/mol*]** | ***E*(*j*)-*E*(*i*)**  **[*a.u*]** |
| --- | --- | --- | --- | --- | --- | --- |
| C4-S77 | σ | C13-S17 | σ* | 0.51 | 0.90 | 0.02 |
| C2-C3 | π | C2-C3 | π* | 0.55 | 0.31 | 0.01 |
| C23-C38 | σ | C22-H24 | σ* | 1.99 | 1.07 | 0.04 |
| C1-C92 | σ | C1-C2 | σ* | 2.00 | 1.05 | 0.04 |
| C7-C8 | σ | C8-C9 | σ* | 2.98 | 1.13 | 0.05 |
| C26-C69 | σ | C26-C38 | σ* | 3.99 | 1.17 | 0.06 |
| C58-H61 | σ | C53-C56 | σ* | 3.99 | 1.11 | 0.06 |
| C15-C16 | σ | C14-C15 | σ* | 4.96 | 1.28 | 0.07 |
| C14-C15 | σ | C16-C27 | σ* | 4.97 | 1.23 | 0.07 |
| C22-C23 | σ | C23-C37 | σ* | 4.99 | 1.23 | 0.07 |
| C72-C73 | σ | C71-C72 | σ* | 5.82 | 1.28 | 0.08 |
| C82-H83 | σ | C21-S81 | σ* | 5.85 | 0.71 | 0.06 |
| C68-S76 | σ | C25-C37 | σ* | 5.87 | 1.15 | 0.07 |
| C2-C19 | σ | C1-C2 | σ* | 5.99 | 1.16 | 0.08 |
| N100-O101 | π | N100-O101 | π* | 6.58 | 0.40 | 0.05 |
| C13-C14 | σ | C5-C13 | σ* | 6.65 | 1.29 | 0.08 |
| C23-C37 | σ | C25-S76 | σ* | 6.79 | 0.88 | 0.07 |
| C11-C12 | σ | C9-C10 | σ* | 7.53 | 1.15 | 0.08 |
| C37-C63 | π | C22-C23 | π* | 7.60 | 0.33 | 0.05 |
| C2-C3 | σ | C4-S77 | σ* | 7.84 | 0.91 | 0.08 |
| C2-C3 | σ | C19-S79 | σ* | 8.51 | 0.90 | 0.08 |
| C7-C8 | σ | C6-S78 | σ* | 8.58 | 0.90 | 0.08 |
| C10-C20 | σ | C12-S81 | σ* | 8.62 | 0.92 | 0.08 |
| C37-C63 | π | C25-C26 | π* | 9.50 | 0.32 | 0.05 |
| C15-C16 | π | C27-C28 | π* | 10.47 | 0.32 | 0.06 |
| C22-C23 | π | C21-C82 | π* | 11.85 | 0.30 | 0.06 |
| C71-C72 | π | N100-O101 | π* | 13.16 | 0.19 | 0.05 |
| C10-C20 | π | C7-C8 | π* | 14.23 | 0.31 | 0.06 |
| C27-C28 | π | C15-C16 | π* | 14.24 | 0.29 | 0.06 |
| C2-C3 | π | C4-C5 | π* | 14.29 | 0.31 | 0.06 |
| C7-C8 | π | C6-C19 | π* | 14.92 | 0.28 | 0.06 |
| C15-C16 | π | C13-C14 | π* | 15.50 | 0.29 | 0.07 |
| C4-C5 | π | C13-C14 | π* | 15.54 | 0.29 | 0.06 |
| C4-C5 | π | C2-C3 | π* | 15.78 | 0.31 | 0.07 |
| C21-C82 | π | C11-C12 | π* | 15.79 | 0.27 | 0.06 |
| C13-C14 | π | C15-C16 | π* | 16.45 | 0.32 | 0.07 |
| C71-C72 | π | N103-O104 | π* | 16.81 | 0.17 | 0.05 |
| C11-C12 | π | C10-C20 | π* | 17.64 | 0.31 | 0.07 |
| C2-C3 | π | C6-C19 | π* | 17.74 | 0.28 | 0.07 |
| C6-C19 | π | C2-C3 | π* | 18.26 | 0.31 | 0.07 |
| C13-C14 | π | C4-C5 | π* | 18.77 | 0.32 | 0.07 |
| C7-C8 | π | C10-C20 | π* | 18.79 | 0.30 | 0.07 |
| C25-C26 | π | C69-C70 | π* | 18.89 | 0.32 | 0.07 |
| C29-C32 | π | C27-C28 | π* | 19.62 | 0.30 | 0.07 |
| C30-C34 | π | C29-C32 | π* | 19.78 | 0.30 | 0.07 |
| C25-C26 | π | C37-C63 | π* | 20.04 | 0.32 | 0.07 |
| C25-C26 | π | C38-O62 | π* | 20.14 | 0.33 | 0.07 |
| C40-C42 | π | C41-C43 | π* | 20.16 | 0.30 | 0.07 |
| C41-C43 | π | C45-C47 | π* | 20.26 | 0.30 | 0.07 |
| C51-C53 | π | C52-C54 | π* | 20.45 | 0.30 | 0.07 |
| C52-C54 | π | C56-C58 | π* | 20.58 | 0.30 | 0.07 |
| C69-C70 | π | C68-C73 | π* | 20.69 | 0.27 | 0.07 |
| C69-C70 | π | C25-C26 | π* | 20.78 | 0.29 | 0.07 |
| C10-C20 | π | C11-C12 | π* | 20.94 | 0.27 | 0.07 |
| C68-C73 | π | C69-C70 | π* | 20.97 | 0.31 | 0.07 |
| C45-C47 | π | C40-C42 | π* | 21.58 | 0.29 | 0.07 |
| C27-C28 | π | C29-C32 | π* | 21.71 | 0.30 | 0.07 |
| C6-C19 | π | C7-C8 | π* | 21.76 | 0.31 | 0.08 |
| C56-C58 | π | C51-C53 | π* | 21.87 | 0.29 | 0.07 |
| C29-C32 | π | C30-C34 | π* | 21.89 | 0.30 | 0.07 |
| C71-C72 | π | C69-C70 | π* | 21.92 | 0.32 | 0.08 |
| C27-C28 | π | C30-C34 | π* | 21.95 | 0.29 | 0.07 |
| C68-C73 | π | C71-C72 | π* | 22.37 | 0.28 | 0.07 |
| C51-C53 | π | C56-C58 | π* | 23.01 | 0.30 | 0.08 |
| C52-C54 | π | C51-C53 | π* | 23.06 | 0.29 | 0.08 |
| C41-C43 | π | C40-C42 | π* | 23.09 | 0.29 | 0.08 |
| C40-C42 | π | C45-C47 | π* | 23.39 | 0.30 | 0.08 |
| C56-C58 | π | C52-C54 | π* | 23.46 | 0.30 | 0.08 |
| C22-C23 | π | C38-O62 | π* | 23.49 | 0.30 | 0.08 |
| C45-C47 | π | C41-C43 | π* | 23.74 | 0.30 | 0.08 |
| C30-C34 | π | C27-C28 | π* | 23.86 | 0.30 | 0.08 |
| C71-C72 | π | C68-C73 | π* | 23.94 | 0.30 | 0.08 |
| C11-C12 | π | C21-C82 | π* | 26.23 | 0.30 | 0.08 |
| C69-C70 | π | C71-C72 | π* | 27.98 | 0.26 | 0.08 |
| C22-C23 | π | C37-C63 | π* | 28.61 | 0.29 | 0.08 |
| C21-C82 | π | C22-C23 | π* | 31.64 | 0.30 | 0.08 |
| S80 | LP(1) | C11-C82 | σ* | 0.63 | 1.25 | 0.01 |
| N39 | LP(1) | C40-C41 | σ* | 2.95 | 0.85 | 0.05 |
| N39 | LP(1) | C51-C52 | σ* | 3.45 | 0.85 | 0.05 |
| O105 | LP(1) | C72-N103 | σ* | 4.02 | 1.09 | 0.06 |
| N65 | LP(1) | C63-C64 | σ* | 12.66 | 1.04 | 0.10 |
| N67 | LP(1) | C63-C66 | σ* | 12.68 | 1.04 | 0.10 |
| O105 | LP(2) | C72-N103 | σ* | 15.83 | 0.58 | 0.09 |
| O102 | LP(2) | C71-N100 | σ* | 15.87 | 0.58 | 0.09 |
| O101 | LP(2) | C71-N100 | σ* | 15.94 | 0.58 | 0.09 |
| O104 | LP(2) | C72-N103 | σ* | 15.98 | 0.58 | 0.09 |
| N39 | LP(1) | C51-C53 | π* | 17.45 | 0.30 | 0.07 |
| S81 | LP(2) | C21-C82 | π* | 18.19 | 0.27 | 0.06 |
| O62 | LP(2) | C23-C38 | σ* | 19.64 | 0.74 | 0.11 |
| O105 | LP(2) | N103-O104 | σ* | 20.43 | 0.76 | 0.11 |
| O101 | LP(2) | N100-O102 | σ* | 20.49 | 0.76 | 0.11 |
| O102 | LP(2) | N100-O101 | σ* | 20.51 | 0.74 | 0.11 |
| N39 | LP(1) | C40-C42 | π* | 20.53 | 0.30 | 0.07 |
| O104 | LP(2) | N103-O105 | σ* | 20.71 | 0.75 | 0.11 |
| O62 | LP(2) | C26-C38 | σ* | 21.28 | 0.75 | 0.11 |
| N39 | LP(1) | C30-C34 | π* | 22.17 | 0.29 | 0.07 |
| S80 | LP(2) | C11-C12 | π* | 22.19 | 0.24 | 0.07 |
| S79 | LP(2) | C7-C8 | π* | 22.97 | 0.28 | 0.07 |
| S78 | LP(2) | C6-C19 | π* | 23.47 | 0.25 | 0.07 |
| S17 | LP(2) | C15-C16 | π* | 23.69 | 0.28 | 0.07 |
| S77 | LP(2) | C13-C14 | π* | 24.03 | 0.26 | 0.07 |
| S77 | LP(2) | C4-C5 | π* | 24.11 | 0.28 | 0.08 |
| S76 | LP(2) | C68-C73 | π* | 24.16 | 0.27 | 0.07 |
| S17 | LP(2) | C13-C14 | π* | 24.85 | 0.26 | 0.08 |
| S78 | LP(2) | C2-C3 | π* | 24.87 | 0.28 | 0.08 |
| S79 | LP(2) | C6-C19 | π* | 25.00 | 0.25 | 0.08 |
| S76 | LP(2) | C25-C26 | π* | 26.84 | 0.28 | 0.08 |
| S80 | LP(2) | C10-C20 | π* | 26.85 | 0.28 | 0.08 |
| S81 | LP(2) | C11-C12 | π* | 28.18 | 0.24 | 0.08 |

**Table S10**: The representative NBOs values for **FICD3.**

| **Compounds** | **Donor(*i*)** | **Type** | **Acceptor(*j*)** | **Type** | ***E* (2)**  **[*kcal/mol*]** | ***E*(*j*)-*E*(*i*)**  **[*a.u*]** |
| --- | --- | --- | --- | --- | --- | --- |
| C51-C53 | π | C40-C42 | π* | 0.50 | 0.30 | 0.01 |
| C4-S77 | σ | C13-S17 | σ* | 0.51 | 0.90 | 0.02 |
| C68-C73 | σ | C72-C101 | σ* | 2.96 | 1.11 | 0.05 |
| C5-C13 | σ | C3-C4 | σ* | 2.97 | 1.23 | 0.05 |
| C58-H61 | σ | C52-C54 | σ* | 3.98 | 1.11 | 0.06 |
| C58-H61 | σ | C53-C56 | σ* | 3.98 | 1.11 | 0.06 |
| C68-S76 | σ | C69-C70 | σ* | 3.98 | 1.25 | 0.06 |
| C15-C16 | σ | C14-C15 | σ* | 4.97 | 1.28 | 0.07 |
| C14-C15 | σ | C16-C27 | σ* | 4.98 | 1.23 | 0.07 |
| C22-C23 | σ | C23-C37 | σ* | 4.98 | 1.23 | 0.07 |
| C82-H83 | σ | C21-S81 | σ* | 5.86 | 0.72 | 0.06 |
| C68-S76 | σ | C25-C37 | σ* | 5.90 | 1.15 | 0.07 |
| C70-C71 | σ | C71-C72 | σ* | 5.97 | 1.27 | 0.08 |
| C2-C19 | σ | C1-C2 | σ* | 5.98 | 1.16 | 0.07 |
| C72-C73 | π | C101-F105 | σ* | 6.63 | 0.55 | 0.06 |
| C13-C14 | σ | C5-C13 | σ* | 6.66 | 1.29 | 0.08 |
| C70-C71 | π | C100-F104 | σ* | 6.69 | 0.55 | 0.06 |
| C23-C37 | σ | C25-S76 | σ* | 6.81 | 0.88 | 0.07 |
| C6-C19 | σ | C8-C9 | σ* | 7.42 | 1.14 | 0.08 |
| C15-C16 | σ | C14-S77 | σ* | 7.48 | 0.93 | 0.08 |
| C11-C12 | σ | C9-C10 | σ* | 7.49 | 1.15 | 0.08 |
| C37-C63 | π | C22-C23 | σ* | 7.56 | 0.33 | 0.05 |
| C2-C3 | σ | C4-S77 | σ* | 7.84 | 0.91 | 0.08 |
| C7-C8 | σ | C20-S80 | σ* | 8.00 | 0.91 | 0.08 |
| C63-C64 | σ | C64-N65 | σ* | 8.33 | 1.62 | 0.10 |
| C4-C5 | σ | C13-S17 | σ* | 8.45 | 0.92 | 0.08 |
| C2-C3 | σ | C19-S79 | σ* | 8.50 | 0.90 | 0.08 |
| C7-C8 | σ | C6-S78 | σ* | 8.56 | 0.90 | 0.08 |
| C10-C20 | σ | C12-S81 | σ* | 8.62 | 0.92 | 0.08 |
| C37-C63 | π | C25-C26 | π* | 9.30 | 0.32 | 0.05 |
| C15-C16 | π | C27-C28 | π* | 11.37 | 0.32 | 0.06 |
| C22-C23 | π | C21-C82 | π* | 11.74 | 0.30 | 0.06 |
| C10-C20 | π | C7-C8 | π* | 14.12 | 0.31 | 0.06 |
| C2-C3 | π | C4-C5 | π* | 14.33 | 0.31 | 0.06 |
| C7-C8 | π | C6-C19 | π* | 14.94 | 0.28 | 0.06 |
| C15-C16 | π | C13-C14 | π* | 15.49 | 0.29 | 0.07 |
| C27-C28 | π | C15-C16 | π* | 15.51 | 0.29 | 0.06 |
| C4-C5 | π | C2-C3 | π* | 15.60 | 0.31 | 0.07 |
| C4-C5 | π | C13-C14 | π* | 15.64 | 0.29 | 0.07 |
| C21-C82 | π | C11-C12 | π* | 15.66 | 0.27 | 0.06 |
| C25-C26 | π | C68-C69 | π* | 15.66 | 0.31 | 0.07 |
| C13-C14 | π | C15-C16 | π* | 16.54 | 0.32 | 0.07 |
| C11-C12 | π | C10-C20 | π* | 17.44 | 0.31 | 0.07 |
| C2-C3 | π | C6-C19 | π* | 17.51 | 0.28 | 0.07 |
| C72-C73 | π | C68-C69 | π* | 17.51 | 0.30 | 0.07 |
| C68-C69 | π | C25-C26 | π* | 17.70 | 0.30 | 0.07 |
| C6-C19 | π | C2-C3 | π* | 18.34 | 0.32 | 0.07 |
| C7-C8 | π | C10-C20 | π* | 18.49 | 0.30 | 0.07 |
| C13-C14 | π | C4-C5 | π* | 18.69 | 0.32 | 0.07 |
| C68-C69 | π | C70-C71 | π* | 18.84 | 0.31 | 0.07 |
| C72-C73 | π | C70-C71 | π* | 19.43 | 0.31 | 0.07 |
| C29-C32 | π | C27-C28 | π* | 19.45 | 0.30 | 0.07 |
| C70-C71 | π | C68-C69 | π* | 19.49 | 0.29 | 0.07 |
| C30-C34 | π | C29-C32 | π* | 19.68 | 0.30 | 0.07 |
| C40-C42 | π | C41-C43 | π* | 20.21 | 0.30 | 0.07 |
| C41-C43 | π | C45-C47 | π* | 20.33 | 0.30 | 0.07 |
| C25-C26 | π | C38-O62 | π* | 20.41 | 0.33 | 0.07 |
| C51-C53 | π | C52-C54 | π* | 20.44 | 0.30 | 0.07 |
| C10-C20 | π | C11-C12 | π* | 20.49 | 0.27 | 0.07 |
| C52-C54 | π | C56-C58 | π* | 20.53 | 0.30 | 0.07 |
| C70-C71 | π | C72-C73 | π* | 20.55 | 0.31 | 0.07 |
| C25-C26 | π | C37-C63 | π* | 20.78 | 0.32 | 0.07 |
| C6-C19 | π | C7-C8 | π* | 21.53 | 0.31 | 0.08 |
| C45-C47 | π | C40-C42 | π* | 21.64 | 0.29 | 0.08 |
| C27-C28 | π | C29-C32 | π* | 21.69 | 0.30 | 0.08 |
| C29-C32 | π | C30-C34 | π* | 21.75 | 0.30 | 0.08 |
| C56-C58 | π | C51-C53 | π* | 21.87 | 0.29 | 0.08 |
| C27-C28 | π | C30-C34 | π* | 21.99 | 0.29 | 0.08 |
| C68-C69 | π | C72-C73 | π* | 22.26 | 0.30 | 0.08 |
| C22-C23 | π | C38-O62 | π* | 22.99 | 0.30 | 0.08 |
| C51-C53 | π | C56-C58 | π* | 22.99 | 0.30 | 0.08 |
| C52-C54 | π | C51-C53 | π* | 23.02 | 0.29 | 0.08 |
| C41-C43 | π | C40-C42 | π* | 23.07 | 0.29 | 0.08 |
| C40-C42 | π | C45-C47 | π* | 23.31 | 0.30 | 0.08 |
| C56-C58 | π | C52-C54 | π* | 23.48 | 0.30 | 0.08 |
| C45-C47 | π | C41-C43 | π* | 23.67 | 0.30 | 0.08 |
| C30-C34 | π | C27-C28 | π* | 23.93 | 0.30 | 0.08 |
| C11-C12 | π | C21-C82 | π* | 25.63 | 0.30 | 0.08 |
| C22-C23 | π | C37-C63 | π* | 27.62 | 0.29 | 0.08 |
| C21-C82 | π | C22-C23 | π* | 30.50 | 0.30 | 0.09 |
| F102 | LP(1) | C71-C100 | σ* | 0.51 | 1.46 | 0.03 |
| S76 | LP(1) | C68-C69 | σ* | 2.86 | 1.21 | 0.05 |
| S80 | LP(1) | C10-C20 | σ* | 2.99 | 1.21 | 0.05 |
| F107 | LP(2) | C101-F106 | σ* | 3.93 | 0.72 | 0.05 |
| F103 | LP(2) | C100-F102 | σ* | 4.46 | 0.71 | 0.05 |
| F107 | LP(2) | C101-F105 | σ* | 5.45 | 0.72 | 0.06 |
| F102 | LP(2) | C71-C100 | σ* | 6.82 | 0.82 | 0.07 |
| N65 | LP(1) | C63-C64 | σ* | 12.65 | 1.04 | 0.10 |
| N67 | LP(1) | C63-C66 | σ* | 12.66 | 1.04 | 0.10 |
| N39 | LP(1) | C51-C53 | π* | 17.72 | 0.30 | 0.07 |
| S81 | LP(2) | C21-C82 | π* | 18.44 | 0.27 | 0.06 |
| O62 | LP(2) | C23-C38 | σ* | 19.77 | 0.74 | 0.11 |
| N39 | LP(1) | C40-C42 | π* | 20.05 | 0.30 | 0.07 |
| O62 | LP(2) | C26-C38 | σ* | 21.03 | 0.75 | 0.11 |
| S80 | LP(2) | C11-C12 | π* | 22.33 | 0.25 | 0.07 |
| S76 | LP(2) | C68-C69 | π* | 22.77 | 0.27 | 0.07 |
| S79 | LP(2) | C7-C8 | π* | 22.95 | 0.28 | 0.07 |
| N39 | LP(1) | C30-C34 | π* | 23.27 | 0.29 | 0.08 |
| S78 | LP(2) | C6-C19 | π* | 23.52 | 0.25 | 0.07 |
| S17 | LP(2) | C15-C16 | π* | 23.58 | 0.28 | 0.07 |
| S77 | LP(2) | C13-C14 | π* | 23.95 | 0.26 | 0.07 |
| S77 | LP(2) | C4-C5 | π* | 24.16 | 0.28 | 0.08 |
| S78 | LP(2) | C2-C3 | π* | 24.62 | 0.28 | 0.08 |
| S17 | LP(2) | C13-C14 | π* | 24.86 | 0.26 | 0.08 |
| S79 | LP(2) | C6-C19 | π* | 24.89 | 0.25 | 0.08 |
| S76 | LP(2) | C25-C26 | π* | 26.69 | 0.28 | 0.08 |
| S80 | LP(2) | C10-C20 | π* | 26.86 | 0.28 | 0.08 |
| S81 | LP(2) | C11-C12 | π* | 28.07 | 0.24 | 0.08 |

**Table S11**: The representative NBOs values for **FICD4.**

| **Compounds** | **Donor(*i*)** | **Type** | **Acceptor(*j*)** | **Type** | ***E* (2)**  **[*kcal/mol*]** | ***E*(*j*)-*E*(*i*)**  **[*a.u*]** |
| --- | --- | --- | --- | --- | --- | --- |
| C51-C53 | π | C40-C42 | π* | 0.50 | 0.30 | 0.01 |
| C4-S77 | σ | C13-S17 | σ* | 0.51 | 0.90 | 0.02 |
| C71-C103 | σ | C70-C71 | σ* | 2.97 | 1.26 | 0.06 |
| C72-C100 | σ | C70-C71 | σ* | 2.97 | 1.26 | 0.06 |
| C58-H61 | σ | C52-C54 | σ* | 3.98 | 1.11 | 0.06 |
| C58-H61 | σ | C53-C56 | σ* | 3.98 | 1.11 | 0.06 |
| C25-C26 | σ | C69-C70 | σ* | 4.97 | 1.31 | 0.07 |
| C15-C16 | σ | C14-C15 | σ* | 4.98 | 1.28 | 0.07 |
| C73-H75 | σ | C71-C72 | σ* | 4.98 | 1.08 | 0.07 |
| C63-C66 | σ | C37-C63 | σ* | 5.86 | 1.33 | 0.08 |
| C68-S76 | σ | C25-C37 | σ* | 5.90 | 1.15 | 0.07 |
| C82-H83 | σ | C21-S81 | σ* | 5.90 | 0.72 | 0.06 |
| C2-C19 | σ | C1-C2 | σ* | 5.95 | 1.16 | 0.07 |
| C63-C64 | σ | C37-C63 | σ* | 6.27 | 1.33 | 0.08 |
| C22-C23 | σ | C21-C22 | σ* | 6.49 | 1.31 | 0.08 |
| C13-C14 | σ | C5-C13 | σ* | 6.65 | 1.29 | 0.08 |
| C23-C37 | σ | C25-S76 | σ* | 6.85 | 0.88 | 0.07 |
| C2-C3 | σ | C4-S77 | σ* | 7.84 | 0.91 | 0.08 |
| C7-C8 | σ | C20-S80 | σ* | 8.00 | 0.91 | 0.07 |
| C63-C66 | σ | C66-N67 | σ* | 8.10 | 1.61 | 0.10 |
| C66-N67 | σ | C63-C66 | σ* | 8.10 | 1.57 | 0.10 |
| C4-C5 | σ | C3-S78 | σ* | 8.11 | 0.90 | 0.08 |
| C2-C3 | σ | C19-S79 | σ* | 8.51 | 0.90 | 0.08 |
| C7-C8 | σ | C6-S78 | σ* | 8.53 | 0.90 | 0.08 |
| C10-C20 | σ | C12-S81 | σ* | 8.58 | 0.92 | 0.08 |
| C37-C63 | π | C25-C26 | π* | 9.19 | 0.32 | 0.05 |
| C15-C16 | π | C27-C28 | π* | 11.44 | 0.32 | 0.06 |
| C22-C23 | π | C21-C82 | π* | 11.72 | 0.30 | 0.06 |
| C10-C20 | π | C7-C8 | π* | 14.06 | 0.31 | 0.06 |
| C2-C3 | π | C4-C5 | π* | 14.36 | 0.31 | 0.06 |
| C25-C26 | π | C68-C69 | π* | 14.87 | 0.31 | 0.06 |
| C7-C8 | π | C6-C19 | π* | 14.99 | 0.28 | 0.06 |
| C15-C16 | π | C13-C14 | π* | 15.45 | 0.29 | 0.07 |
| C4-C5 | π | C2-C3 | π* | 15.52 | 0.31 | 0.06 |
| C27-C28 | π | C15-C16 | π* | 15.52 | 0.29 | 0.06 |
| C21-C82 | π | C11-C12 | π* | 15.53 | 0.27 | 0.06 |
| C4-C5 | π | C13-C14 | π* | 15.66 | 0.29 | 0.07 |
| C13-C14 | π | C15-C16 | π* | 16.57 | 0.32 | 0.07 |
| C2-C3 | π | C6-C19 | π* | 17.32 | 0.28 | 0.07 |
| C11-C12 | π | C10-C20 | π* | 17.41 | 0.31 | 0.07 |
| C7-C8 | π | C10-C20 | π* | 18.18 | 0.30 | 0.07 |
| C6-C19 | π | C2-C3 | π* | 18.34 | 0.32 | 0.07 |
| C68-C69 | π | C25-C26 | π* | 18.57 | 0.30 | 0.07 |
| C13-C14 | π | C4-C5 | π* | 18.59 | 0.32 | 0.07 |
| C72-C73 | π | C70-C71 | π* | 18.65 | 0.31 | 0.07 |
| C68-C69 | π | C70-C71 | π* | 18.82 | 0.31 | 0.07 |
| C68-C69 | π | C72-C73 | π* | 19.39 | 0.31 | 0.07 |
| C29-C32 | π | C27-C28 | π* | 19.46 | 0.30 | 0.07 |
| C30-C34 | π | C29-C32 | π* | 19.7 | 0.30 | 0.07 |
| C70-C71 | π | C103-O104 | π* | 19.71 | 0.30 | 0.07 |
| C10-C20 | π | C11-C12 | π* | 20.18 | 0.27 | 0.07 |
| C40-C42 | π | C41-C43 | π* | 20.22 | 0.30 | 0.07 |
| C72-C73 | π | C68-C69 | π* | 20.25 | 0.28 | 0.07 |
| C41-C43 | π | C45-C47 | π* | 20.34 | 0.30 | 0.07 |
| C51-C53 | π | C52-C54 | π* | 20.43 | 0.30 | 0.07 |
| C70-C71 | π | C68-C69 | π* | 20.44 | 0.28 | 0.07 |
| C25-C26 | π | C38-O62 | π* | 20.47 | 0.33 | 0.07 |
| C52-C54 | π | C56-C58 | π* | 20.51 | 0.30 | 0.07 |
| C6-C19 | π | C7-C8 | π* | 21.35 | 0.31 | 0.08 |
| C25-C26 | π | C37-C63 | π* | 21.58 | 0.31 | 0.08 |
| C45-C47 | π | C40-C42 | π* | 21.65 | 0.29 | 0.08 |
| C27-C28 | π | C29-C32 | π* | 21.68 | 0.30 | 0.08 |
| C29-C32 | π | C30-C34 | π* | 21.74 | 0.30 | 0.08 |
| C70-C71 | π | C72-C73 | π* | 21.75 | 0.31 | 0.08 |
| C56-C58 | π | C51-C53 | π* | 21.85 | 0.29 | 0.08 |
| C27-C28 | π | C30-C34 | π* | 22.02 | 0.29 | 0.08 |
| C22-C23 | π | C38-O62 | π* | 22.63 | 0.31 | 0.08 |
| C51-C53 | π | C56-C58 | π* | 23.03 | 0.30 | 0.08 |
| C52-C54 | π | C51-C53 | π* | 23.03 | 0.29 | 0.08 |
| C41-C43 | π | C40-C42 | π* | 23.06 | 0.29 | 0.08 |
| C40-C42 | π | C45-C47 | π* | 23.29 | 0.30 | 0.08 |
| C56-C58 | π | C52-C54 | π* | 23.51 | 0.30 | 0.08 |
| C45-C47 | π | C41-C43 | π* | 23.66 | 0.30 | 0.08 |
| C30-C34 | π | C27-C28 | π* | 23.88 | 0.30 | 0.08 |
| C11-C12 | π | C21-C82 | π* | 25.14 | 0.30 | 0.08 |
| C22-C23 | π | C37-C63 | π* | 26.97 | 0.29 | 0.08 |
| C21-C82 | π | C22-C23 | π* | 29.64 | 0.31 | 0.09 |
| O105 | LP(1) | C110-H111 | σ* | 0.52 | 0.96 | 0.02 |
| O104 | LP(1) | C100-O101 | π* | 0.57 | 0.76 | 0.02 |
| N39 | LP(1) | C32-C34 | σ* | 2.78 | 0.84 | 0.05 |
| S76 | LP(1) | C68-C69 | σ* | 2.89 | 1.21 | 0.05 |
| N39 | LP(1) | C51-C52 | σ* | 3.36 | 0.85 | 0.05 |
| N39 | LP(1) | C51-C53 | σ* | 3.38 | 0.85 | 0.05 |
| O105 | LP(2) | C110-H112 | σ* | 4.87 | 0.72 | 0.06 |
| O105 | LP(2) | C110-H111 | σ* | 4.89 | 0.72 | 0.06 |
| O102 | LP(2) | C106-H108 | σ* | 5.23 | 0.71 | 0.06 |
| O105 | LP(1) | C103-O104 | σ* | 7.29 | 1.19 | 0.08 |
| O102 | LP(1) | C100-O101 | σ* | 7.40 | 1.19 | 0.08 |
| N65 | LP(1) | C63-C64 | σ* | 12.64 | 1.04 | 0.10 |
| N67 | LP(1) | C63-C66 | σ* | 12.65 | 1.04 | 0.10 |
| N39 | LP(1) | C51-C53 | π* | 17.95 | 0.30 | 0.07 |
| O104 | LP(2) | C71-C103 | σ* | 18.09 | 0.72 | 0.10 |
| S81 | LP(2) | C21-C82 | π* | 18.62 | 0.27 | 0.07 |
| O101 | LP(2) | C72-C100 | σ* | 19.43 | 0.71 | 0.11 |
| O62 | LP(2) | C23-C38 | σ* | 19.84 | 0.74 | 0.11 |
| N39 | LP(1) | C40-C42 | π* | 19.98 | 0.30 | 0.07 |
| O62 | LP(2) | C26-C38 | σ* | 20.92 | 0.75 | 0.11 |
| S80 | LP(2) | C11-C12 | π* | 22.34 | 0.25 | 0.07 |
| S76 | LP(2) | C68-C69 | π* | 22.76 | 0.27 | 0.07 |
| S79 | LP(2) | C7-C8 | π* | 22.96 | 0.28 | 0.07 |
| N39 | LP(1) | C30-C34 | π* | 23.23 | 0.29 | 0.08 |
| S17 | LP(2) | C15-C16 | π* | 23.53 | 0.28 | 0.07 |
| S78 | LP(2) | C6-C19 | π* | 23.60 | 0.25 | 0.07 |
| S77 | LP(2) | C13-C14 | π* | 23.92 | 0.26 | 0.07 |
| S77 | LP(2) | C4-C5 | π* | 24.19 | 0.28 | 0.08 |
| S78 | LP(2) | C2-C3 | π* | 24.58 | 0.28 | 0.08 |
| S79 | LP(2) | C6-C19 | π* | 24.78 | 0.25 | 0.08 |
| S17 | LP(2) | C13-C14 | π* | 24.84 | 0.26 | 0.08 |
| S76 | LP(2) | C25-C26 | π* | 26.41 | 0.28 | 0.08 |
| S80 | LP(2) | C10-C20 | π* | 26.68 | 0.28 | 0.08 |
| S81 | LP(2) | C11-C12 | π* | 28.07 | 0.25 | 0.08 |
| O104 | LP(2) | C103-O105 | σ* | 32.99 | 0.67 | 0.14 |
| O101 | LP(2) | C100-O102 | σ* | 33.16 | 0.68 | 0.14 |
| O102 | LP(2) | C100-O101 | π* | 48.05 | 0.38 | 0.12 |
| O105 | LP(2) | C103-O104 | π* | 50.34 | 0.37 | 0.12 |

**Table S12:** The representative NBOs values for **FICD5.**

| **Compounds** | **Donor(*i*)** | **Type** | **Acceptor(*j*)** | **Type** | ***E* (2)**  **[*kcal/mol*]** | ***E*(*j*)-*E*(*i*)**  **[*a.u*]** |
| --- | --- | --- | --- | --- | --- | --- |
| C4-S77 | σ | C13-S17 | σ* | 0.51 | 0.9 | 0.02 |
| C2-C3 | π | C2-C3 | π* | 0.54 | 0.31 | 0.01 |
| C2-C19 | σ | C6-C8 | σ* | 2.91 | 1.3 | 0.06 |
| C26-C69 | σ | C25-C37 | σ* | 2.94 | 1.18 | 0.05 |
| C5-C13 | σ | C3-C4 | σ* | 2.97 | 1.24 | 0.05 |
| C68-S76 | σ | C69-C70 | σ* | 3.98 | 1.24 | 0.06 |
| C25-C37 | σ | C37-C63 | σ* | 3.99 | 1.28 | 0.06 |
| C23-C37 | σ | C22-C23 | σ* | 4.93 | 1.28 | 0.07 |
| C70-H74 | σ | C71-C72 | σ* | 4.97 | 1.05 | 0.07 |
| C10-C20 | σ | C7-C20 | σ* | 4.99 | 1.23 | 0.07 |
| C68-S76 | σ | C25-C37 | σ* | 5.88 | 1.15 | 0.07 |
| C6-C8 | σ | C8-C9 | σ* | 5.89 | 1.16 | 0.07 |
| C2-C19 | σ | C1-C2 | σ* | 5.96 | 1.16 | 0.07 |
| C22-C23 | σ | C21-C22 | σ* | 6.48 | 1.32 | 0.08 |
| C13-C14 | σ | C5-C13 | σ* | 6.62 | 1.29 | 0.08 |
| C23-C37 | σ | C25-S76 | σ* | 6.80 | 0.88 | 0.07 |
| C37-C63 | π | C22-C23 | π* | 7.60 | 0.33 | 0.05 |
| C2-C3 | σ | C4-S77 | σ* | 7.82 | 0.91 | 0.08 |
| C2-C3 | σ | C19-S79 | σ* | 8.56 | 0.9 | 0.07 |
| C10-C20 | σ | C12-S81 | σ* | 8.62 | 0.92 | 0.08 |
| C102-N103 | σ | C72-C102 | σ* | 8.62 | 1.57 | 0.11 |
| C71-C100 | σ | C100-N101 | σ* | 8.89 | 1.62 | 0.11 |
| C72-C102 | σ | C102-N103 | σ* | 8.93 | 1.62 | 0.11 |
| C37-C63 | π | C25-C26 | π* | 9.48 | 0.32 | 0.05 |
| C22-C23 | π | C21-C82 | π* | 11.83 | 0.30 | 0.06 |
| C15-C16 | π | C27-C28 | π* | 11.84 | 0.32 | 0.06 |
| C10-C20 | π | C7-C8 | π* | 14.09 | 0.31 | 0.06 |
| C2-C3 | π | C4-C5 | π* | 14.40 | 0.31 | 0.06 |
| C7-C8 | π | C6-C19 | π* | 15.00 | 0.28 | 0.06 |
| C4-C5 | π | C13-C14 | π* | 15.48 | 0.29 | 0.06 |
| C15-C16 | π | C13-C14 | π* | 15.74 | 0.29 | 0.07 |
| C21-C82 | π | C11-C12 | π* | 15.76 | 0.27 | 0.06 |
| C4-C5 | π | C2-C3 | π* | 15.87 | 0.31 | 0.07 |
| C27-C28 | π | C15-C16 | π* | 16.27 | 0.28 | 0.06 |
| C13-C14 | π | C15-C16 | π* | 16.54 | 0.32 | 0.07 |
| C11-C12 | π | C10-C20 | π* | 17.56 | 0.31 | 0.07 |
| C2-C3 | π | C6-C19 | π* | 17.64 | 0.28 | 0.07 |
| C6-C19 | π | C2-C3 | π* | 18.22 | 0.31 | 0.07 |
| C25-C26 | π | C69-C70 | π* | 18.68 | 0.32 | 0.07 |
| C13-C14 | π | C4-C5 | π* | 18.75 | 0.32 | 0.07 |
| C7-C8 | π | C10-C20 | π* | 18.81 | 0.30 | 0.07 |
| C29-C32 | π | C27-C28 | π* | 19.10 | 0.30 | 0.07 |
| C30-C34 | π | C29-C32 | π* | 19.47 | 0.30 | 0.07 |
| C25-C26 | π | C37-C63 | π* | 20.19 | 0.32 | 0.07 |
| C25-C26 | π | C38-O62 | π* | 20.24 | 0.33 | 0.07 |
| C68-C73 | π | C69-C70 | π* | 20.38 | 0.31 | 0.07 |
| C42-C45 | π | C43-C47 | π* | 20.39 | 0.30 | 0.07 |
| C40-C41 | π | C42-C45 | π* | 20.40 | 0.30 | 0.07 |
| C51-C52 | π | C53-C56 | π* | 20.50 | 0.31 | 0.07 |
| C69-C70 | π | C68-C73 | π* | 20.66 | 0.28 | 0.07 |
| C53-C56 | π | C54-C58 | π* | 20.83 | 0.30 | 0.07 |
| C10-C20 | π | C11-C12 | π* | 20.87 | 0.27 | 0.07 |
| C69-C70 | π | C25-C26 | π* | 20.93 | 0.29 | 0.07 |
| C68-C73 | π | C71-C72 | π* | 20.96 | 0.29 | 0.07 |
| C27-C28 | π | C30-C34 | π* | 21.77 | 0.29 | 0.07 |
| C43-C47 | π | C40-C41 | π* | 21.79 | 0.29 | 0.07 |
| C29-C32 | π | C30-C34 | π* | 21.80 | 0.30 | 0.07 |
| C6-C19 | π | C7-C8 | π* | 21.91 | 0.31 | 0.08 |
| C27-C28 | π | C29-C32 | π* | 21.92 | 0.30 | 0.07 |
| C54-C58 | π | C51-C52 | π* | 22.01 | 0.29 | 0.07 |
| C71-C72 | π | C69-C70 | π* | 22.28 | 0.31 | 0.07 |
| C51-C52 | π | C54-C58 | π* | 22.81 | 0.30 | 0.07 |
| C42-C45 | π | C40-C41 | π* | 23.03 | 0.29 | 0.08 |
| C40-C41 | π | C43-C47 | π* | 23.11 | 0.30 | 0.08 |
| C53-C56 | π | C51-C52 | π* | 23.16 | 0.29 | 0.08 |
| C54-C58 | π | C53-C56 | π* | 23.32 | 0.30 | 0.08 |
| C22-C23 | π | C38-O62 | π* | 23.38 | 0.30 | 0.08 |
| C43-C47 | π | C42-C45 | π* | 23.61 | 0.30 | 0.08 |
| C71-C72 | π | C68-C73 | π* | 23.95 | 0.29 | 0.07 |
| C30-C34 | π | C27-C28 | π* | 24.31 | 0.30 | 0.08 |
| C69-C70 | π | C71-C72 | π* | 25.35 | 0.27 | 0.08 |
| C11-C12 | π | C21-C82 | π* | 26.06 | 0.30 | 0.08 |
| C22-C23 | π | C37-C63 | π* | 28.39 | 0.29 | 0.08 |
| C21-C82 | π | C22-C23 | π* | 31.36 | 0.30 | 0.09 |
| S80 | LP(1) | C11-C82 | σ* | 0.63 | 1.25 | 0.03 |
| S76 | LP(1) | C68-C69 | σ* | 2.94 | 1.20 | 0.05 |
| S80 | LP(1) | C10-C20 | σ* | 2.98 | 1.21 | 0.05 |
| N39 | LP(1) | C51-C53 | σ* | 3.57 | 0.85 | 0.05 |
| N39 | LP(1) | C51-C52 | σ* | 3.73 | 0.85 | 0.05 |
| N101 | LP(1) | C71-C100 | σ* | 12.30 | 1.05 | 0.10 |
| N103 | LP(1) | C72-C102 | σ* | 12.33 | 1.05 | 0.10 |
| N65 | LP(1) | C63-C64 | σ* | 12.65 | 1.04 | 0.10 |
| N67 | LP(1) | C63-C66 | σ* | 12.67 | 1.04 | 0.10 |
| N39 | LP(1) | C51-C52 | π* | 16.17 | 0.30 | 0.06 |
| S81 | LP(2) | C21-C82 | π* | 18.28 | 0.27 | 0.06 |
| N39 | LP(1) | C40-C41 | π* | 18.65 | 0.30 | 0.07 |
| O62 | LP(2) | C23-C38 | σ* | 19.65 | 0.74 | 0.11 |
| O62 | LP(2) | C26-C38 | σ* | 21.23 | 0.75 | 0.11 |
| S80 | LP(2) | C11-C12 | π* | 22.22 | 0.25 | 0.07 |
| S79 | LP(2) | C7-C8 | π* | 22.75 | 0.28 | 0.07 |
| S17 | LP(2) | C15-C16 | π* | 23.55 | 0.28 | 0.07 |
| S78 | LP(2) | C6-C19 | π* | 23.58 | 0.25 | 0.07 |
| S76 | LP(2) | C68-C73 | π* | 24.04 | 0.27 | 0.07 |
| S77 | LP(2) | C13-C14 | π* | 24.14 | 0.26 | 0.07 |
| S77 | LP(2) | C4-C5 | π* | 24.21 | 0.28 | 0.08 |
| S17 | LP(2) | C13-C14 | π* | 24.61 | 0.26 | 0.07 |
| S78 | LP(2) | C2-C3 | π* | 24.77 | 0.28 | 0.08 |
| S79 | LP(2) | C6-C19 | π* | 24.86 | 0.25 | 0.08 |
| N39 | LP(1) | C30-C34 | π* | 25.60 | 0.29 | 0.08 |
| S76 | LP(2) | C25-C26 | π* | 26.75 | 0.28 | 0.08 |
| S80 | LP(2) | C10-C20 | π* | 26.90 | 0.28 | 0.08 |
| S81 | LP(2) | C11-C12 | π* | 28.12 | 0.24 | 0.08 |

**Table S13:** Wavelength, excitation energy and oscillator strength of **FICR** at M06/6-311G(d,p) level in the gaseous phase.

| **NO** | **DFT *λ* (*nm*)** | ***E*(*eV*)** | ***f*_os_** | **MO contributions** |
| --- | --- | --- | --- | --- |
| 1 | 701 | 1.77 | 1.98 | H→L (97%), |
| 2 | 544 | 2.28 | 0.51 | H-1→L (89%), H-2→L (3%), H→L (2%), H→L+1 (2%), H→L+2 (3%) |
| 3 | 527 | 2.35 | 0.21 | H→L+1 (86%), H-1→L (3%), H-1→L+1 (6%) |
| 4 | 457 | 2.71 | 0.40 | H-2→L (42%), H→L+2 (47%), H-1→L (6%) |
| 5 | 433 | 2.86 | 0.09 | H-2→L (50%), H→L+2 (42%), |
| 6 | 425 | 2.92 | 0.02 | H-1→L+1 (77%), H-2→L+1 (8%), H→L+1 (9%) |

MO=molecular orbital, H=HOMO, L=LUMO, *f*_os_*=* oscillator strength, $\lambda$ (*nm*)= wavelength

**Table S14:** Wavelength, excitation energy and oscillator strength of **FICD1** at M06/6-311G(d,p) level in the gaseous phase.

| **NO** | **DFT *λ* (*nm*)** | ***E*(*eV*)** | ***f*_os_** | **MO contributions** |
| --- | --- | --- | --- | --- |
| 1 | 734 | 1.69 | 1.19 | H→L (94%), H-1→L (3%) |
| 2 | 588 | 2.11 | 1.13 | H→L+1 (88%), H-1→L (2%), H-1→L+1 (5%) |
| 3 | 559 | 2.22 | 0.34 | H-1→L (86%), H-2→L (5%), H→L (4%), H→L+1 (2%) |
| 4 | 467 | 2.66 | 0.02 | H-2→L (56%), H-1→L+1 (21%), H-4→L (3%), H-2→L+1 (2%), H-1→L (7%), H→L+2 (4%) |
| 5 | 459 | 2.70 | 0.31 | H-2→L (16%), H-1→L+1 (58%), H→L+2 (12%), H-2→L+1 (4%), H→L+1 (5%) |
| 6 | 433 | 2.87 | 0.28 | H-2→L (11%), H→L+2 (75%), H-1→L+1 (7%) |

MO=molecular orbital, H=HOMO, L=LUMO, *f*_os_*=* oscillator strength, $\lambda$ (*nm*)= wavelength

**Table S15:** Wavelength, excitation energy and oscillator strength of **FICD2** at M06/6-311G(d,p) level in the gaseous phase.

| **NO** | **DFT *λ* (*nm*)** | ***E*(*eV*)** | ***f*_os_** | **MO contributions** |
| --- | --- | --- | --- | --- |
| 1 | 815 | 1.52 | 0.93 | H→L (95%), H-1→L (3%) |
| 2 | 626 | 1.98 | 0.02 | H-1→L (74%), H→L+1 (17%), H-2→L (2%), H→L (4%) |
| 3 | 615 | 2.02 | 1.56 | H-1→L (18%), H→L+1 (74%), H-1→L+1 (4%) |
| 4 | 514 | 2.41 | 0.06 | H→L+2 (84%), H-2→L (7%), H-1→L+2 (3%) |
| 5 | 494 | 2.51 | 0.00 | H-2→L (65%), H-1→L+1 (13%), H→L+2 (12%), H-1→L (3%) |
| 6 | 489 | 2.54 | 0.29 | H-2→L (13%), H-1→L+1 (72%), H-2→L+1 (3%), H→L+1 (6%), H→L+4 (3%) |

MO=molecular orbital, H=HOMO, L=LUMO, *f*_os_*=* oscillator strength, $\lambda$ (*nm*)= wavelength

**Table S16:** Wavelength, excitation energy and oscillator strength of **FICD3** at M06/6-311G(d,p) level in the gaseous phase.

| **NO** | **DFT *λ* (*nm*)** | ***E*(*eV*)** | ***f*_os_** | **MO contributions** |
| --- | --- | --- | --- | --- |
| 1 | 765 | 1.62 | 1.08 | H→L (95%), H-1→L (3%) |
| 2 | 602 | 2.06 | 1.01 | H→L+1 (86%), H-1→L (5%), H-1→L+1 (5%) |
| 3 | 583 | 2.13 | 0.49 | H-1→L (84%), H-2→L (4%), H→L (4%), H→L+1 (5%) |
| 4 | 477 | 2.59 | 0.01 | H-2→L (49%), H-1→L+1 (33%), H-2→L+1 (2%), H-1→L (4%), H→L+1 (3%) |
| 5 | 471 | 2.63 | 0.34 | H-2→L (29%), H-1→L+1 (49%), H→L+2 (10%), H-2→L+1 (3%), H-1→L (2%), H→L+1 (5%) |
| 6 | 437 | 2.84 | 0.33 | H→L+2 (79%), H-2→L (9%), H-1→L+1 (5%) |

MO=molecular orbital, H=HOMO, L=LUMO, *f*_os_*=* oscillator strength, $\lambda$ (*nm*)= wavelength

**Table S17:** Wavelength, excitation energy and oscillator strength of **FICD4** at M06/6-311G(d,p) level in the gaseous phase.

| **NO** | **DFT *λ* (*nm*)** | ***E*(*eV*)** | ***f*_os_** | **MO contributions** |
| --- | --- | --- | --- | --- |
| 1 | 725 | 1.71 | 1.28 | H→L (94%), H-1→L (3%) |
| 2 | 584 | 2.12 | 1.08 | H→L+1 (89%), H-1→L+1 (5%) |
| 3 | 553 | 2.24 | 0.33 | H-1→L (86%), H-2→L (5%), H→L (4%) |
| 4 | 463 | 2.68 | 0.02 | H-2→L (48%), H-1→L+1 (28%), H-4→L (3%), H-2→L+1 (3%), H-1→L (6%), H→L+1 (2%), H→L+2 (4%) |
| 5 | 456 | 2.72 | 0.34 | H-2→L (19%), H-1→L+1 (49%), H→L+2 (18%), H-2→L+1 (4%), H-1→L (2%), H→L+1 (5%) |
| 6 | 434 | 2.86 | 0.22 | H-2→L (17%), H→L+2 (69%), H-1→L+1 (8%) |

MO=molecular orbital, H=HOMO, L=LUMO, *f*_os_*=* oscillator strength, $\lambda$ (*nm*)= wavelength

**Table S18:** Wavelength, excitation energy and oscillator strength of **FICD5** at M06/6-311G(d,p) level in the gaseous phase.

| **NO** | **DFT *λ* (*nm*)** | ***E*(*eV*)** | ***f*_os_** | **MO contributions** |
| --- | --- | --- | --- | --- |
| 1 | 813 | 1.53 | 0.97 | H→L (95%), H-1→L (3%) |
| 2 | 622 | 1.99 | 0.86 | H-1→L (12%), H→L+1 (79%), H-1→L+1 (4%) |
| 3 | 610 | 2.03 | 0.75 | H-1→L (78%), H→L+1 (13%), H-2→L (3%), H→L (3%) |
| 4 | 490 | 2.53 | 0.01 | H-2→L (48%), H-1→L+1 (36%), H-2→L+1 (2%), H-1→L (4%), H→L+1 (2%) |
| 5 | 485 | 2.56 | 0.37 | H-2→L (32%), H-1→L+1 (47%), H-2→L+1 (2%), H-1→L (2%), H→L+1 (4%), H→L+2 (4%), H→L+3 (5%) |
| 6 | 444 | 2.79 | 0.31 | H→L+2 (51%), H→L+3 (30%), H-2→L (8%), H-1→L+1 (4%) |

MO=molecular orbital, H=HOMO, L=LUMO, *f*_os_*=* oscillator strength, $\lambda$ (*nm*)= wavelength

**Table S19:** Wavelength, excitation energy and oscillator strength of **FICR** at M06/6-311G(d,p) level in the solvent phase.

| **NO** | **DFT λ (nm)** | **E(eV)** | ***f*_os_** | **MO contributions** |
| --- | --- | --- | --- | --- |
| 1 | 771 | 1.61 | 2.20 | H→L (94%), H-1→L (3%) |
| 2 | 569 | 2.18 | 0.42 | H-1→L (80%), H-2→L (4%), H→L (5%), H→L+1 (8%) |
| 3 | 561 | 2.21 | 0.17 | H-1→L (10%), H→L+1 (80%), H-1→L+1 (5%) |
| 4 | 467 | 2.65 | 0.38 | H-2→L (60%), H→L+2 (29%), H-1→L (6%) |
| 5 | 453 | 2.74 | 0.24 | H-2→L (31%), H→L+2 (61%), |
| 6 | 442 | 2.81 | 0.03 | H-1→L+1 (78%), H-2→L+1 (8%), H→L+1 (9%) |

MO=molecular orbital, H=HOMO, L=LUMO, *f*_os_*=* oscillator strength, $\lambda$ (*nm*)= wavelength

**Table S20:** Wavelength, excitation energy and oscillator strength of **FICD1** at M06/6-311G(d,p) level in the solvent phase.

| **NO** | **DFT λ (nm)** | **E(eV)** | ***f*_os_** | **MO contributions** |
| --- | --- | --- | --- | --- |
| 1 | 806 | 1.54 | 1.34 | H→L (93%), H-1→L (4%) |
| 2 | 618 | 2.01 | 1.29 | H→L+1 (90%), H-1→L+1 (5%) |
| 3 | 585 | 2.12 | 0.16 | H-1→L (85%), H-2→L (7%), H→L (6%) |
| 4 | 483 | 2.57 | 0.04 | H-2→L (74%), H-5→L (2%), H-4→L (2%), H-1→L (9%), H-1→L+1 (5%) |
| 5 | 468 | 2.65 | 0.12 | H-1→L+1 (77%), H-2→L (6%), H-2→L+1 (7%), H→L+1 (7%) |
| 6 | 447 | 2.78 | 0.48 | H→L+2 (90%), H-2→L (3%) |

MO=molecular orbital, H=HOMO, L=LUMO, *f*_os_*=* oscillator strength, $\lambda$ (*nm*)= wavelength

**Table S21:** Wavelength, excitation energy and oscillator strength of **FICD2** at M06/6-311G(d,p) level in the solvent phase.

| **NO** | **DFT λ (nm)** | **E(eV)** | ***f*_os_** | **MO contributions** |
| --- | --- | --- | --- | --- |
| 1 | 889 | 1.39 | 1.19 | H→L (94%), H-1→L (4%) |
| 2 | 642 | 1.93 | 0.68 | H-1→L (24%), H→L+1 (64%), H-1→L+1 (4%), H→L+2 (3%) |
| 3 | 641 | 1.93 | 0.83 | H-1→L (64%), H→L+1 (20%), H-2→L (3%), H→L (4%), H→L+2 (5%) |
| 4 | 577 | 2.15 | 0.07 | H→L+2 (86%), H-1→L+2 (2%), H→L+1 (7%) |
| 5 | 512 | 2.42 | 0.04 | H-2→L (85%), H-1→L (6%) |
| 6 | 488 | 2.54 | 0.11 | H-1→L+1 (78%), H-2→L+1 (5%), H-1→L+2 (6%), H→L+1 (7%) |

MO=molecular orbital, H=HOMO, L=LUMO, *f*_os_*=* oscillator strength, $\lambda$ (*nm*)= wavelength

**Table S22:** Wavelength, excitation energy and oscillator strength of **FICD3** at M06/6-311G(d,p) level in the solvent phase.

| **NO** | **DFT λ (nm)** | **E(eV)** | ***f*_os_** | **MO contributions** |
| --- | --- | --- | --- | --- |
| 1 | 832 | 1.49 | 1.29 | H→L (93%), H-1→L (4%) |
| 2 | 628 | 1.98 | 1.29 | H→L+1 (90%), H-1→L+1 (5%) |
| 3 | 604 | 2.05 | 0.16 | H-1→L (87%), H-2→L (6%), H→L (6%) |
| 4 | 490 | 2.53 | 0.05 | H-2→L (79%), H-6→L (2%), H-1→L (8%), H-1→L+1 (3%) |
| 5 | 477 | 2.60 | 0.13 | H-1→L+1 (80%), H-2→L (4%), H-2→L+1 (6%), H→L+1 (7%) |
| 6 | 450 | 2.76 | 0.49 | H→L+2 (89%), H-2→L (3%) |

MO=molecular orbital, H=HOMO, L=LUMO, *f*_os_*=* oscillator strength, $\lambda$ (*nm*)= wavelength

**Table S23:** Wavelength, excitation energy and oscillator strength of **FICD4** at M06/6-311G(d,p) level in the solvent phase.

| **NO** | **DFT λ (nm)** | **E(eV)** | ***f*_os_** | **MO contributions** |
| --- | --- | --- | --- | --- |
| 1 | 806 | 1.54 | 1.36 | H→L (93%), H-1→L (4%) |
| 2 | 619 | 2.00 | 1.26 | H→L+1 (89%), H-1→L+1 (6%) |
| 3 | 586 | 2.12 | 0.16 | H-1→L (85%), H-2→L (6%), H→L (6%) |
| 4 | 481 | 2.58 | 0.04 | H-2→L (72%), H-1→L (8%), H-1→L+1 (7%), H→L+2 (3%) |
| 5 | 470 | 2.64 | 0.14 | H-1→L+1 (75%), H-2→L (7%), H-2→L+1 (6%), H→L+1 (7%) |
| 6 | 450 | 2.76 | 0.47 | H→L+2 (87%), H-2→L (4%), H→L+3 (2%) |

MO=molecular orbital, H=HOMO, L=LUMO, *f*_os_*=* oscillator strength, $\lambda$ (*nm*)= wavelength

**Table S24:** Wavelength, excitation energy and oscillator strength of **FICD5** at M06/6-311G(d,p) level in the solvent phase.

| **NO** | **DFT λ (nm)** | **E(eV)** | ***f*_os_** | **MO contributions** |
| --- | --- | --- | --- | --- |
| 1 | 880 | 1.41 | 1.23 | H→L (94%), H-1→L (4%) |
| 2 | 644 | 1.92 | 1.39 | H→L+1 (90%), H-1→L+1 (5%) |
| 3 | 630 | 1.97 | 0.16 | H-1→L (88%), H-2→L (5%), H→L (5%) |
| 4 | 502 | 2.47 | 0.05 | H-2→L (81%), H-6→L (2%), H-1→L (7%) |
| 5 | 487 | 2.55 | 0.15 | H-1→L+1 (82%), H-2→L+1 (6%), H→L+1 (7%) |
| 6 | 460 | 2.69 | 0.35 | H→L+2 (54%), H→L+3 (37%), H-2→L (3%) |

MO=molecular orbital, H=HOMO, L=LUMO, *f*_os_*=* oscillator strength, $\lambda$ (*nm*)= wavelength

**Table S25**: Dipole polarizability and major contributing tensor (*esu*) of the studied compounds **FICR** and **FICD1-FICD5**.

| Dipole Moment |  |  |  |  |  |  |
| --- | --- | --- | --- | --- | --- | --- |
|  | **FICR** | **FICD1** | **FICD2** | **FICD3** | **FICD4** | **FICD5** |
| ***µ_x_*** | 17.36 | 13.86 | 19.23 | 16.14 | 16.19 | 19.47 |
| ***µ_y_*** | 4.09 | 3.57 | -2.60 | 1.32 | 6.69 | -3.32 |
| ***µ_z_*** | -1.15 | 0.04 | -0.36 | -0.04 | -1.63 | -0.59 |
| ***µ*_total_** | 17.87 | 14.31 | 19.41 | 16.20 | 17.59 | 19.76 |
| Polarizability |  |  | |  |  |  |
| ***α_xx_*** | 5.54×10^-22^ | 5.42×10^-22^ | 5.88×10^-22^ | 5.53×10^-22^ | 5.47×10^-22^ | 5.94×10^-22^ |
| ***α_yy_*** | 1.61×10^-22^ | 1.8×10^-22^ | 1.78×10^-22^ | 1.76×10^-22^ | 1.88×10^-22^ | 1.85×10^-22^ |
| ***α_zz_*** | 6.98×10^-22^ | 8.74×10^-23^ | 9.15×10^-23^ | 8.86×10^-23^ | 9.22×10^-23^ | 8.49×10^-23^ |
| ***α*_total_** | 2.61×10^-22^ | 2.69×10^-22^ | 2.86×10^-22^ | 2.73×10^-22^ | 2.76×10^-22^ | 2.88×10^-22^ |
| 2^nd^ Hyper pol. |  |  |  |  |  |  |
| ***γ_X_*** | 8.14×10^-32^ | 7.94×10^-32^ | 1.32×10^-31^ | 9.35×10^-32^ | 8.16×10^-32^ | 1.30×10^-31^ |
| ***γ_Y_*** | 1.43×10^-34^ | 3.99×10^-34^ | 1.04×10^-33^ | 8.05×10^-34^ | 7.70×10^-34^ | 5.77×10^-34^ |
| ***γ_Z_*** | 2.87×10^-35^ | 4.07×10^-35^ | 5.29×10^-35^ | 4.49×10^-35^ | 3.87×10^-35^ | 2.74×10^-35^ |
| *Average*<*γ*> | 8.16×10^-32^ | 7.94×10^-32^ | 1.33×10^-31^ | 9.44×10^-32^ | 8.24×10^-32^ | 1.30×10^-31^ |
| *Magnitude of γ* | 8.14×10^-32^ | 7.99×10^-32^ | 1.32×10^-31^ | 9.35×10^-32^ | 8.16×10^-32^ | 1.30×10^-31^ |

**Table S26**: Frequency dependent Second hyperpolarizability (*esu*) of studied compounds **FICR** and **FICD1-FICD5**.

| **Parameters** | **Frequency**  **ω** | **FICD1** | **FICR** | **FICD2** | **FICD3** | **FICD4** | **FICD5** |
| --- | --- | --- | --- | --- | --- | --- | --- |
| *γ*(−*ω,ω*,0,0) | 0.00 | 7.99×10^-32^ | 8.16×10^-32^ | 1.33×10^-31^ | 9.44×10^-32^ | 8.24×10^-32^ | 1.30×10^-31^ |
|  | 1907.21*nm* | 1.14×10^-31^ | 1.08×10^-31^ | 2.24×10^-31^ | 1.41×10^-31^ | 1.17×10^-31^ | 2.14×10^-31^ |
| *γ*(−*2ω,ω,ω*,0) | 0.00 | 7.99×10^-32^ | 8.16×10^-32^ | 1.33×10^-31^ | 9.44×10^-32^ | 8.24×10^-32^ | 1.30×10^-31^ |
|  | 1907.21*nm* | 1.17×10^-30^ | 6.02×10^-31^ | -2.75×10^-30^ | 3.77×10^-30^ | 1.24×10^-30^ | -3.37×10^-30^ |

**Table S27**: The computed first hyperpolarizability (*β*_tot_) and major contributing tensors (*esu*) of **FICR** and **FICD1-FICD5**.

| **Polarizability** | **FICR** | **FICD1** | **FICD2** | **FICD3** | **FICD4** | **FICD5** |
| --- | --- | --- | --- | --- | --- | --- |
| ***β_xxx_*** | 6.44×10^-27^ | 5.78×10^-27^ | 8.41×10^-27^ | 6.59×10^-27^ | 5.85×10^-27^ | 8.37×10^-27^ |
| ***β_xxy_*** | 4.66×10^-29^ | 3.47×10^-28^ | 2.98×10^-28^ | 5.73×10^-28^ | 5.35×10^-28^ | 2.75×10^-28^ |
| ***β_xyy_*** | -2.63×10^-29^ | -6.68×10^-29^ | 3.10×10^-29^ | 7.62×10^-30^ | -1.19×10^-29^ | -1.90×10^-29^ |
| ***β_yyy_*** | -1.16×10^-29^ | 2.18×10^-29^ | -3.41×10^-29^ | -8.11×10^-30^ | -7.64×10^-30^ | -2.00×10^-29^ |
| ***β_xxz_*** | -1.29×10^-28^ | 1.65×10^-29^ | 9.37×10^-29^ | 8.10×10^-29^ | 4.85×10^-29^ | 4.26×10^-29^ |
| ***β_yyz_*** | 2.13×10^-32^ | 4.30×10^-30^ | 3.17×10^-31^ | 2.86×10^-30^ | 2.23×10^-30^ | 9.54×10^-31^ |
| ***β_xzz_*** | 2.94×10^-31^ | -1.33×10^-29^ | -1.22×10^-29^ | -8.27×10^-30^ | -1.00×10^-29^ | -9.44×10^-30^ |
| ***β_yzz_*** | -7.93×10^-32^ | -2.15×10^-30^ | -3.14×10^-30^ | -2.34×10^-30^ | -3.29×10^-30^ | -1.68×10^-30^ |
| ***β_zzz_*** | 2.02×10^-31^ | -6.65×10^-31^ | 2.37×10^-31^ | -1.03×10^-31^ | -5.62×10^-31^ | 1.17×10^-31^ |
| ***β_total_*** | 6.42×10^-27^ | 5.70×10^-27^ | 8.43×10^-27^ | 6.61×10^-27^ | 5.85×10^-27^ | 8.35×10^-27^ |

**Table S28**: Frequency dependent First hyperpolarizability (*esu*) of studied compounds **FICR** and **FICD1-FICD5**.

|  | **Parameters** | **Frequency *ω*** | **FICR** | **FICD1** | **FICD2** | **FICD3** | **FICD4** | **FICD5** |
| --- | --- | --- | --- | --- | --- | --- | --- | --- |
| Static | *β* (−*ω;ω,*0,) | 0.000 | 6.42×10^-27^ | 5.71×10^-27^ | 8.43×10^-27^ | 6.61×10^-27^ | 5.85×10^-27^ | 8.35×10^-27^ |
|  | *β* (-2, *ω;ω,ω*) | 0.000 | 6.42×10^-27^ | 5.71×10^-27^ | 8.43×10^-27^ | 6.61×10^-27^ | 5.85×10^-27^ | 8.35×10^-27^ |
| Specific | *β* (−*ω;ω*,0) | 1907.21*nm* | 7.52×10^-27^ | 6.96×10^-27^ | 1.09×10^-26^ | 8.19×10^-27^ | 7.15×10^-27^ | 1.08×10^-26^ |
|  | *β* (−2*ω;ω,ω*) | 1907.21*nm* | 2.03×10^-26^ | 2.21×10^-26^ | 7.41×10^-26^ | 3.06×10^-26^ | 2.27×10^-26^ | 6.38×10^-26^ |

**Table S29:** Equations for the calculation of global reactivity descriptors.

| $IP=-E_{\mathrm{HOMO}}$ | (1) |
| --- | --- |
| $EA=-E_{\mathrm{LUMO}}$ | (2) |
| $X=\frac{\left[ IP+EA \right]}{2}$ | (3) |
| $\eta=IP-$ EA | (4) |
| $\mu=\frac{E_{\mathrm{HOMO}}{+E}_{\mathrm{LUMO}}}{2}$ | (5) |
| $\sigma=\frac{1}{\eta}$ | (6) |
| $\omega=\frac{\mu^{2}}{2\eta}$ | (7) |
| ${\Delta N}_{max}=\frac{- \mu}{\eta}$ | (8) |

| 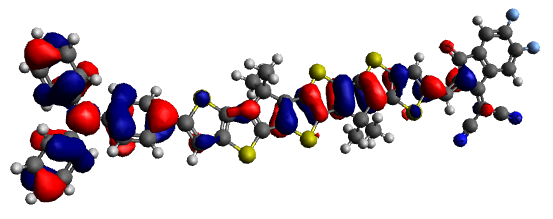 | 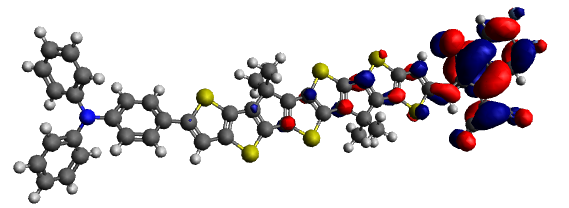 |
| --- | --- |
| **HOMO -1** | **LUMO +1** |
| 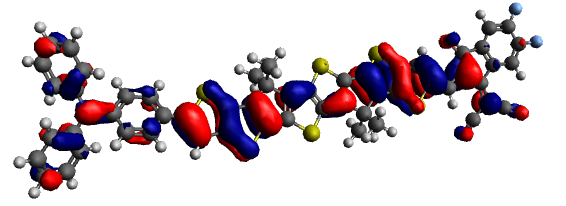 | 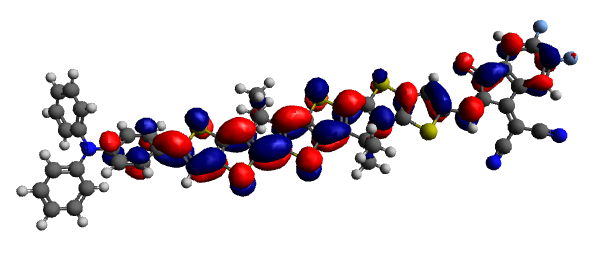 |
| **HOMO -2** | **LUMO +2** |
| **FICR** | |
| 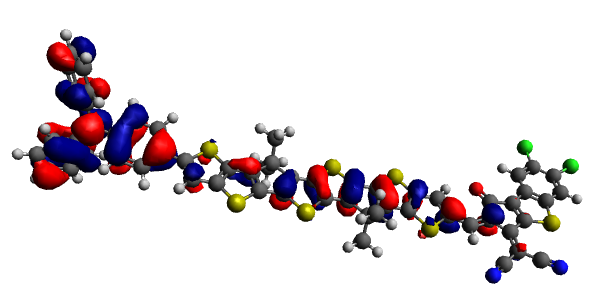 | 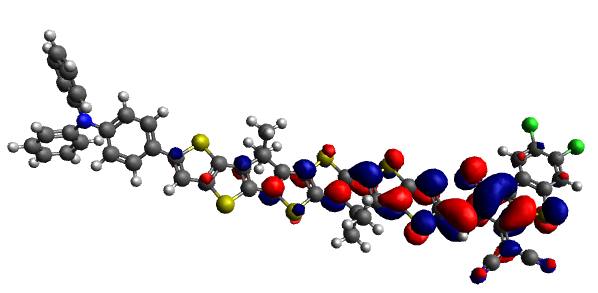 |
| **HOMO -1** | **LUMO +1** |
| 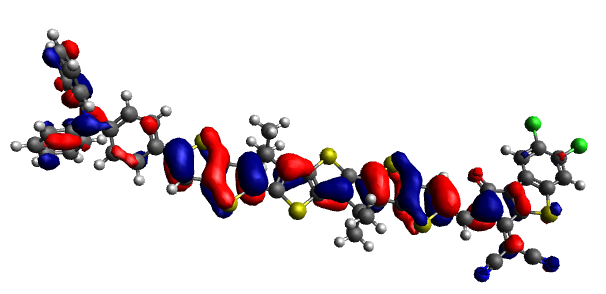 | 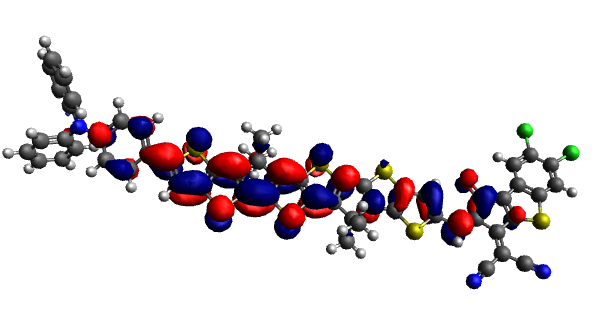 |
| **HOMO -2** | **LUMO +2** |
| **FICD1** | |
| 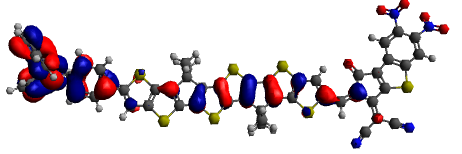 | 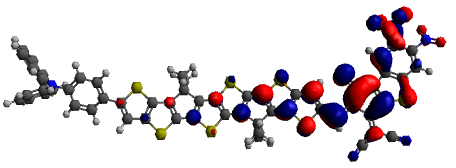 |
| **HOMO -1** | **LUMO +1** |
| 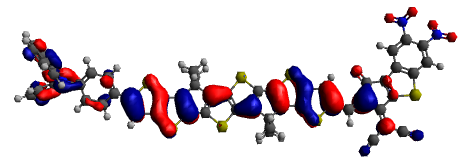 | 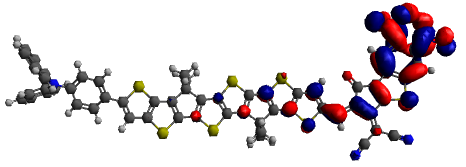 |
| **HOMO -2** | **LUMO +2** |
| **FICD2** | |
| 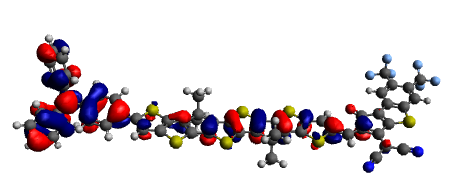 | 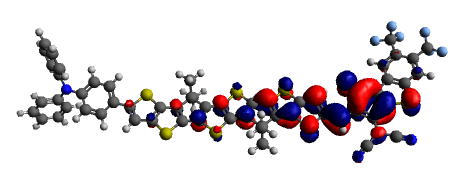 |
| **HOMO -1** | **LUMO +1** |
| 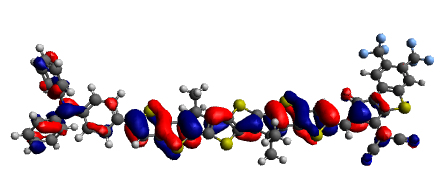 | 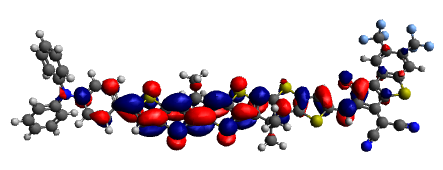 |
| **HOMO -2** | **LUMO +2** |
| **FICD3** | |
| 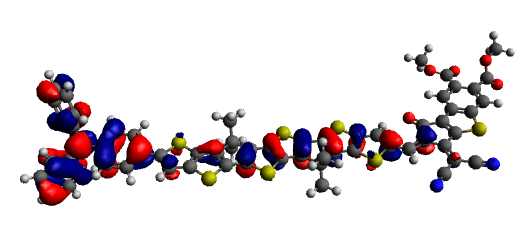 | 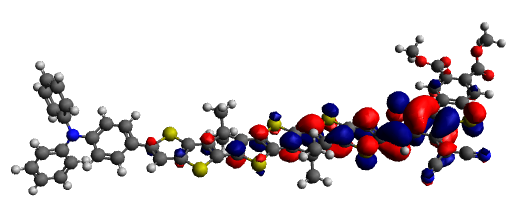 |
| **HOMO -1** | **LUMO +1** |
| 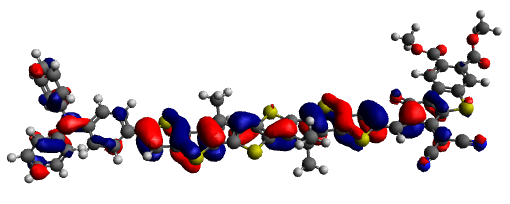 | 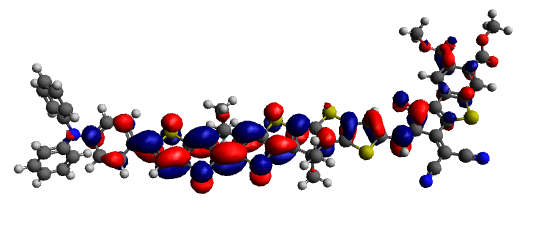 |
| **HOMO -2** | **LUMO +2** |
| **FICD4** | |
| 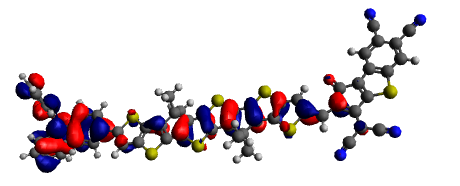 | 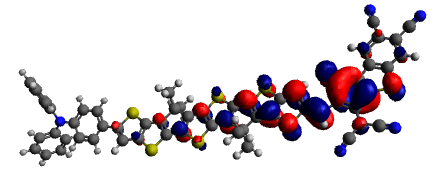 |
| **HOMO -1** | **LUMO +1** |
| 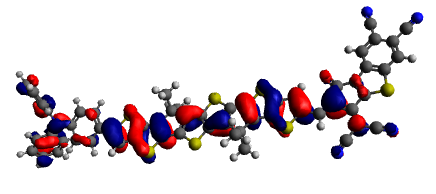 | 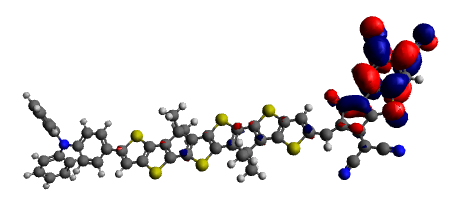 |
| **HOMO -2** | **LUMO +2** |
| **FICD5** | |

**Figure S1**: HOMO-1/LUMO+1 and HOMO-2/LUMO+2 of the studied compounds (**FICR** and **FICD1-FICD5**).
